# Supplementary material for: Interplay between acetylation and ubiquitination of imitation switch chromatin remodeler Isw1 confers multidrug resistance in Cryptococcus neoformans
Source: eLife. 2024 Jan 22;13:e85728. doi: 10.7554/eLife.85728 (PMC10834027; doi:10.7554/eLife.85728)
Supplement: Figure 1—figure supplement 1—source data 1. [file elife-85728-fig1-figsupp1-data1.zip › Figure 1-figure supplement 1-source data 1/Figure supplement 1-source data 4.pptx]

## Slide 1
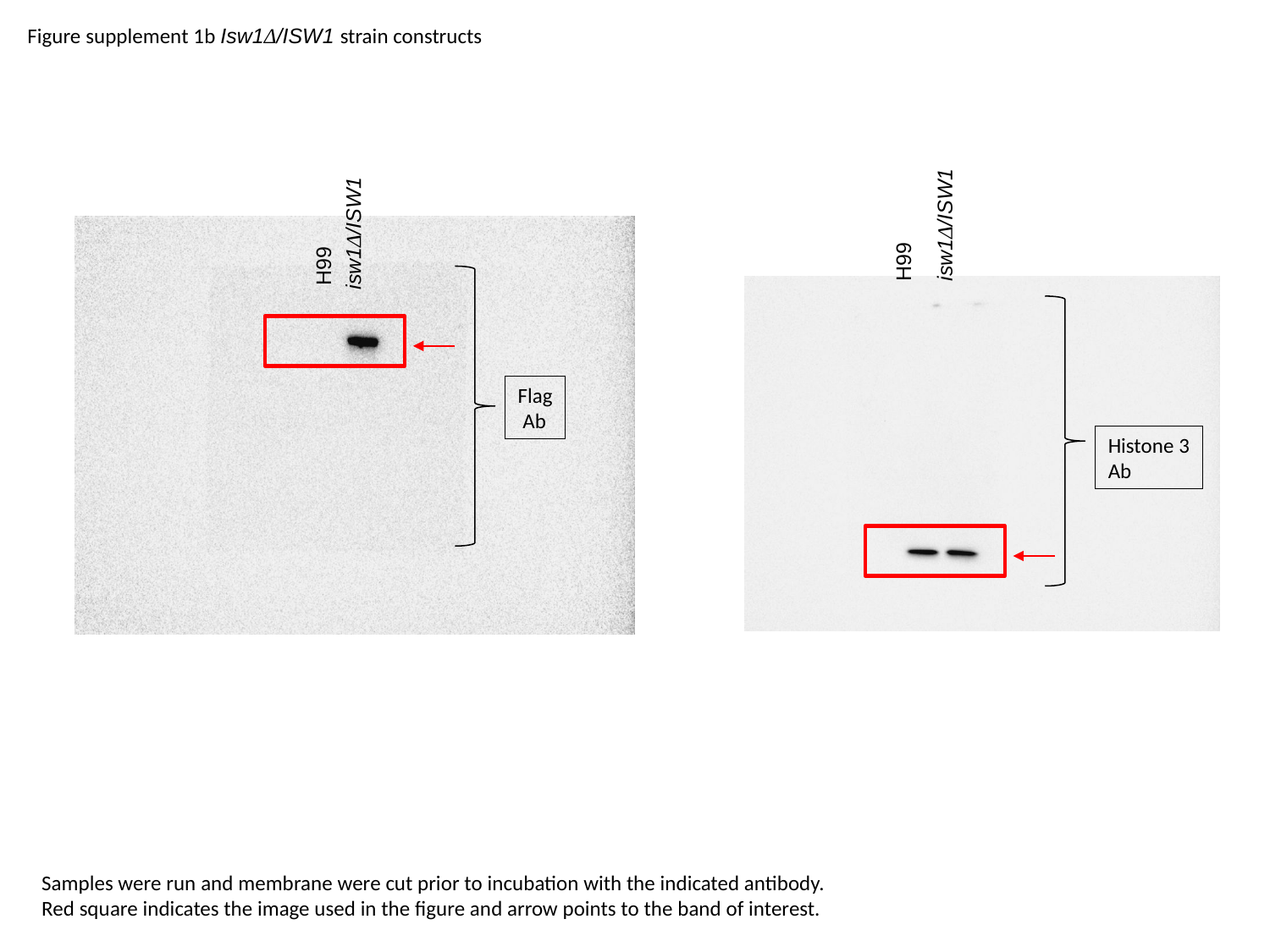

Figure supplement 1b Isw1Δ/ISW1 strain constructs
isw1Δ/ISW1
isw1Δ/ISW1
H99
H99
Flag
 Ab
Histone 3
Ab
Samples were run and membrane were cut prior to incubation with the indicated antibody.
Red square indicates the image used in the figure and arrow points to the band of interest.
