# Supplementary figures and images for: Interplay between acetylation and ubiquitination of imitation switch chromatin remodeler Isw1 confers multidrug resistance in Cryptococcus neoformans
Source: eLife. 2024 Jan 22;13:e85728. doi: 10.7554/eLife.85728 (PMC10834027; doi:10.7554/eLife.85728)

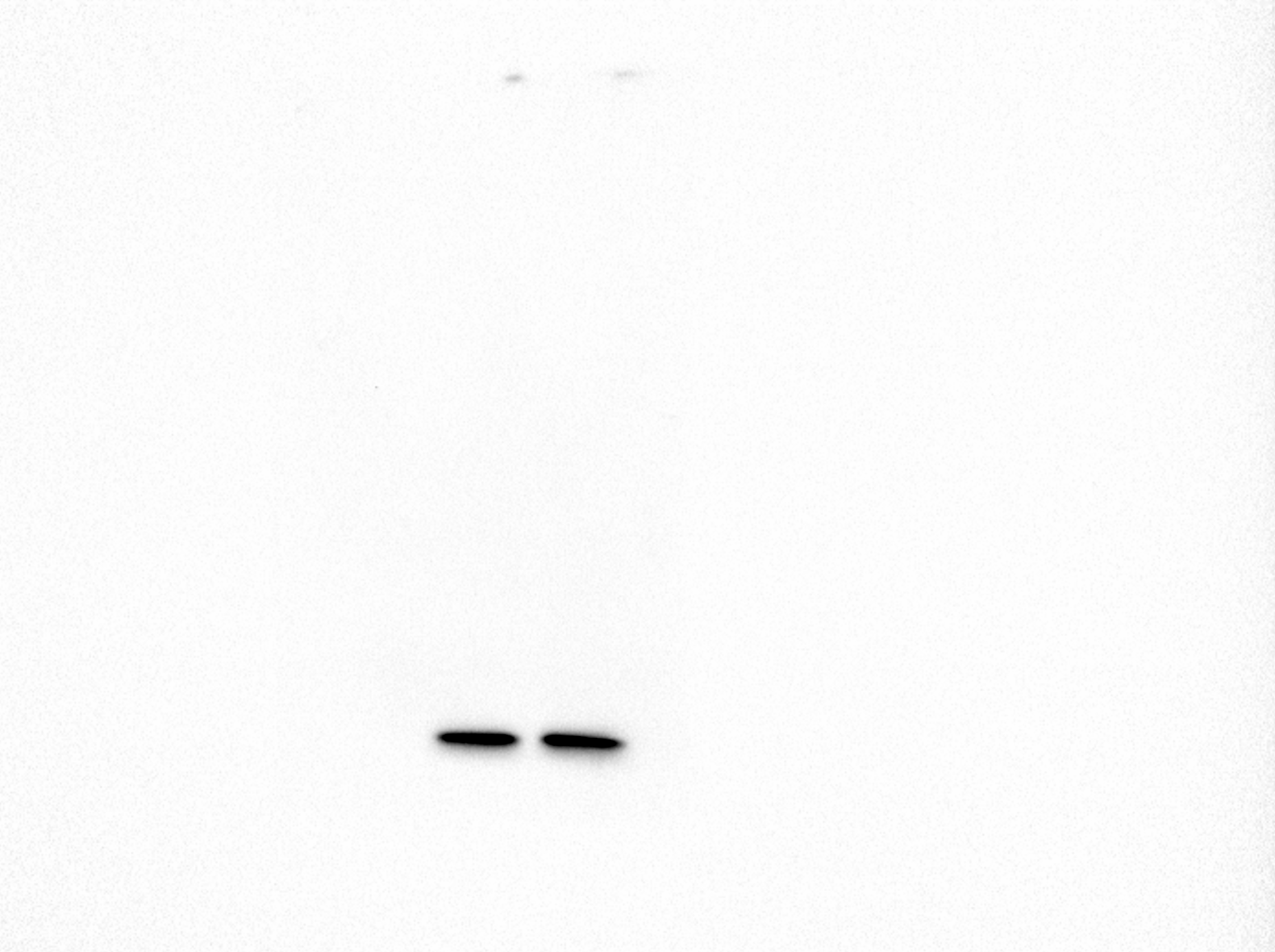

Supplement: Figure 1—figure supplement 1—source data 1. [file elife-85728-fig1-figsupp1-data1.zip › Figure 1-figure supplement 1-source data 1/Figure supplement 1b histone raw data.tif]

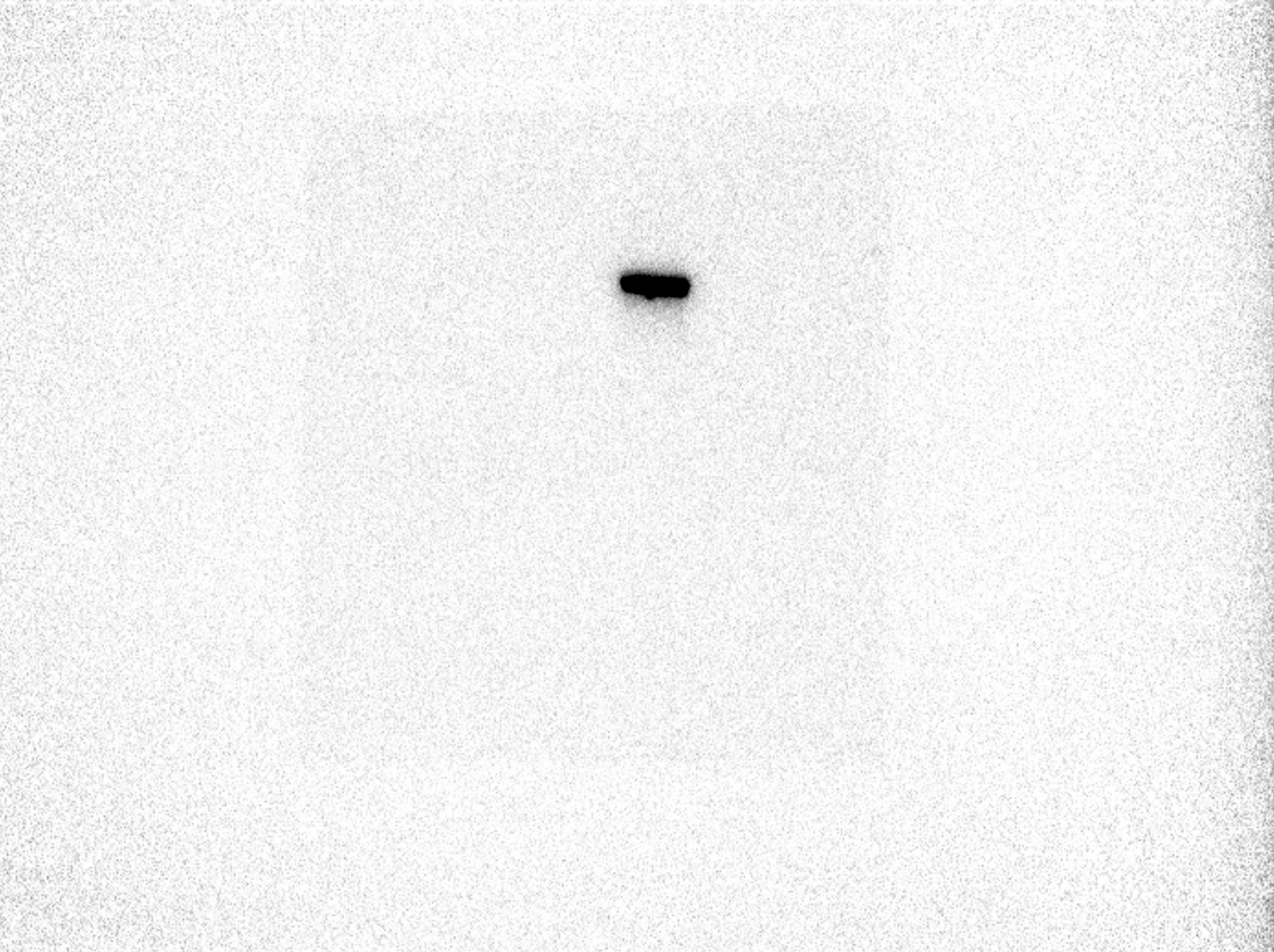

Supplement: Figure 1—figure supplement 1—source data 1. [file elife-85728-fig1-figsupp1-data1.zip › Figure 1-figure supplement 1-source data 1/Figure supplement 1b isw1-flag raw data.tif]

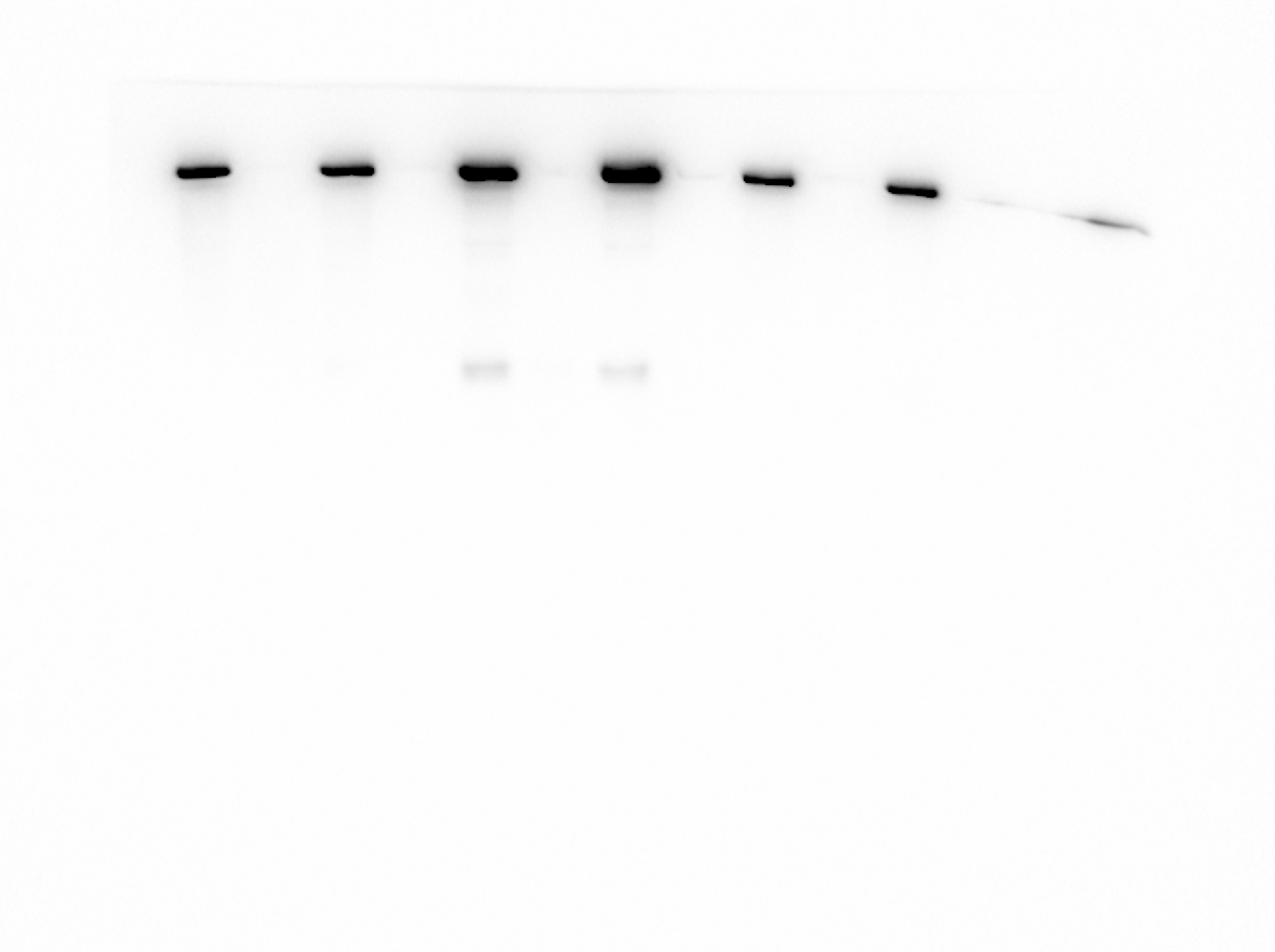

Supplement: Figure 2—source data 1. [file elife-85728-fig2-data1.zip › Figure 2-source data 1/Figure 2b CO-IP ISW1-ITC1-FLAG raw data.tif]

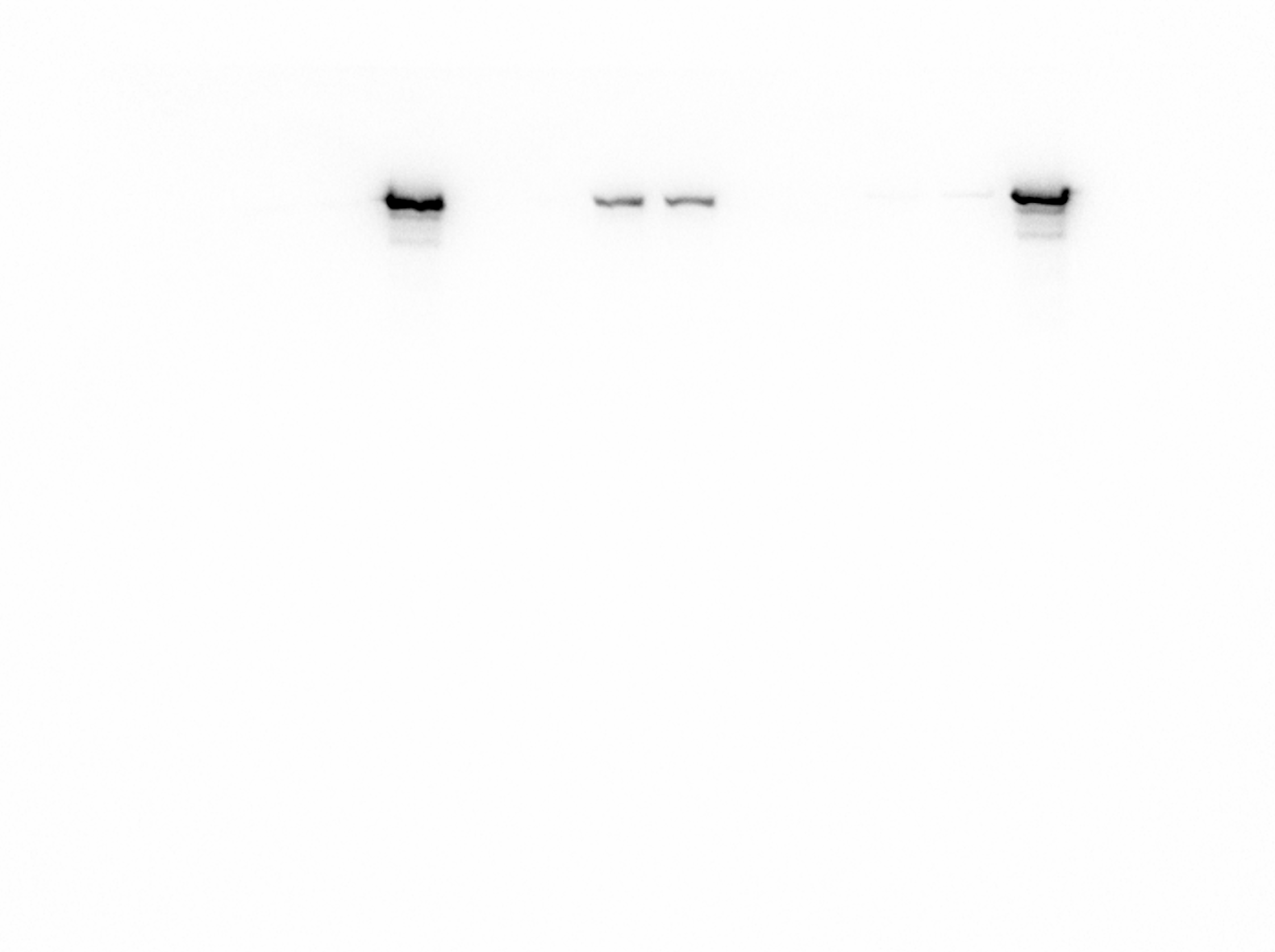

Supplement: Figure 2—source data 1. [file elife-85728-fig2-data1.zip › Figure 2-source data 1/Figure 2b CO-IP ISW1-ITC1-HA raw data.tif]

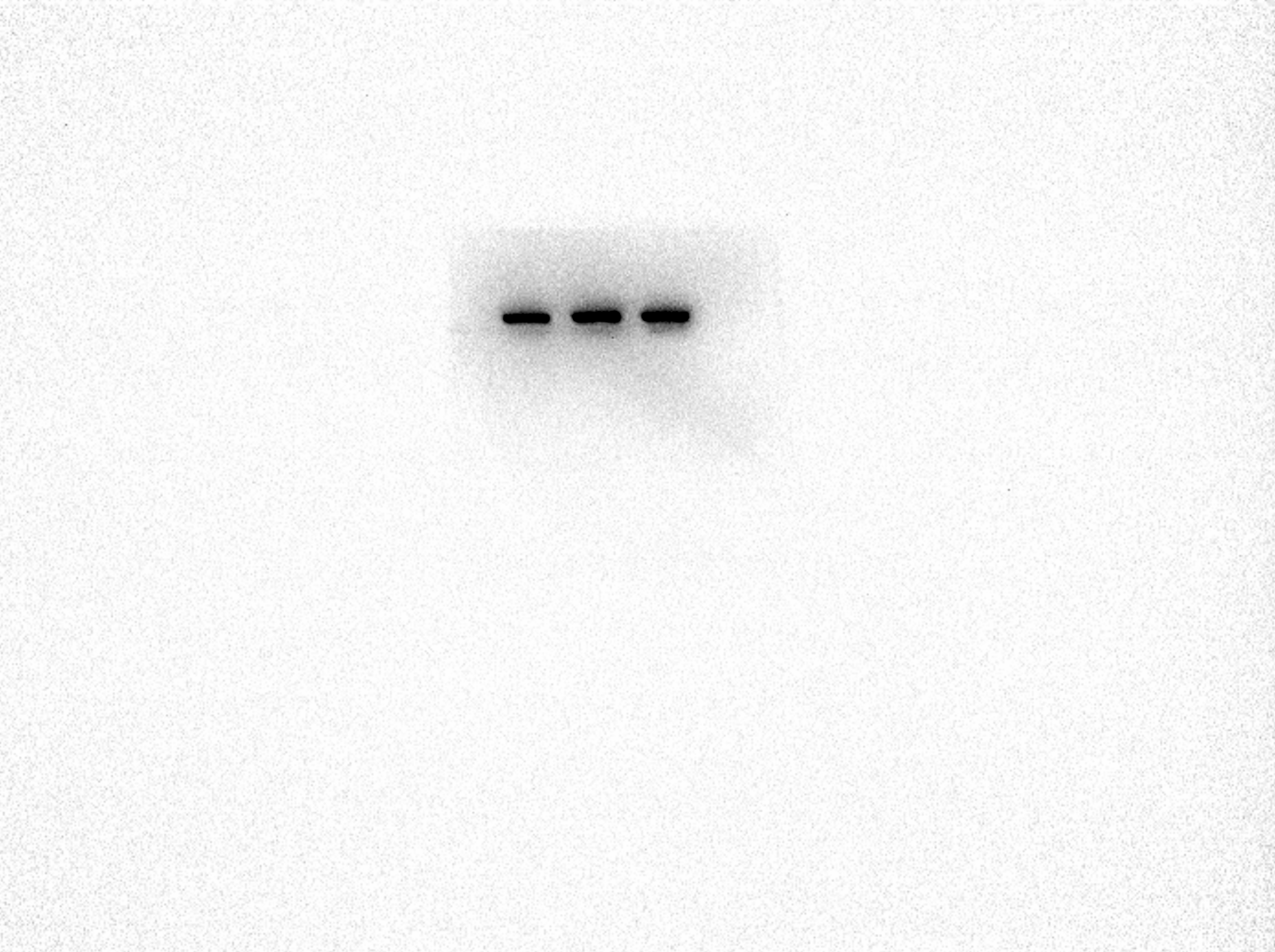

Supplement: Figure 2—source data 1. [file elife-85728-fig2-data1.zip › Figure 2-source data 1/Figure 2c CO-IP ISW1-CHD1 FLAG IP.tif]

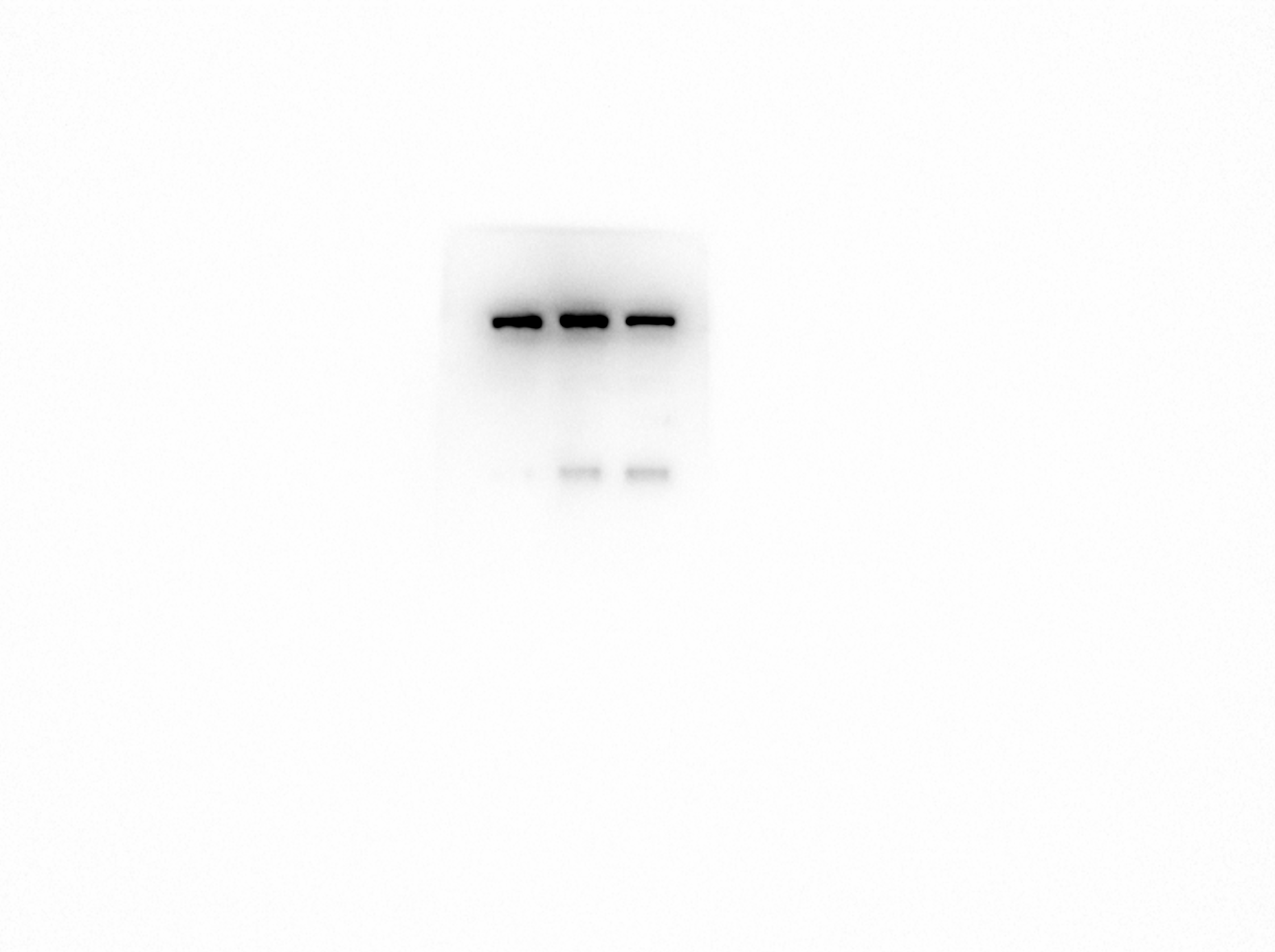

Supplement: Figure 2—source data 1. [file elife-85728-fig2-data1.zip › Figure 2-source data 1/Figure 2c CO-IP ISW1-CHD1 FLAG input raw data.tif]

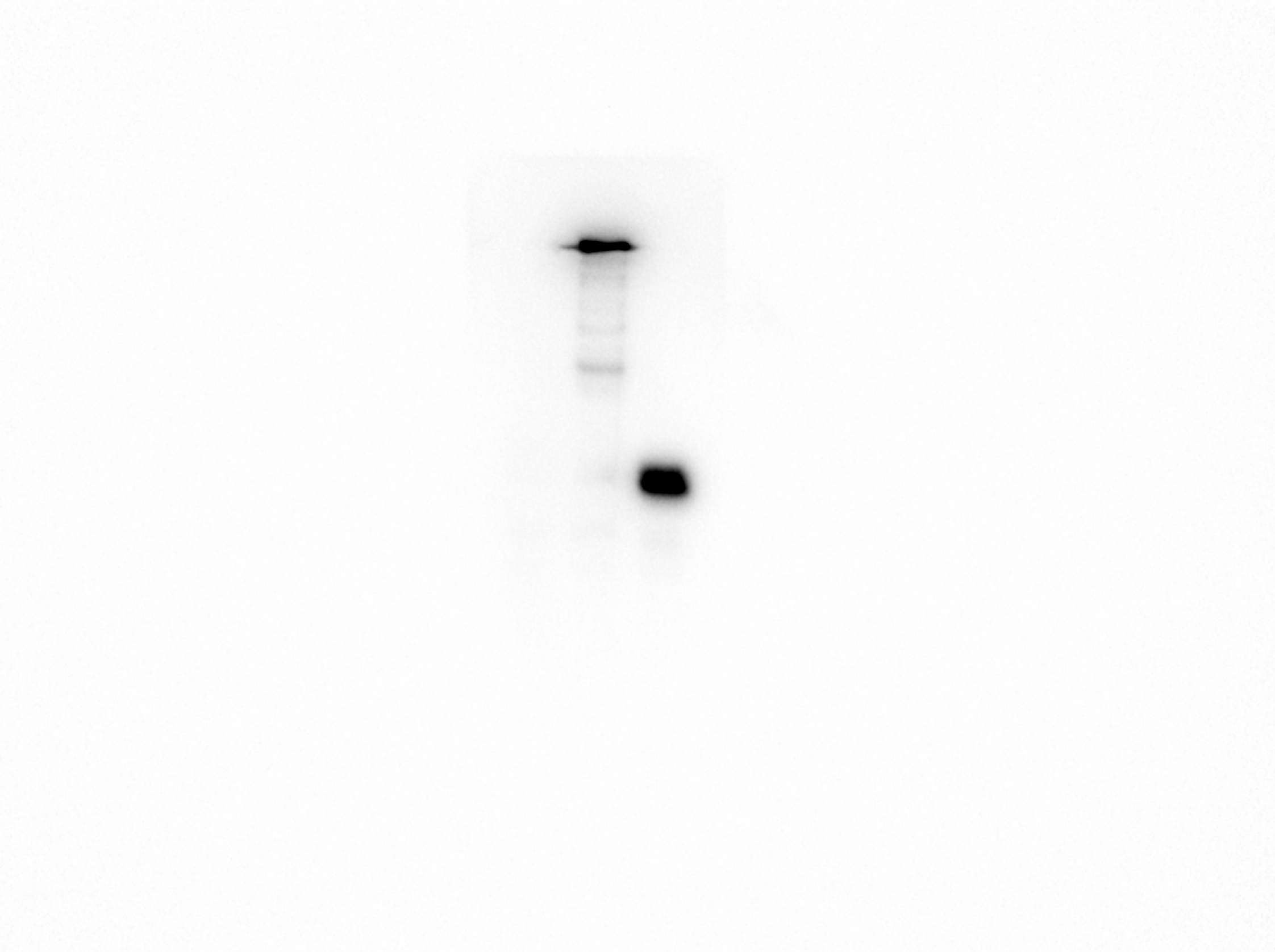

Supplement: Figure 2—source data 1. [file elife-85728-fig2-data1.zip › Figure 2-source data 1/Figure 2c CO-IP ISW1-CHD1 HA input raw data.tif]

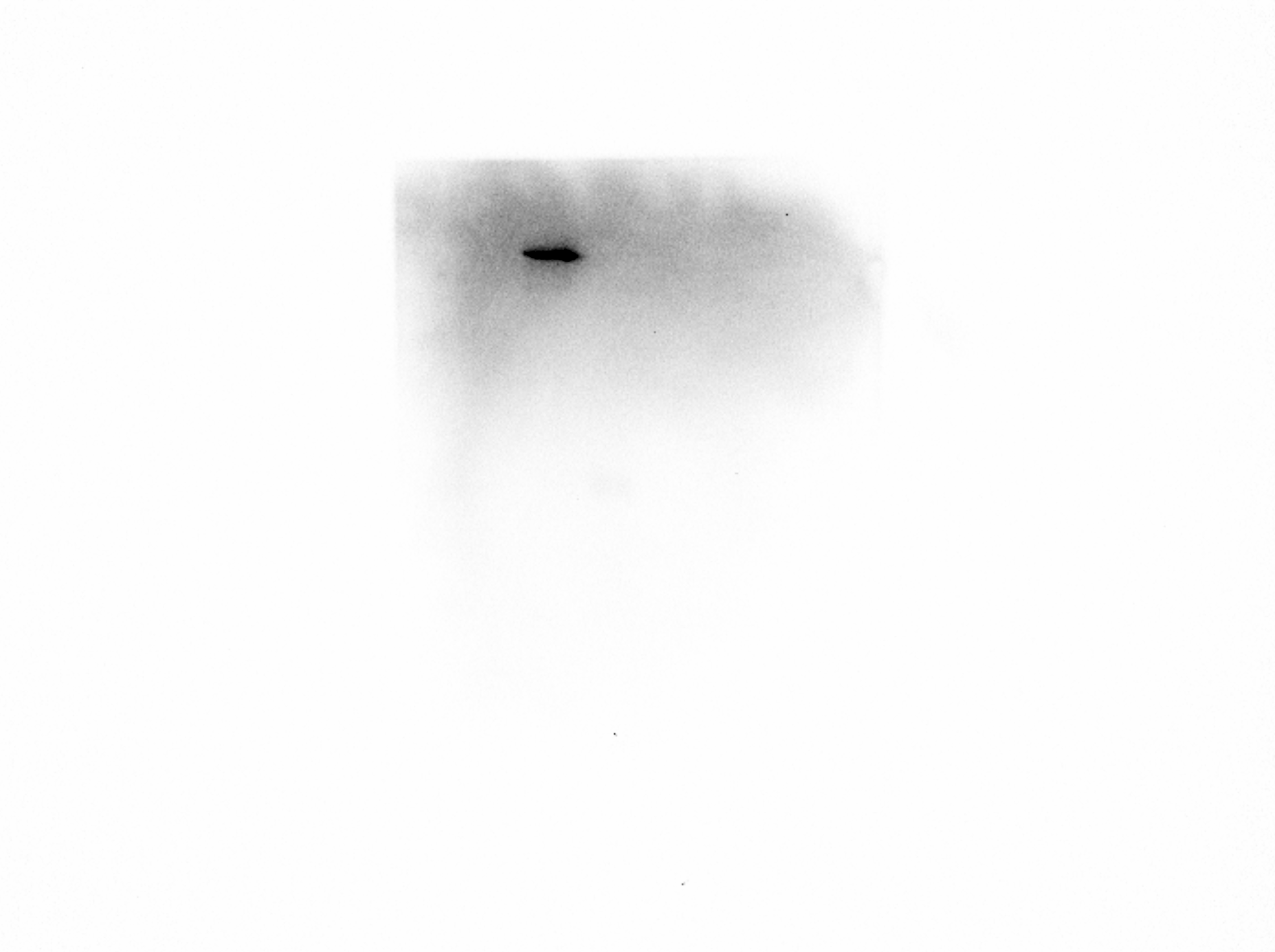

Supplement: Figure 2—source data 1. [file elife-85728-fig2-data1.zip › Figure 2-source data 1/Figure 2c CO-IP ISW1-CHD1 HA IP raw data.tif]

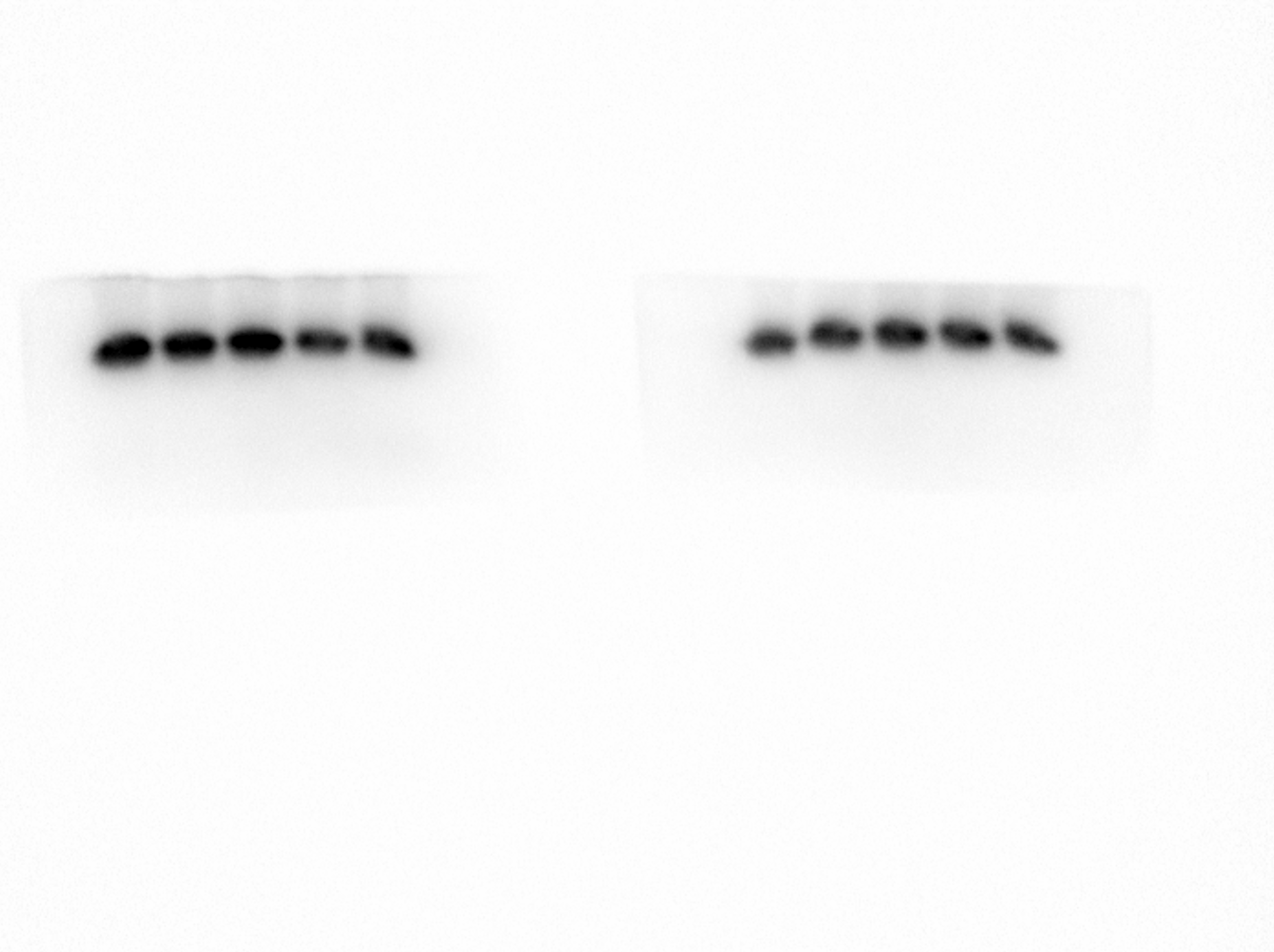

Supplement: Figure 3—figure supplement 1—source data 1. [file elife-85728-fig3-figsupp1-data1.zip › Figure 3-figure supplement 1-source data 1/Figure 3-figure supplement 1 histone raw data.tif]

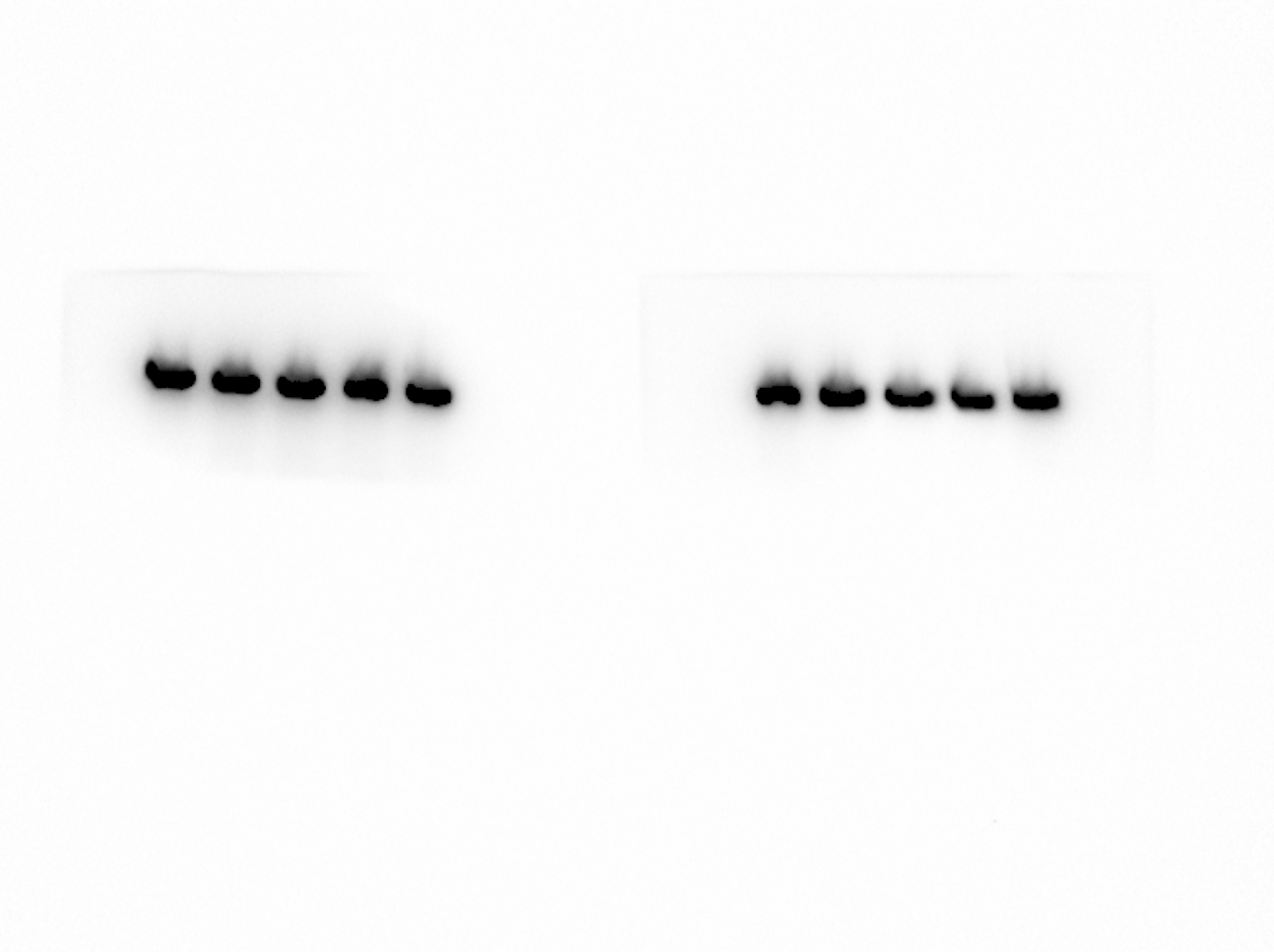

Supplement: Figure 3—figure supplement 1—source data 1. [file elife-85728-fig3-figsupp1-data1.zip › Figure 3-figure supplement 1-source data 1/Figure 3-figure supplement 1 isw1-flag raw data.tif]

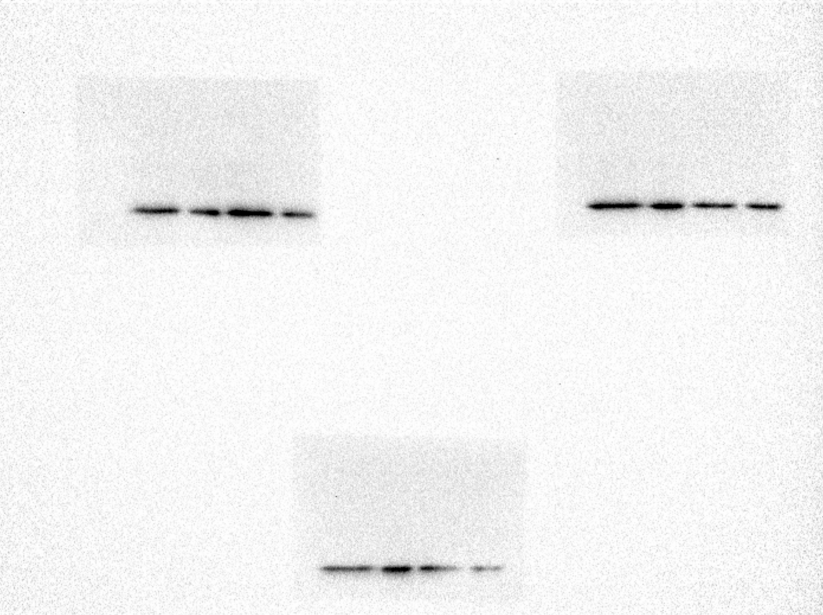

Supplement: Figure 4—source data 1. [file elife-85728-fig4-data1.zip › Figure 4-source data 1/Figure 4a histone raw data.tiff]

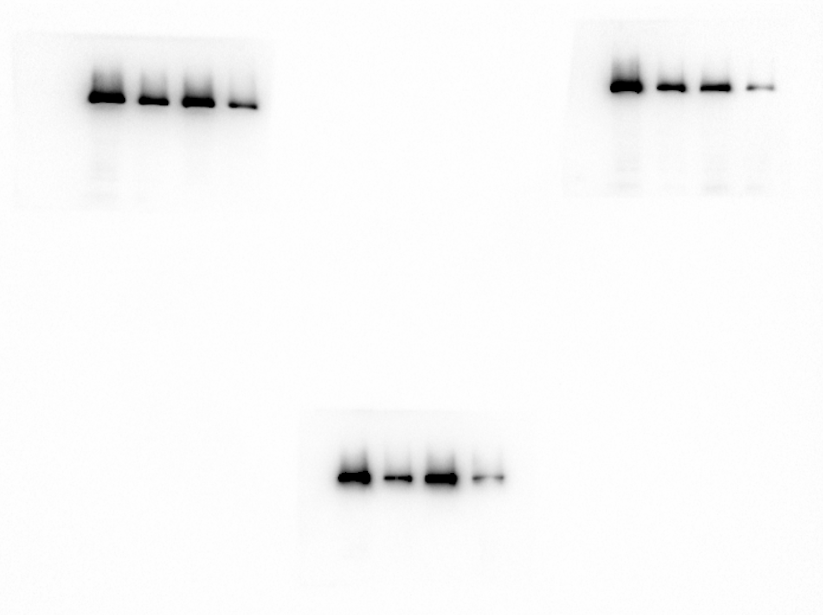

Supplement: Figure 4—source data 1. [file elife-85728-fig4-data1.zip › Figure 4-source data 1/Figure 4a isw1-flag raw data.tiff]

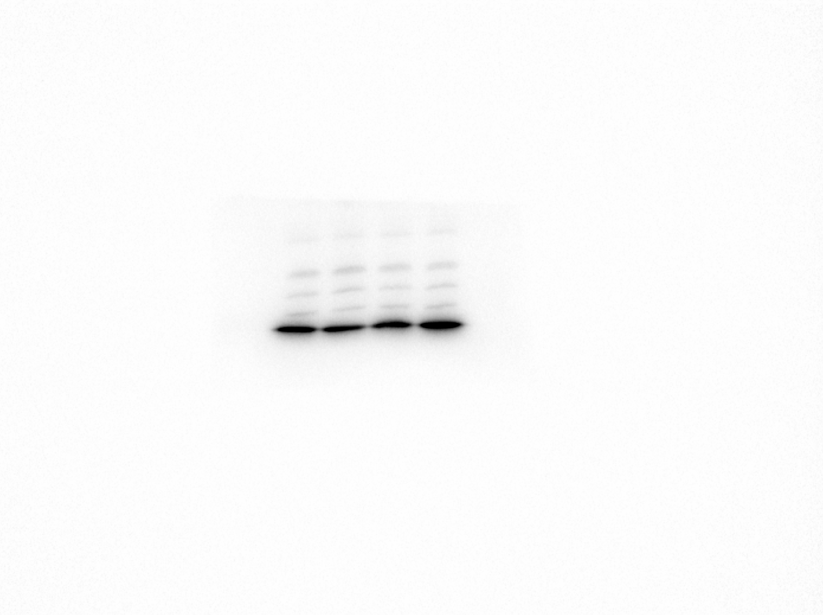

Supplement: Figure 4—source data 1. [file elife-85728-fig4-data1.zip › Figure 4-source data 1/Figure 4b histone raw data.tiff]

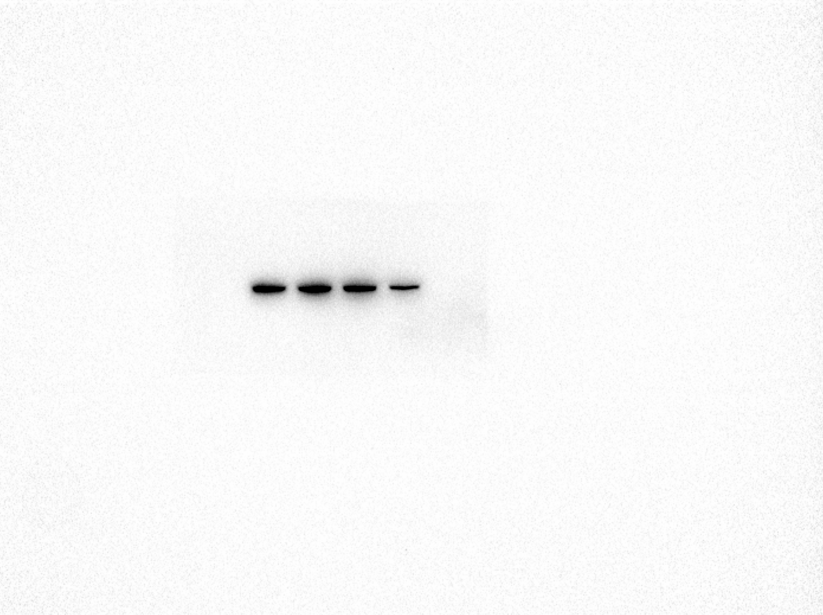

Supplement: Figure 4—source data 1. [file elife-85728-fig4-data1.zip › Figure 4-source data 1/Figure 4b isw1-flag raw data.tiff]

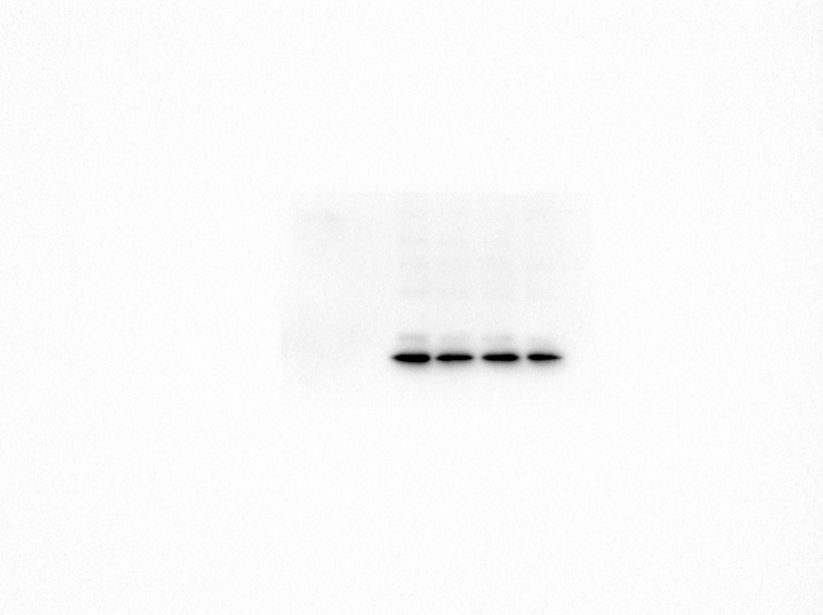

Supplement: Figure 4—source data 1. [file elife-85728-fig4-data1.zip › Figure 4-source data 1/Figure 4c histone raw data.tiff]

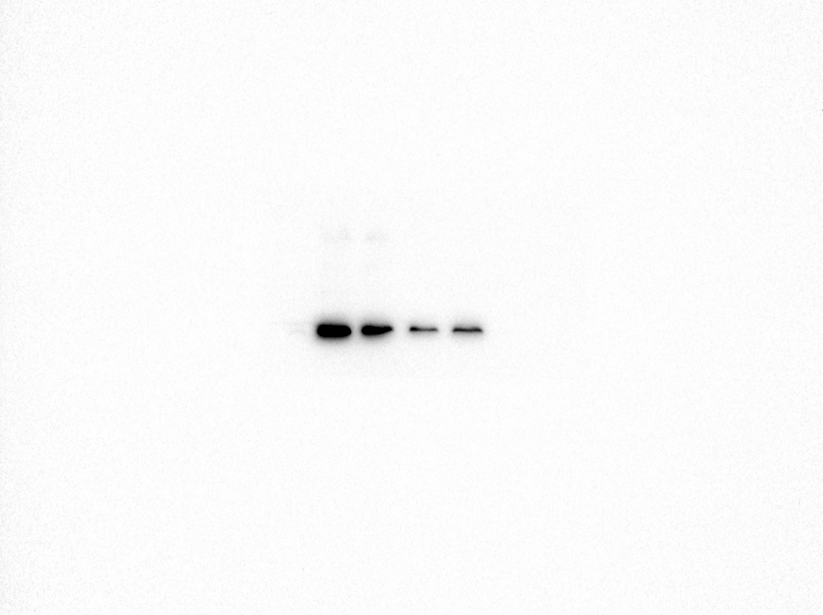

Supplement: Figure 4—source data 1. [file elife-85728-fig4-data1.zip › Figure 4-source data 1/Figure 4c isw1-flag raw data.tiff]

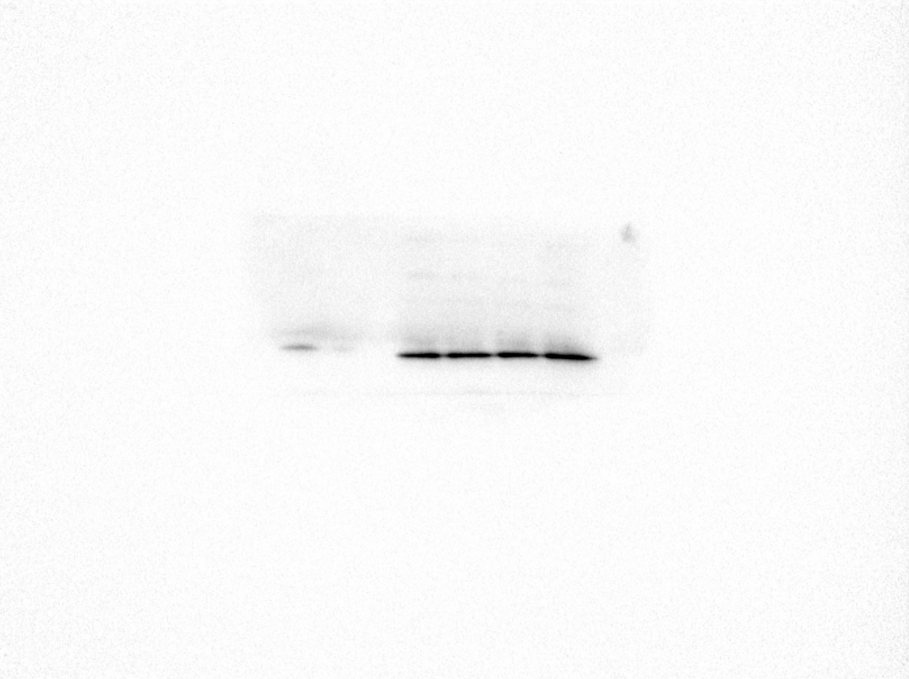

Supplement: Figure 4—source data 1. [file elife-85728-fig4-data1.zip › Figure 4-source data 1/Figure 4d histone raw data.tiff]

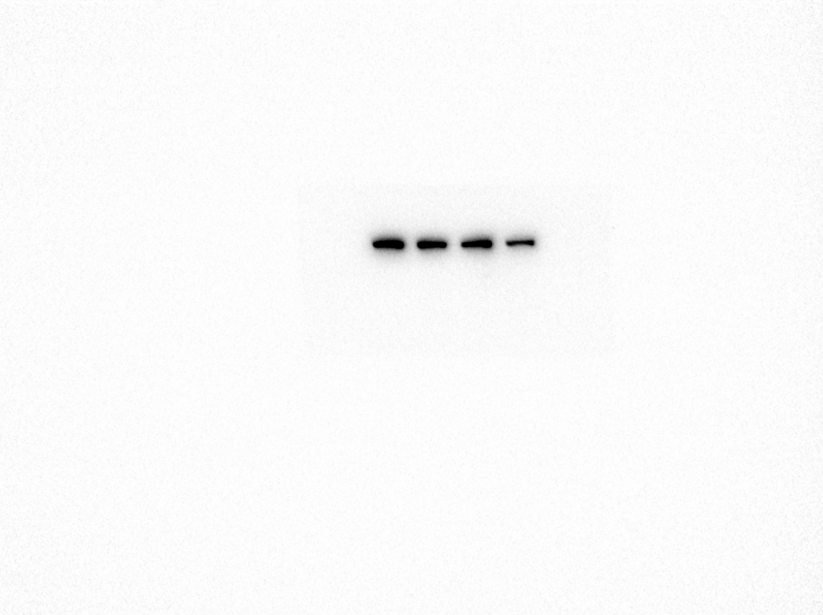

Supplement: Figure 4—source data 1. [file elife-85728-fig4-data1.zip › Figure 4-source data 1/Figure 4d isw1-flag raw data.tiff]

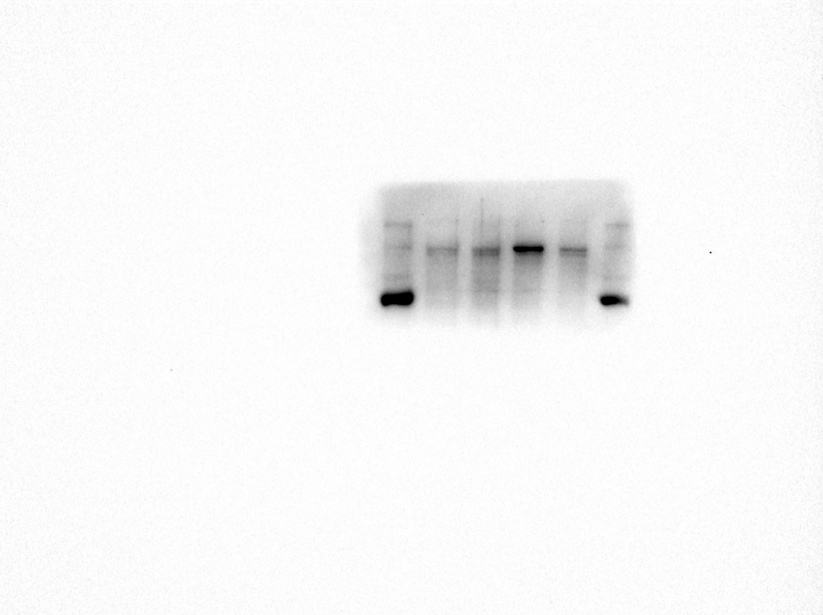

Supplement: Figure 5—source data 1. [file elife-85728-fig5-data1.zip › Figure 5-source data 1/Figure 5a isw1 Kac raw data.tiff]

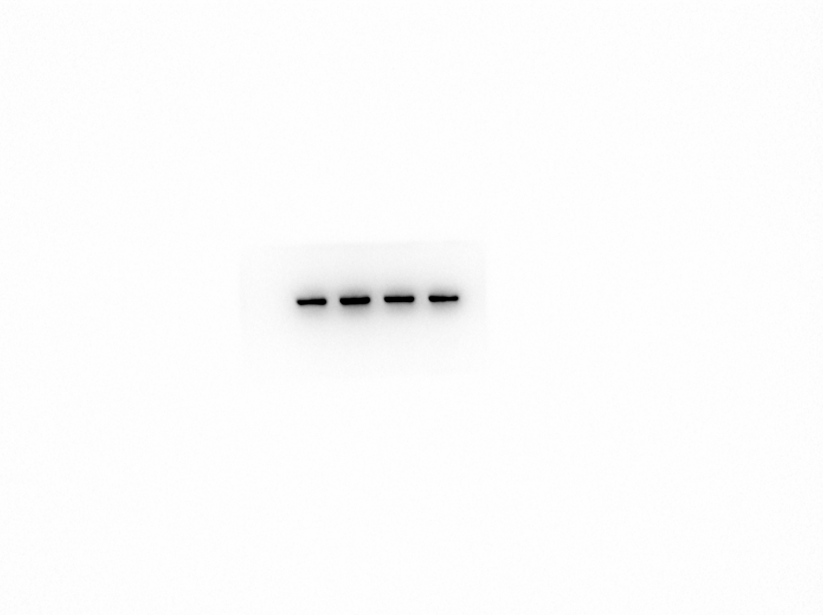

Supplement: Figure 5—source data 1. [file elife-85728-fig5-data1.zip › Figure 5-source data 1/Figure 5a isw1-flag raw data.tiff]

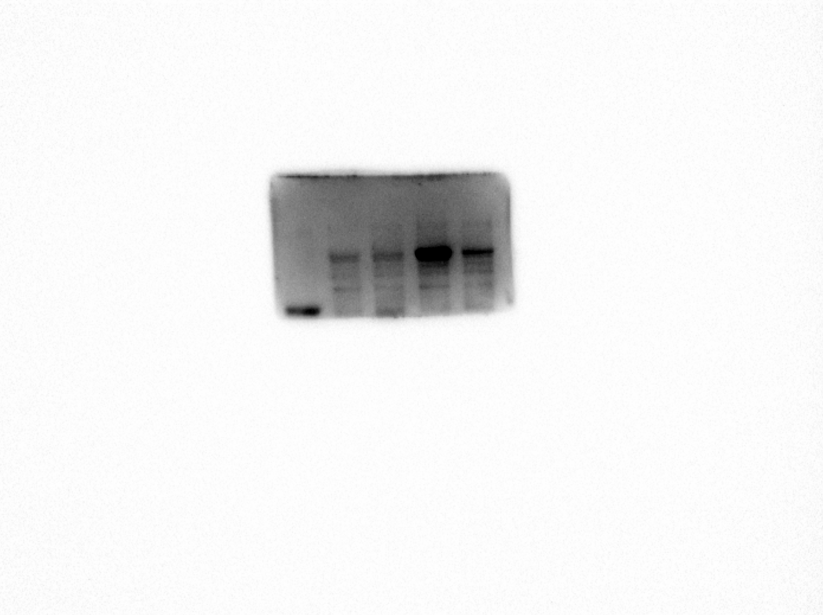

Supplement: Figure 5—source data 1. [file elife-85728-fig5-data1.zip › Figure 5-source data 1/Figure 5b isw1 Kac raw data.tiff]

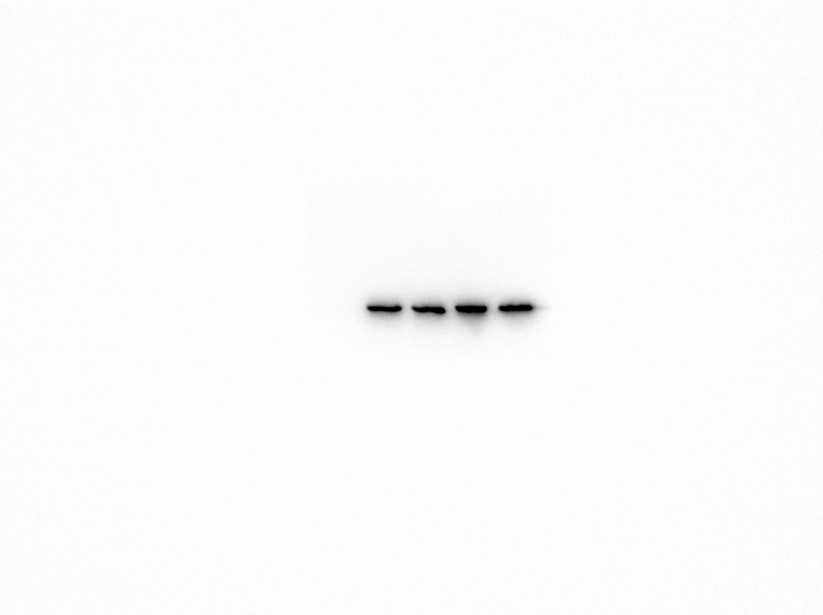

Supplement: Figure 5—source data 1. [file elife-85728-fig5-data1.zip › Figure 5-source data 1/Figure 5b isw1-flag raw data.tiff]

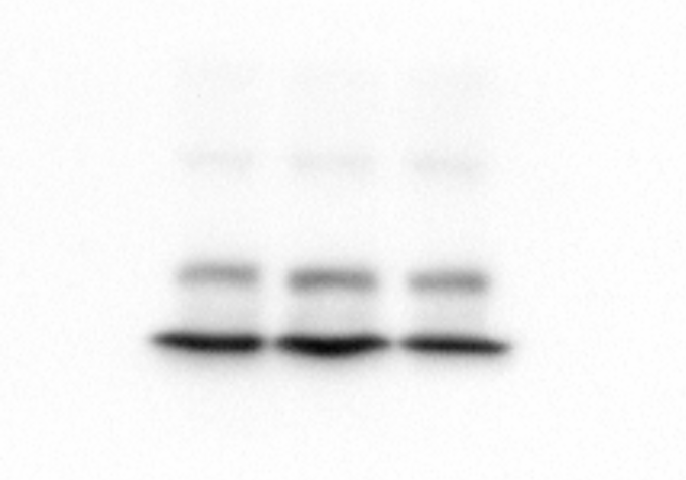

Supplement: Figure 5—source data 1. [file elife-85728-fig5-data1.zip › Figure 5-source data 1/Figure 5e histone raw data.tiff]

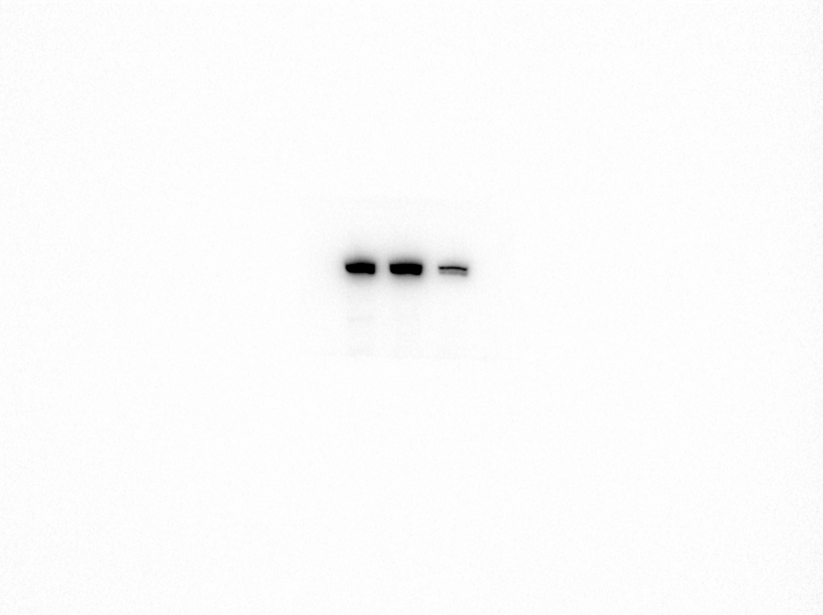

Supplement: Figure 5—source data 1. [file elife-85728-fig5-data1.zip › Figure 5-source data 1/Figure 5e isw1-flag raw data.tiff]

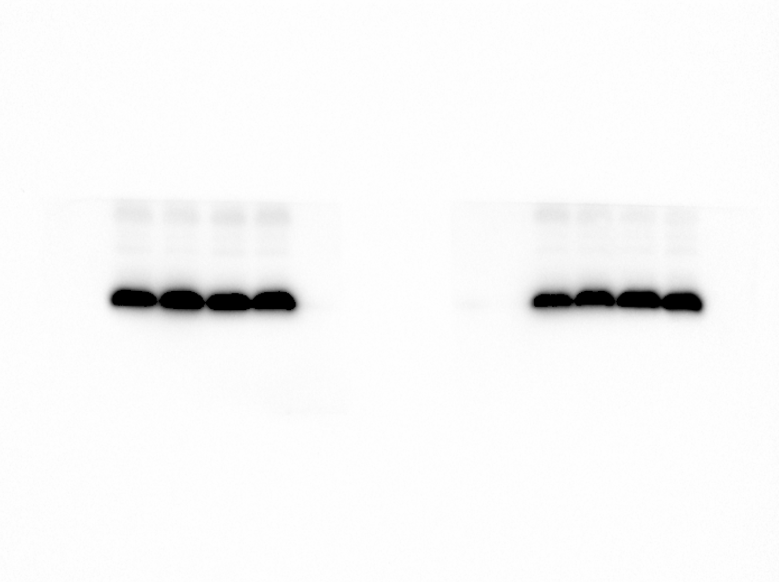

Supplement: Figure 5—source data 1. [file elife-85728-fig5-data1.zip › Figure 5-source data 1/Figure 5f histone raw data.tiff]

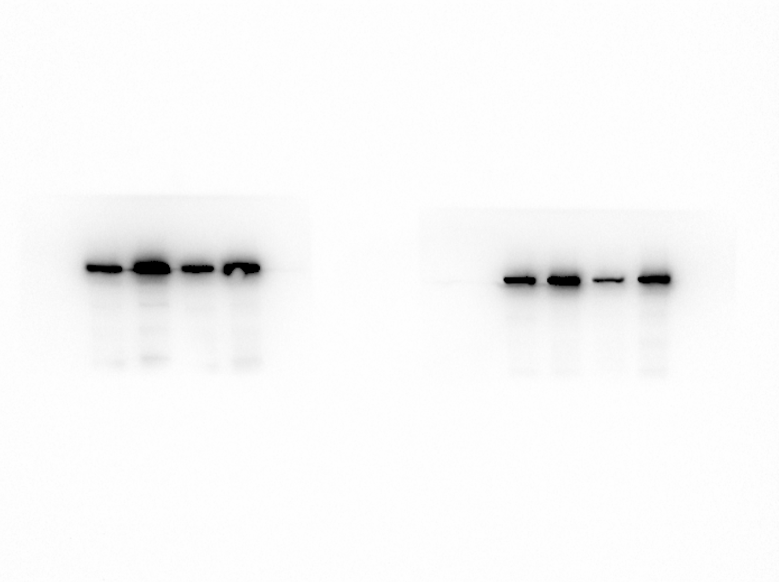

Supplement: Figure 5—source data 1. [file elife-85728-fig5-data1.zip › Figure 5-source data 1/Figure 5f isw1-flag raw data.tiff]

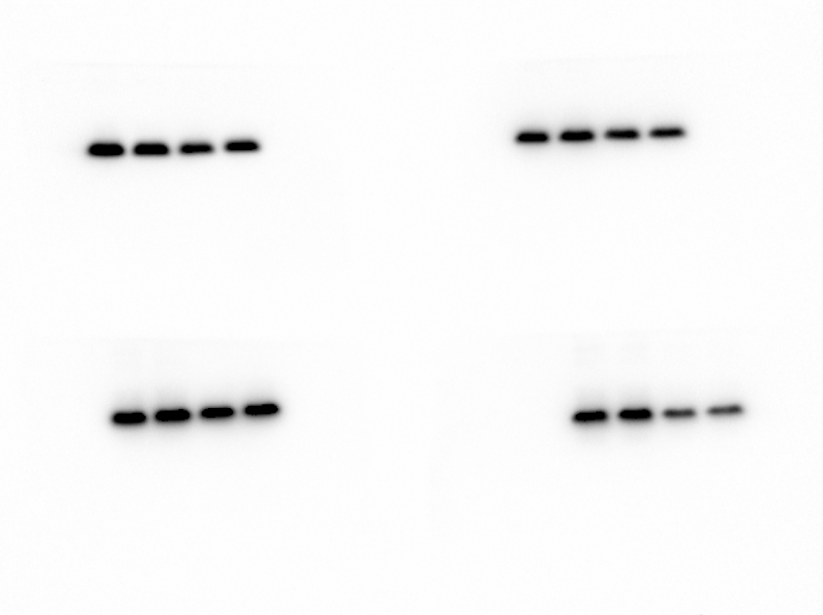

Supplement: Figure 5—source data 1. [file elife-85728-fig5-data1.zip › Figure 5-source data 1/Figure 5g histone raw data.tiff]

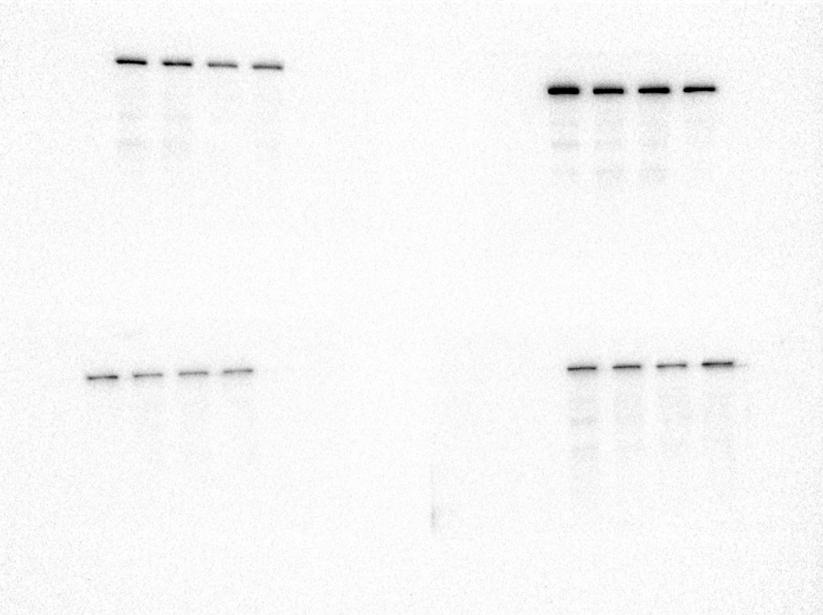

Supplement: Figure 5—source data 1. [file elife-85728-fig5-data1.zip › Figure 5-source data 1/Figure 5g isw1-flag raw data.tiff]

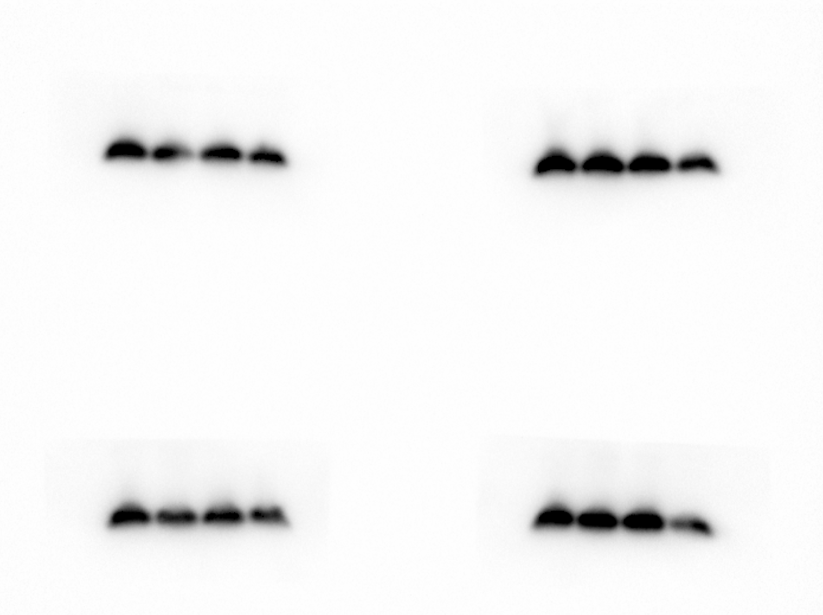

Supplement: Figure 5—source data 1. [file elife-85728-fig5-data1.zip › Figure 5-source data 1/Figure 5h histone raw data.tiff]

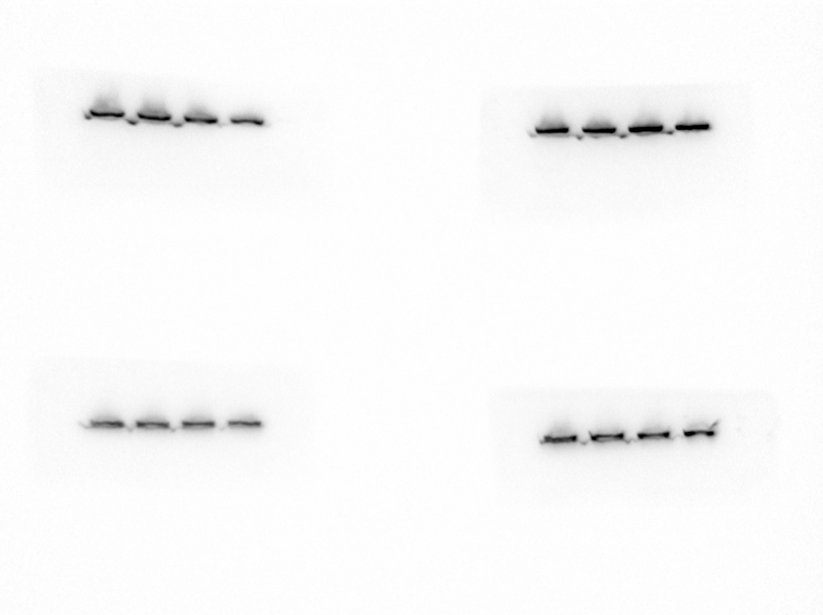

Supplement: Figure 5—source data 1. [file elife-85728-fig5-data1.zip › Figure 5-source data 1/Figure 5h isw1-flag raw data.tiff]

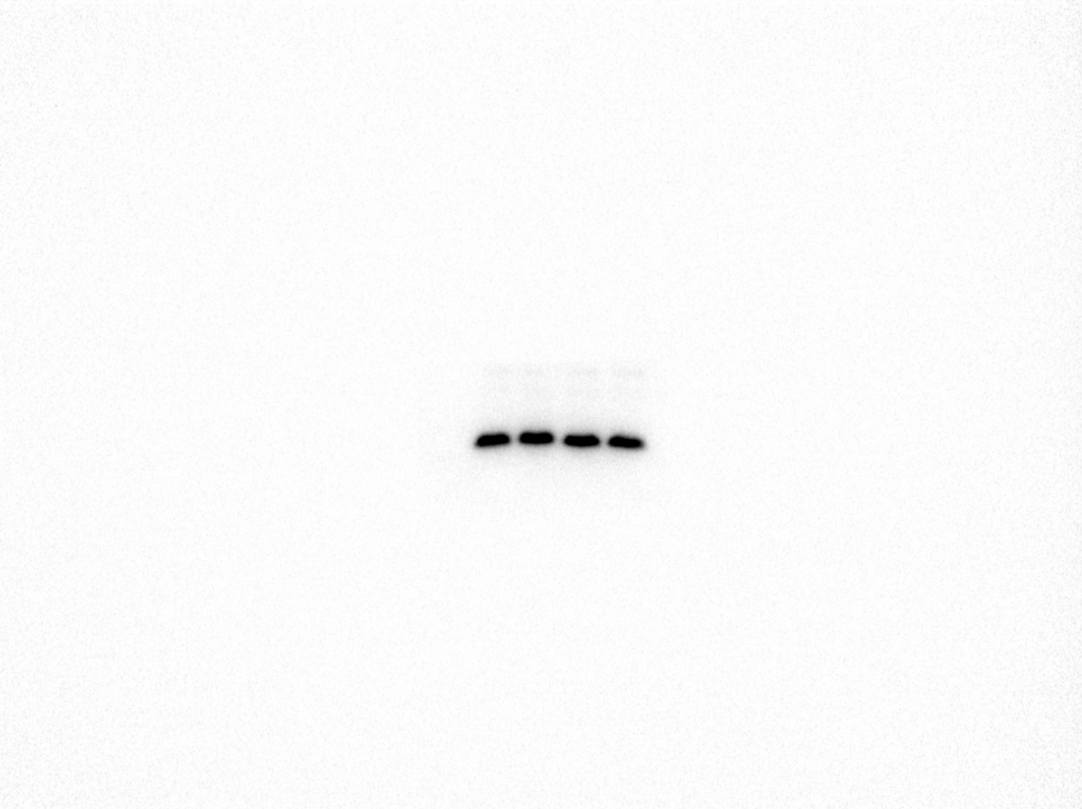

Supplement: Figure 5—source data 1. [file elife-85728-fig5-data1.zip › Figure 5-source data 1/Figure 5i histone raw data.tiff]

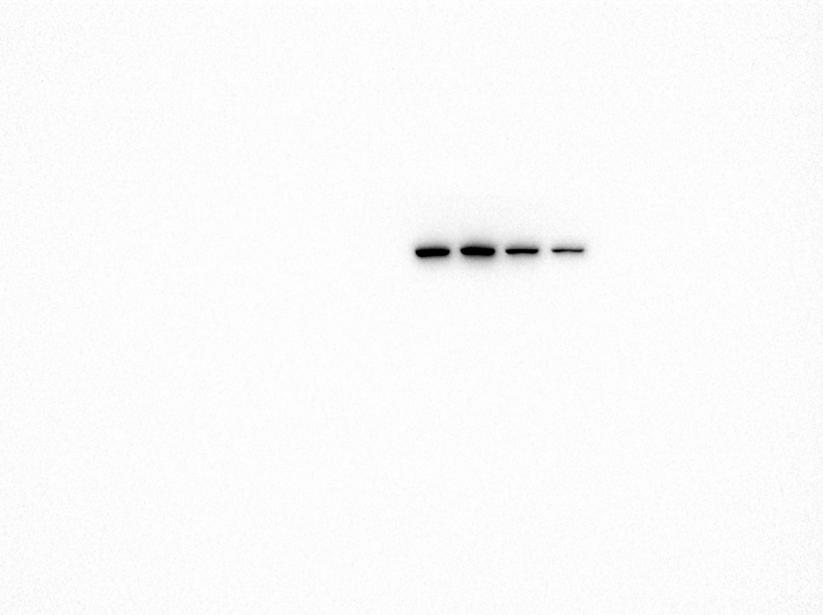

Supplement: Figure 5—source data 1. [file elife-85728-fig5-data1.zip › Figure 5-source data 1/Figure 5i isw1-flag raw data.tiff]

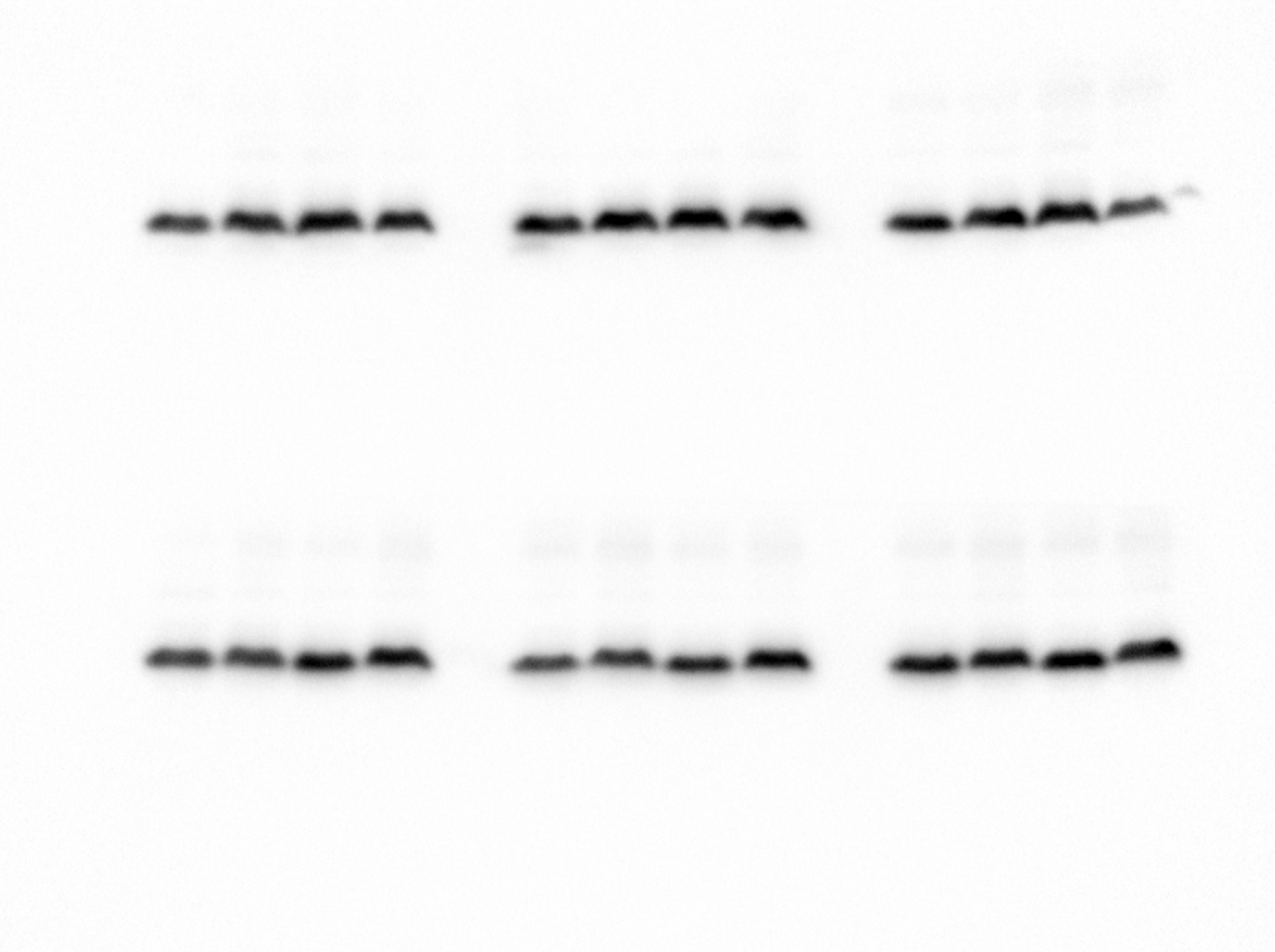

Supplement: Figure 5—figure supplement 1—source data 1. [file elife-85728-fig5-figsupp1-data1.zip › Figure 5-figure supplement 1-source data 1/Figure 5-figure supplement 1e histone raw data.tif]

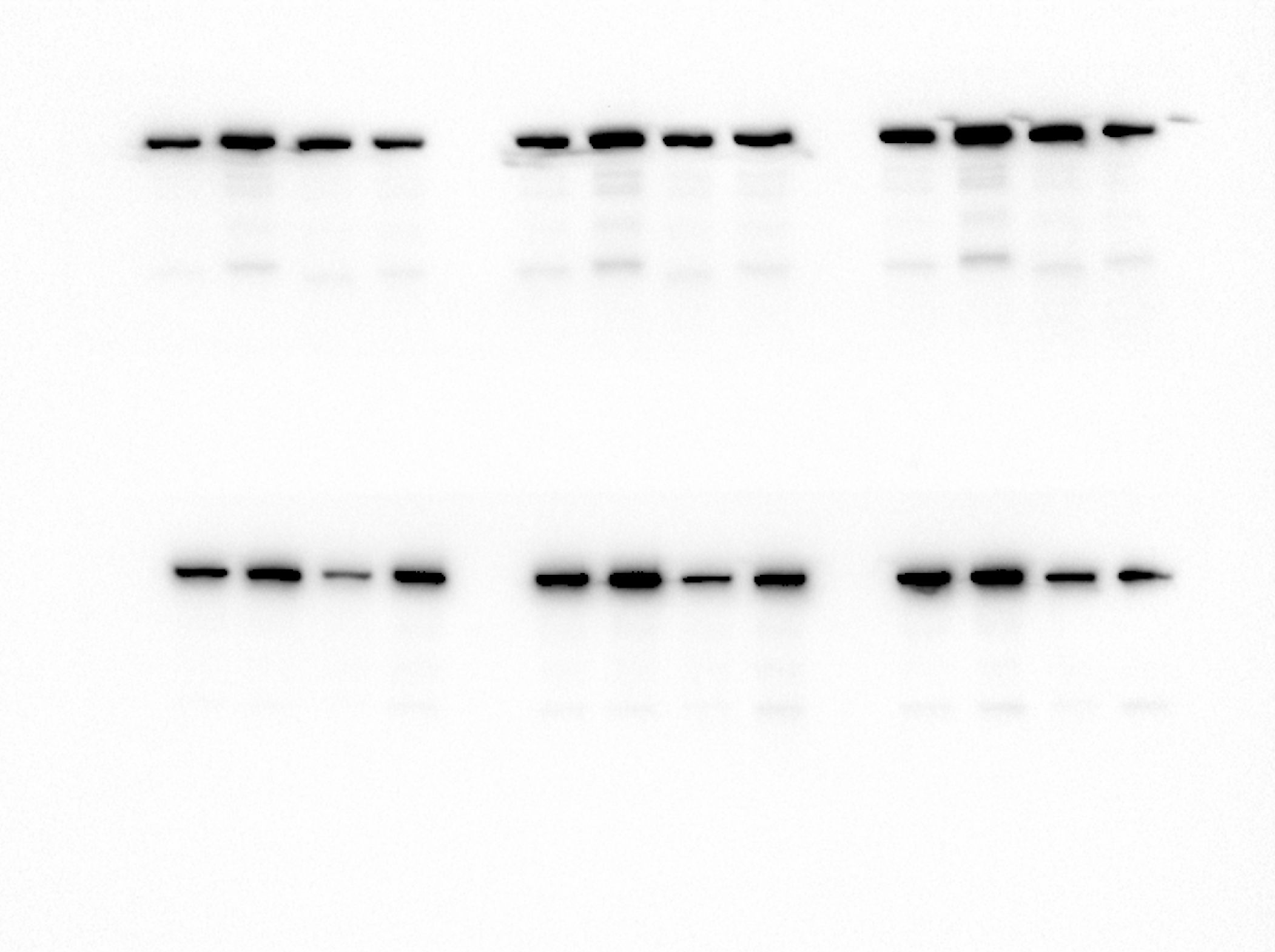

Supplement: Figure 5—figure supplement 1—source data 1. [file elife-85728-fig5-figsupp1-data1.zip › Figure 5-figure supplement 1-source data 1/Figure 5-figure supplement 1e isw1-flag raw data.tif]

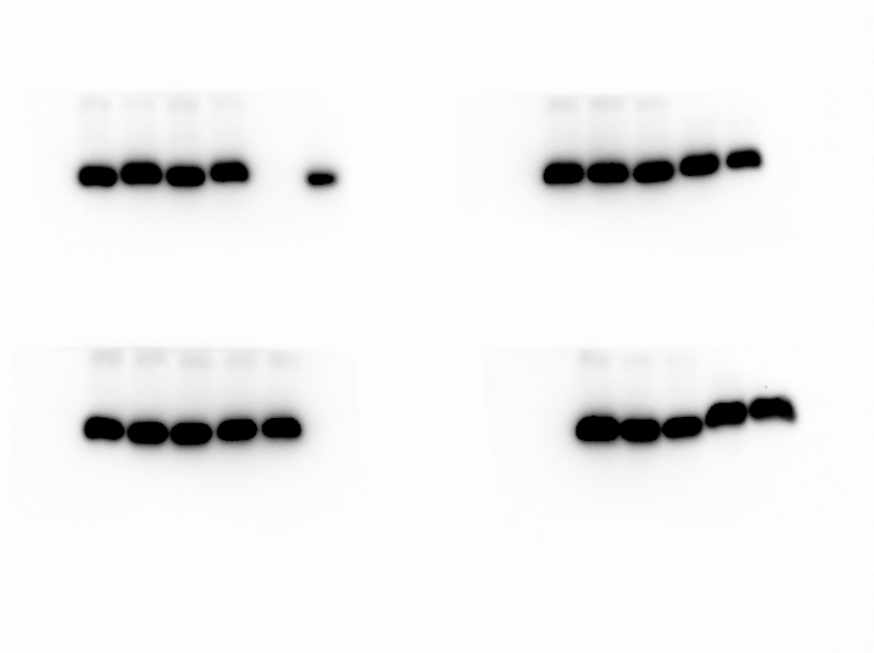

Supplement: Figure 6—source data 1. [file elife-85728-fig6-data1.zip › Figure 6-source data 1/Figure 6a histone raw datat .tiff]

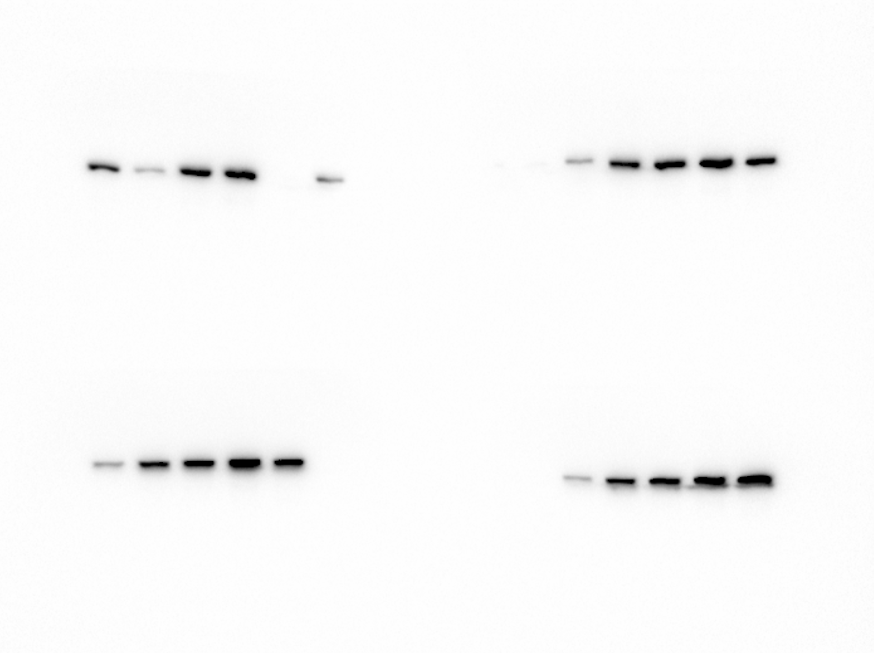

Supplement: Figure 6—source data 1. [file elife-85728-fig6-data1.zip › Figure 6-source data 1/Figure 6a isw1-flag raw data.tiff]

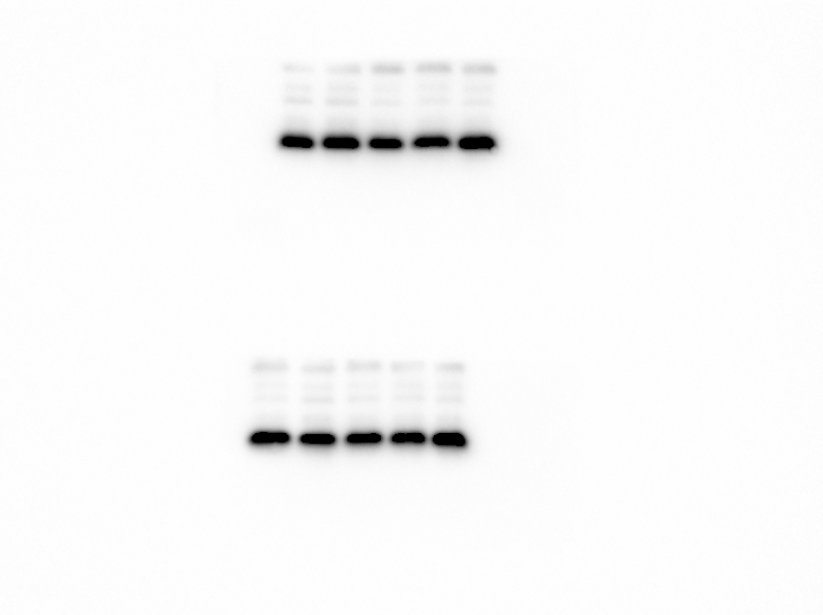

Supplement: Figure 6—source data 1. [file elife-85728-fig6-data1.zip › Figure 6-source data 1/Figure 6b histone raw data.tiff]

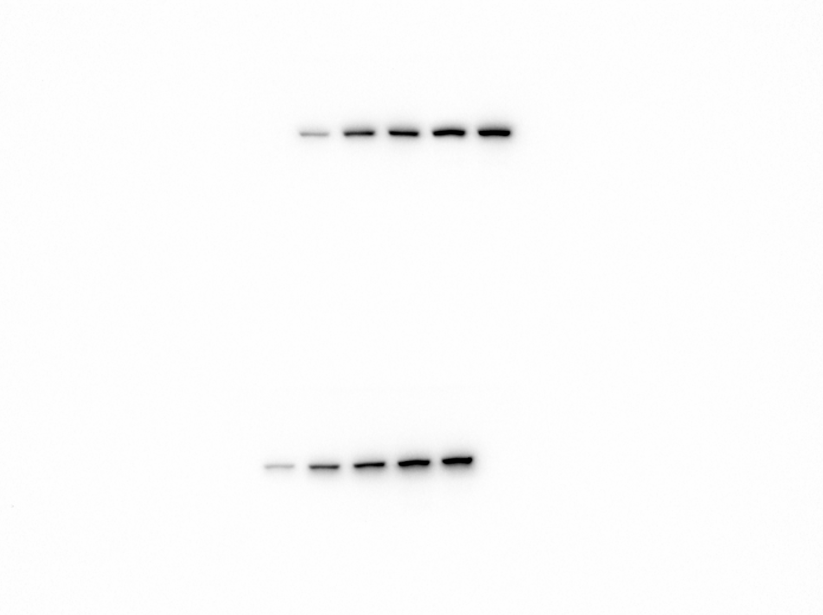

Supplement: Figure 6—source data 1. [file elife-85728-fig6-data1.zip › Figure 6-source data 1/Figure 6b isw1-flag raw data.tiff]

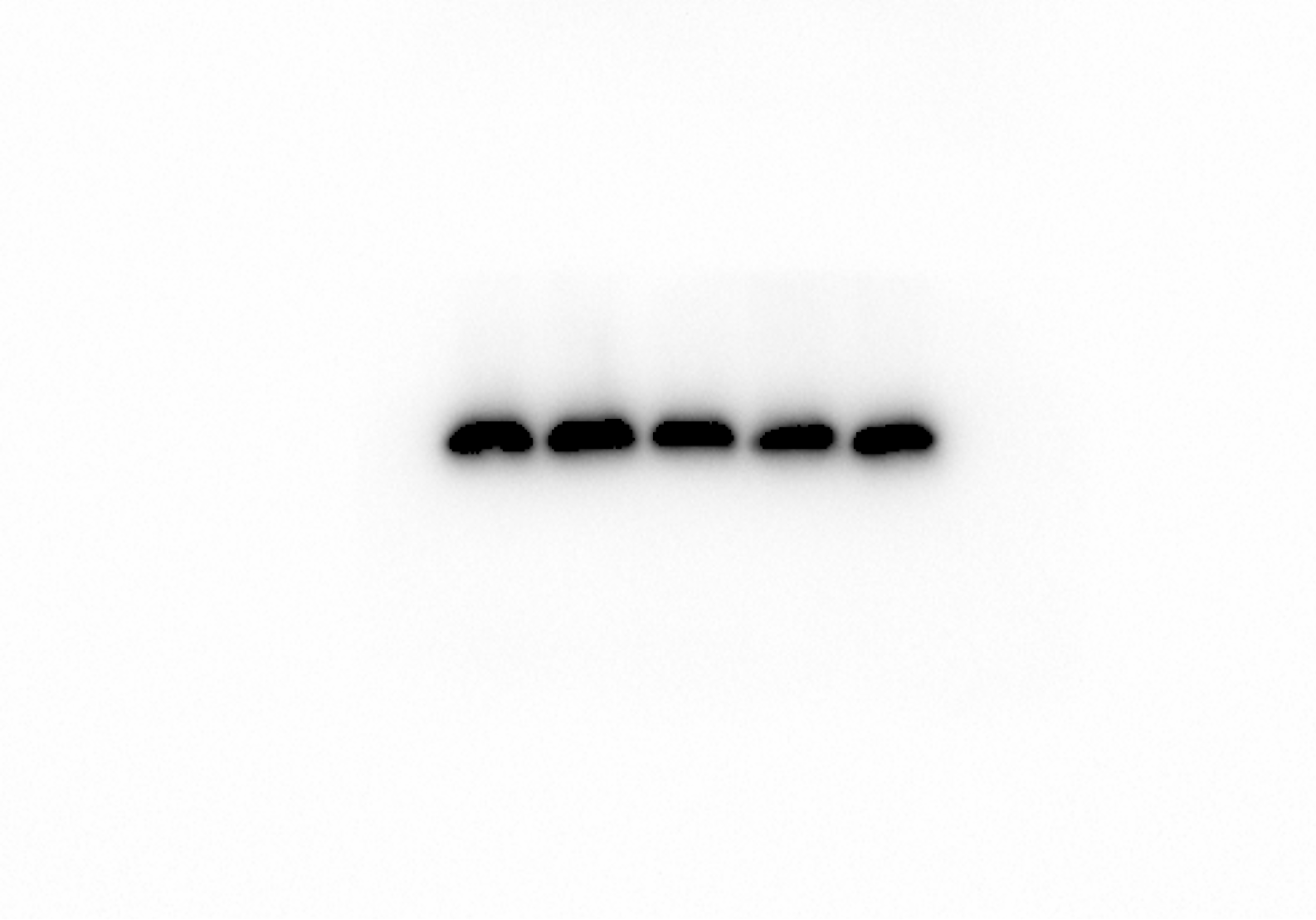

Supplement: Figure 6—source data 1. [file elife-85728-fig6-data1.zip › Figure 6-source data 1/Figure 6c histone raw data .tiff]

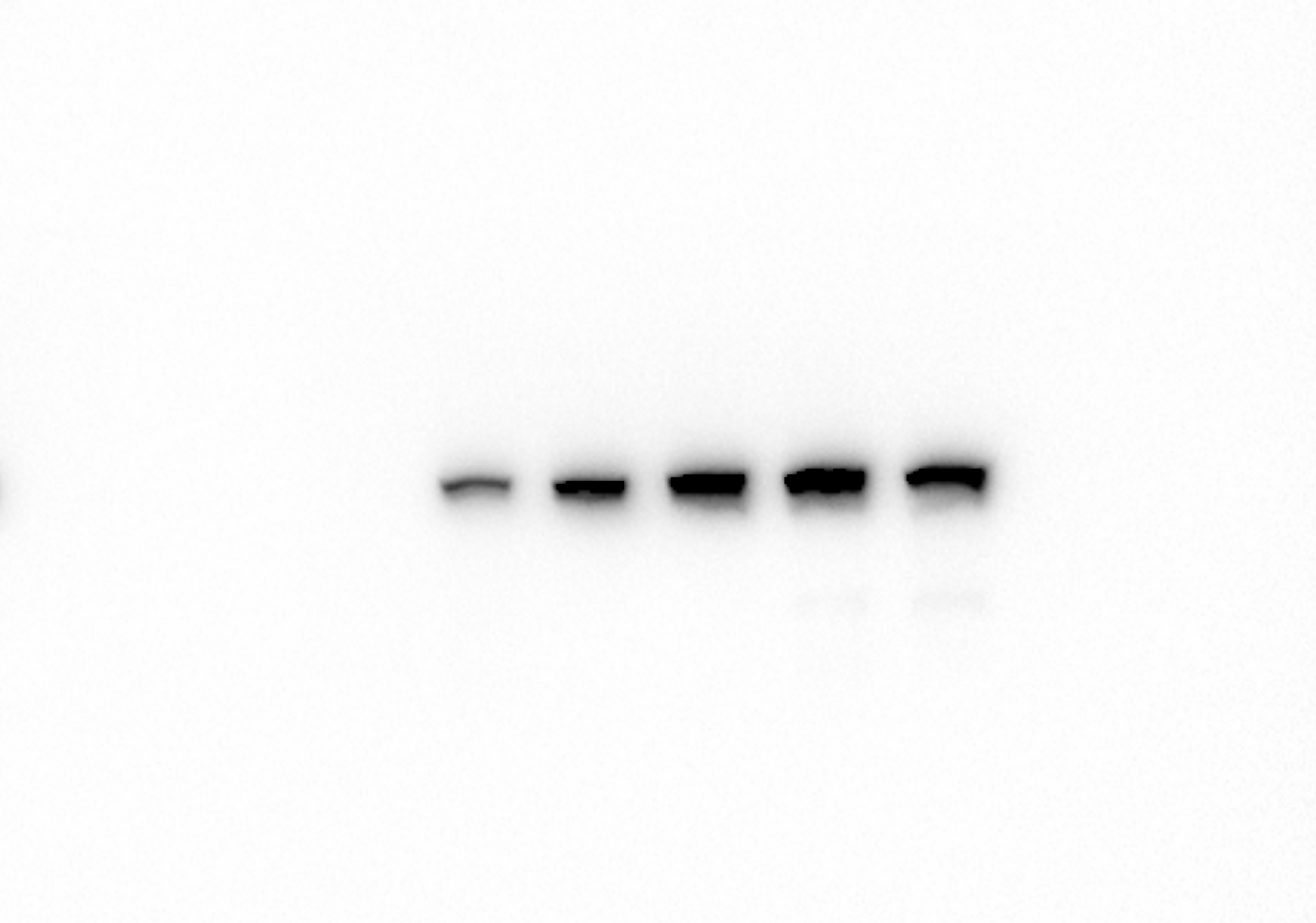

Supplement: Figure 6—source data 1. [file elife-85728-fig6-data1.zip › Figure 6-source data 1/Figure 6c isw1-flag raw data .tiff]

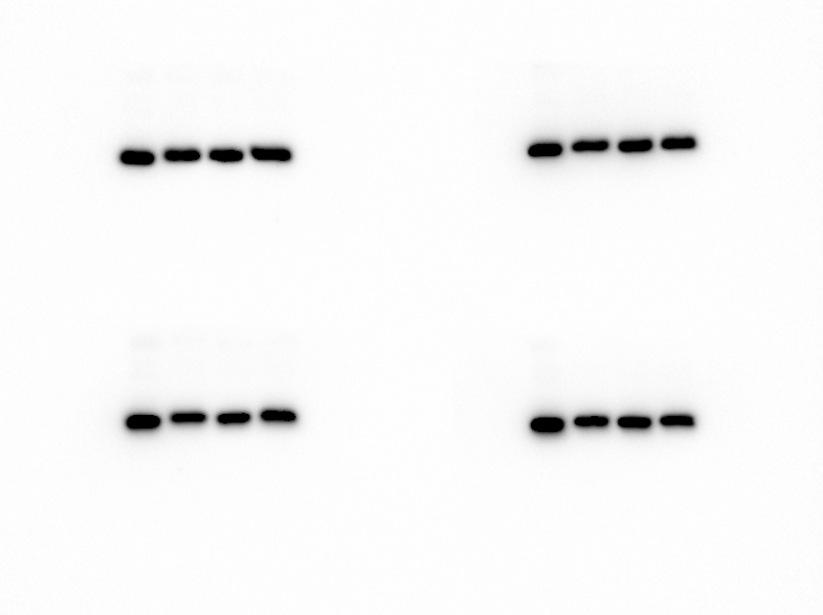

Supplement: Figure 6—source data 1. [file elife-85728-fig6-data1.zip › Figure 6-source data 1/Figure 6d histone raw data.tiff]

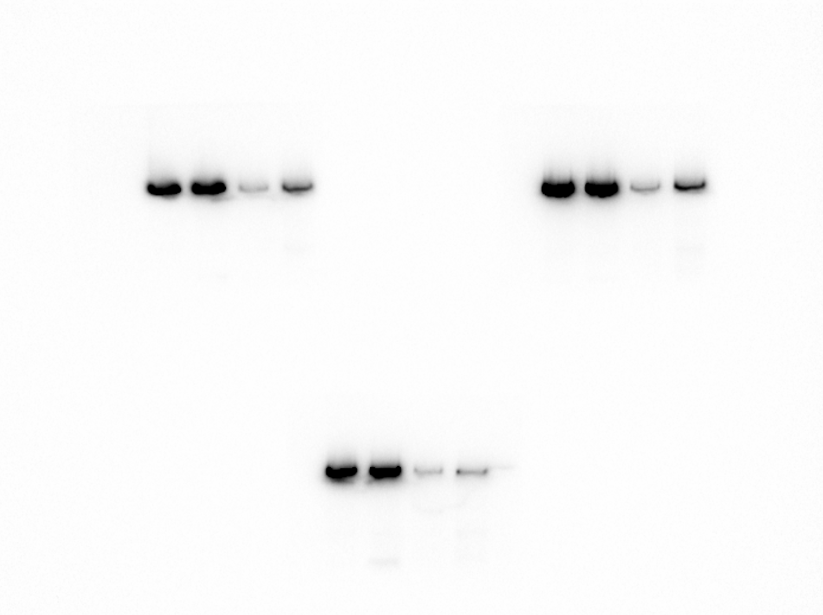

Supplement: Figure 6—source data 1. [file elife-85728-fig6-data1.zip › Figure 6-source data 1/Figure 6d isw1-flag raw data.tiff]

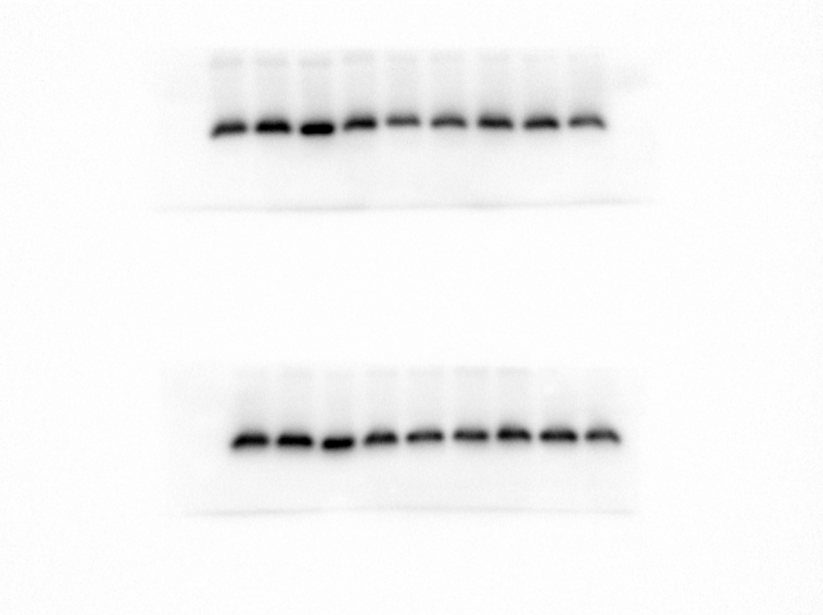

Supplement: Figure 6—source data 1. [file elife-85728-fig6-data1.zip › Figure 6-source data 1/Figure 6f histone raw data.tiff]

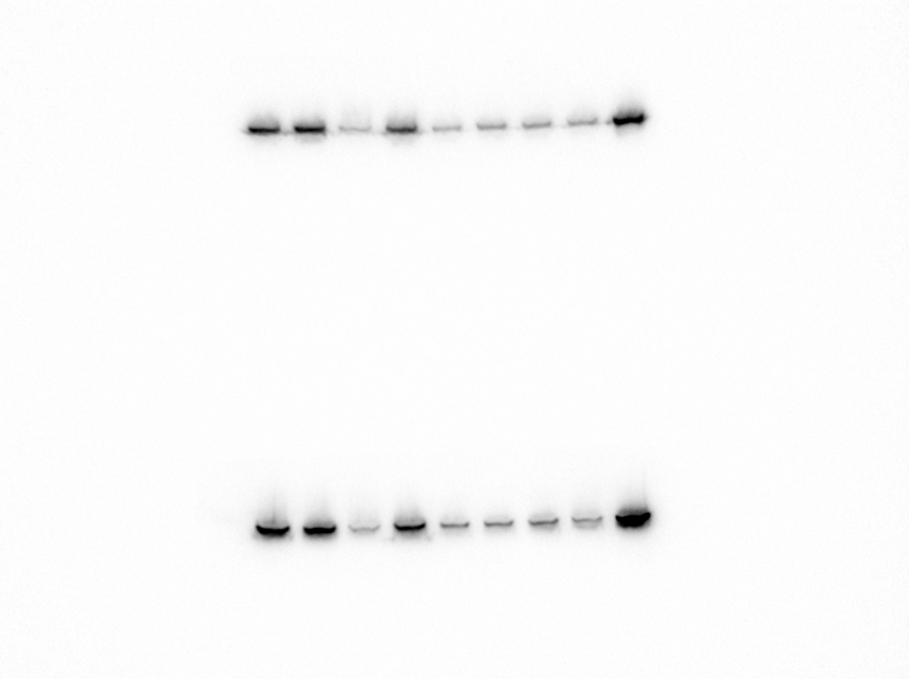

Supplement: Figure 6—source data 1. [file elife-85728-fig6-data1.zip › Figure 6-source data 1/Figure 6f isw1-flag raw data.tiff]

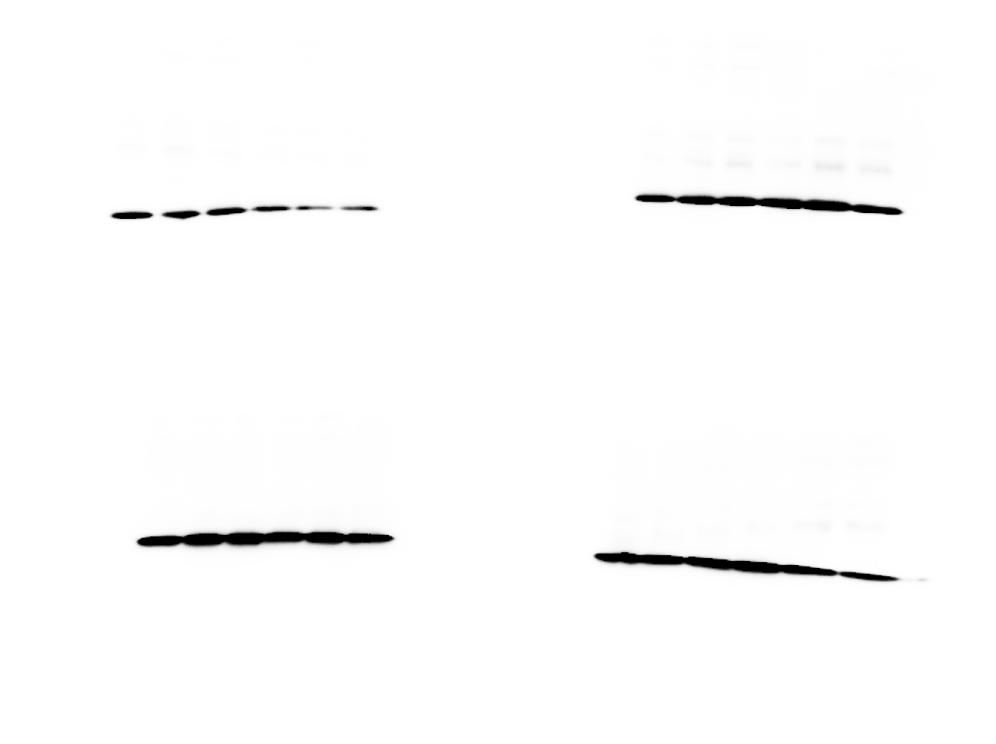

Supplement: Figure 7—source data 1. [file elife-85728-fig7-data1.zip › Figure 7-source data 1/Figure 7b histone raw data.tiff]

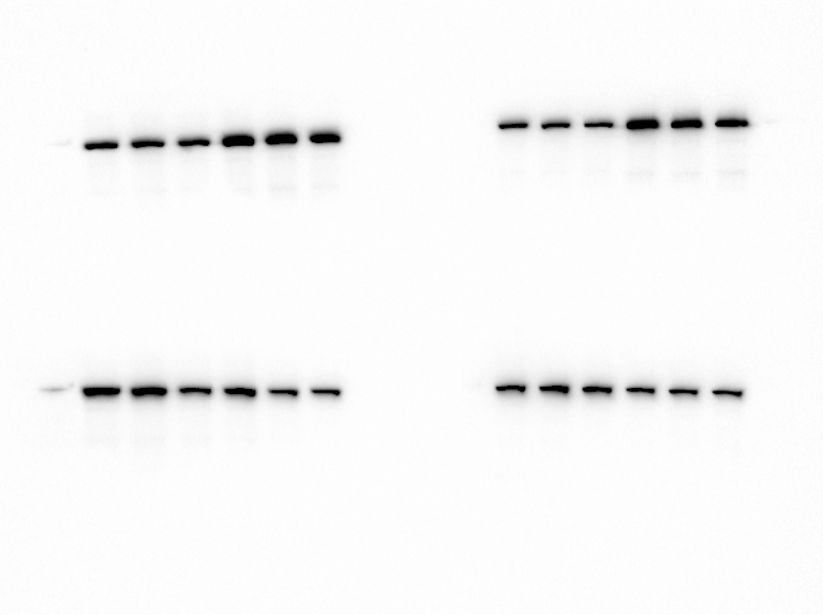

Supplement: Figure 7—source data 1. [file elife-85728-fig7-data1.zip › Figure 7-source data 1/Figure 7b isw1-flag raw data.tiff]

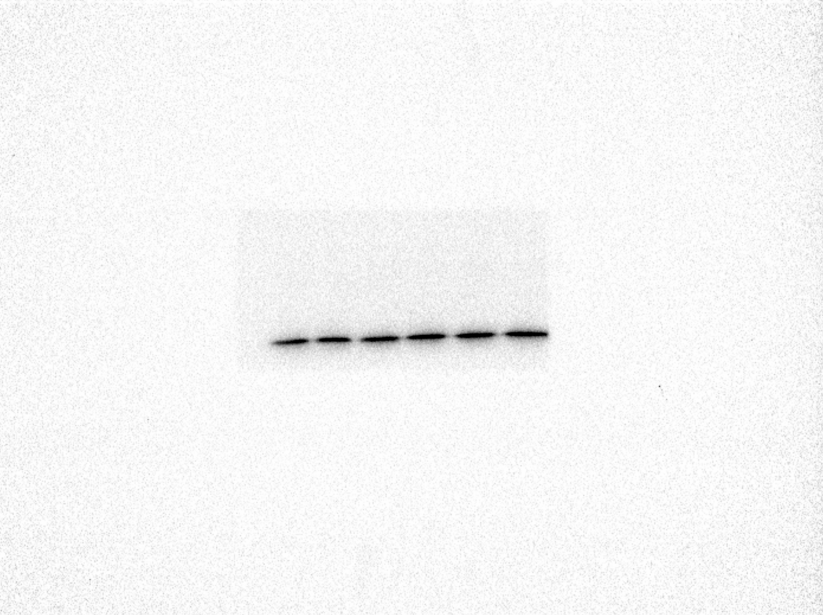

Supplement: Figure 7—source data 1. [file elife-85728-fig7-data1.zip › Figure 7-source data 1/Figure 7c histone raw data.tiff]

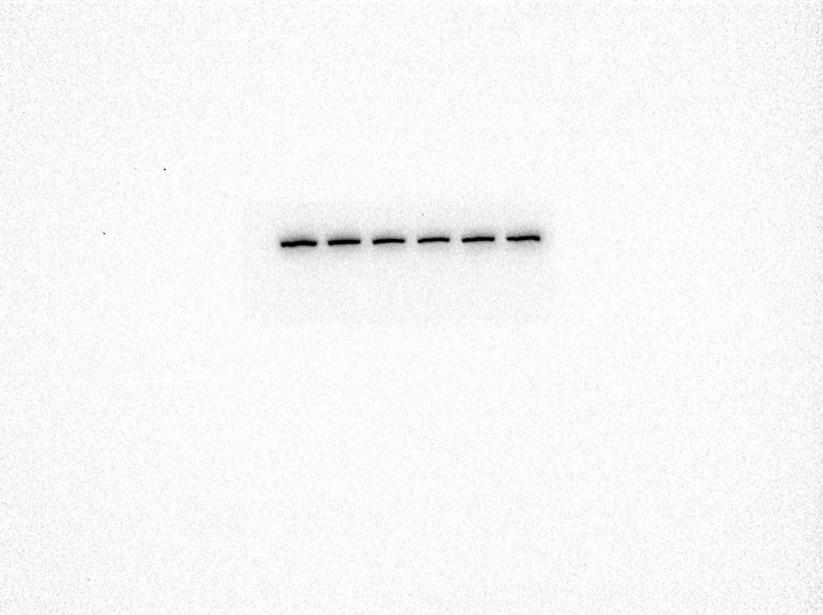

Supplement: Figure 7—source data 1. [file elife-85728-fig7-data1.zip › Figure 7-source data 1/Figure 7c isw1-flag raw data.tiff]

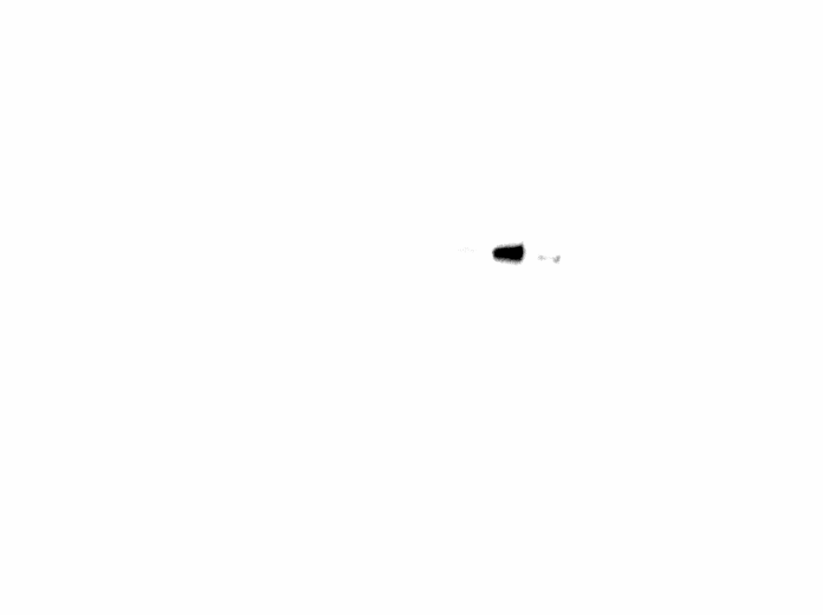

Supplement: Figure 7—source data 1. [file elife-85728-fig7-data1.zip › Figure 7-source data 1/Figure 7d cdc4-ha IP raw data.tiff]

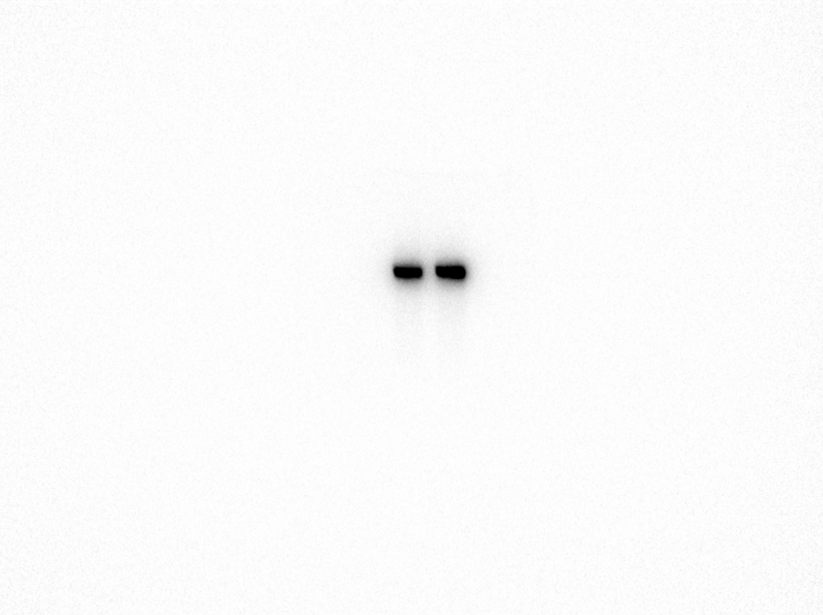

Supplement: Figure 7—source data 1. [file elife-85728-fig7-data1.zip › Figure 7-source data 1/Figure 7d cdc4-ha-input raw data.tiff]

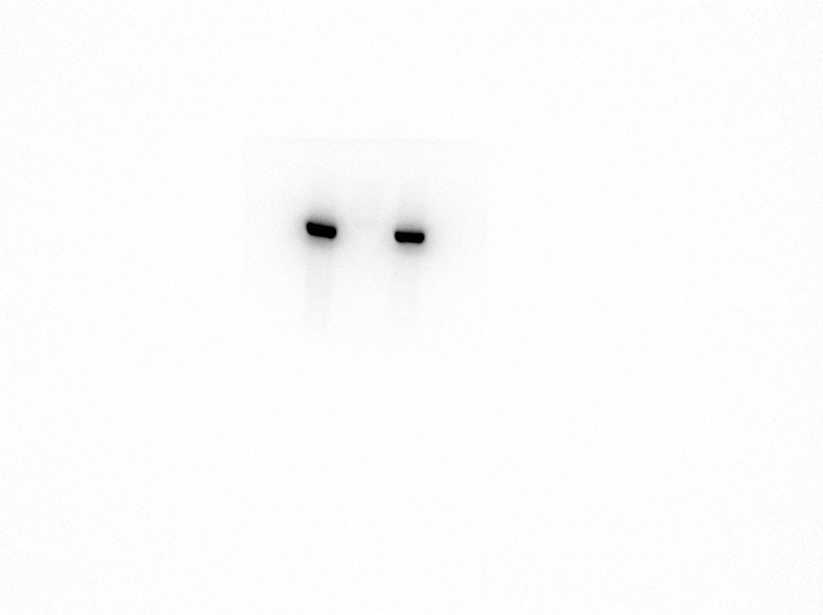

Supplement: Figure 7—source data 1. [file elife-85728-fig7-data1.zip › Figure 7-source data 1/Figure 7d isw1-flag input raw data.tiff]

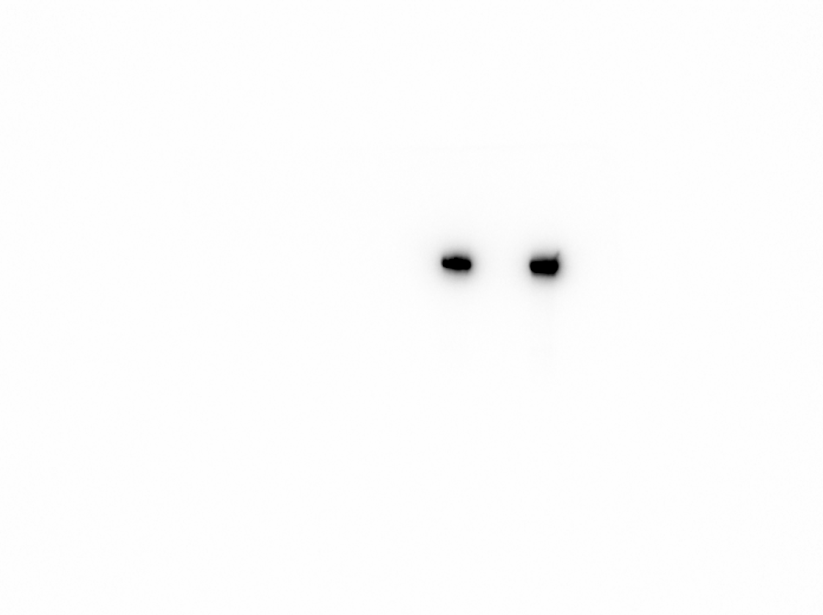

Supplement: Figure 7—source data 1. [file elife-85728-fig7-data1.zip › Figure 7-source data 1/Figure 7d isw1-flag IP raw data.tiff]

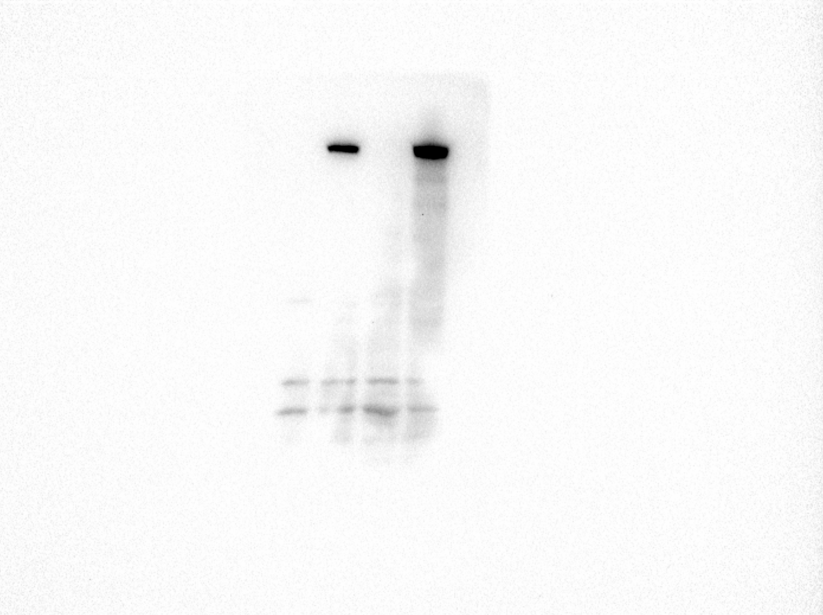

Supplement: Figure 7—source data 1. [file elife-85728-fig7-data1.zip › Figure 7-source data 1/Figure 7e cdc4-ha input raw data.tiff]

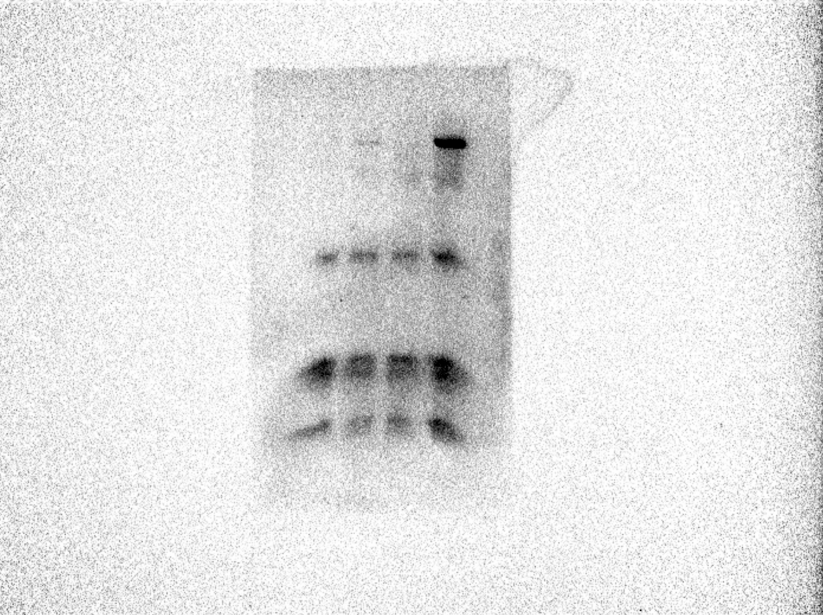

Supplement: Figure 7—source data 1. [file elife-85728-fig7-data1.zip › Figure 7-source data 1/Figure 7e cdc4-ha IP raw data.tiff]

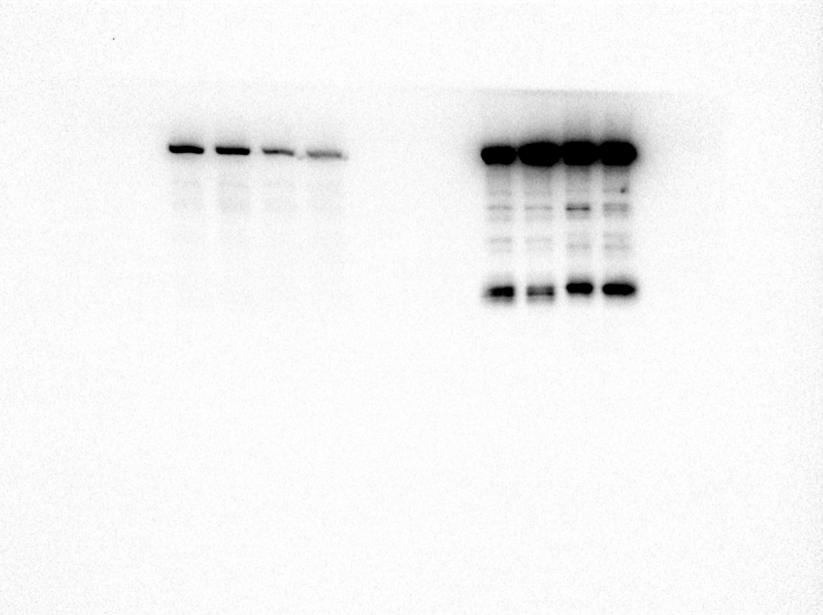

Supplement: Figure 7—source data 1. [file elife-85728-fig7-data1.zip › Figure 7-source data 1/Figure 7e isw1-flag input raw data.tiff]

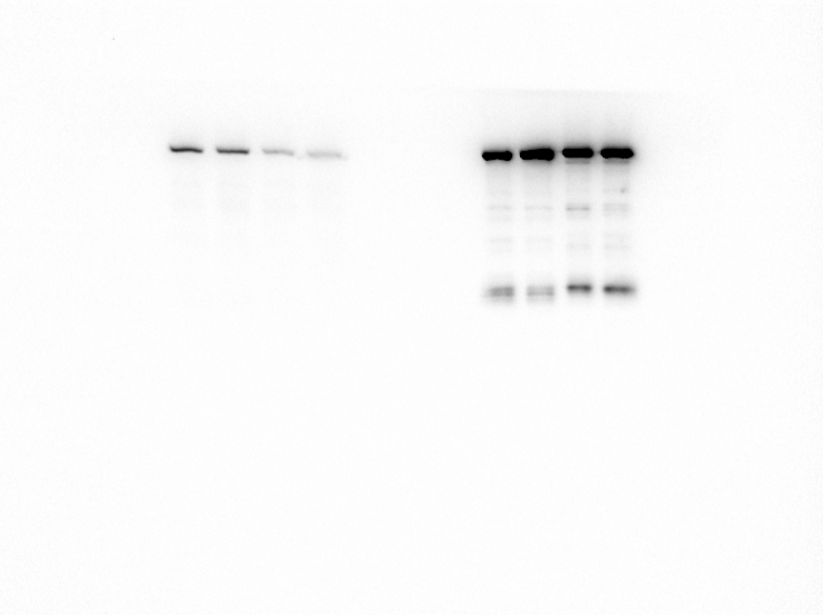

Supplement: Figure 7—source data 1. [file elife-85728-fig7-data1.zip › Figure 7-source data 1/Figure 7e isw1-flag IP raw data.tiff]

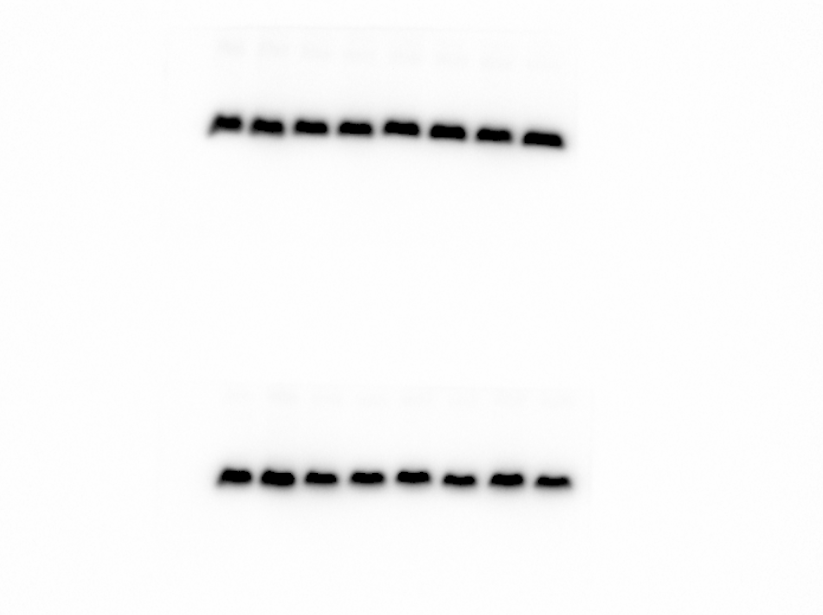

Supplement: Figure 8—source data 1. [file elife-85728-fig8-data1.zip › Figure 8-source data 1/Figure 8a CDLC15-120 histone raw data.tiff]

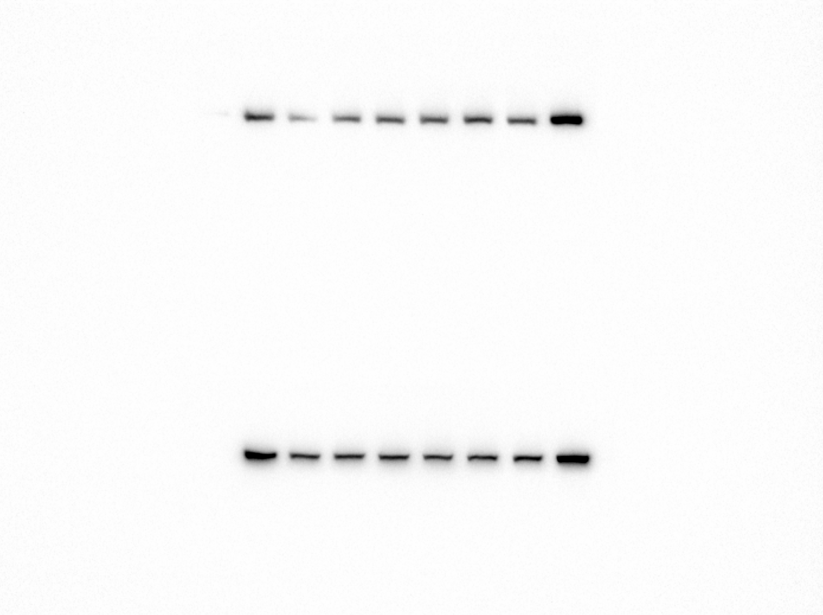

Supplement: Figure 8—source data 1. [file elife-85728-fig8-data1.zip › Figure 8-source data 1/Figure 8a CDLC15-120 iws1-flag raw data.tiff]

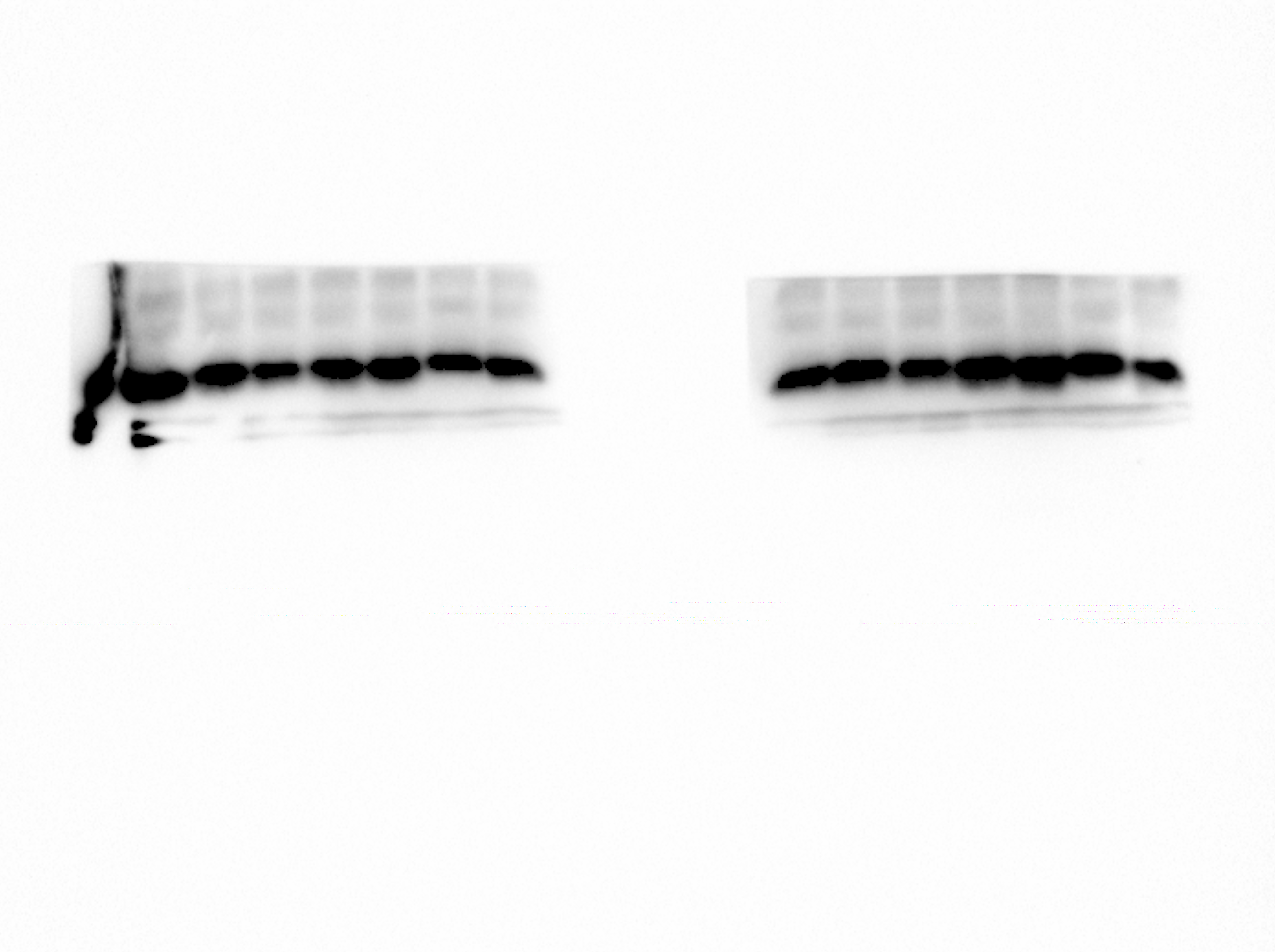

Supplement: Figure 8—source data 1. [file elife-85728-fig8-data1.zip › Figure 8-source data 1/Figure 8a CDLC4-141 histone raw data.tif]

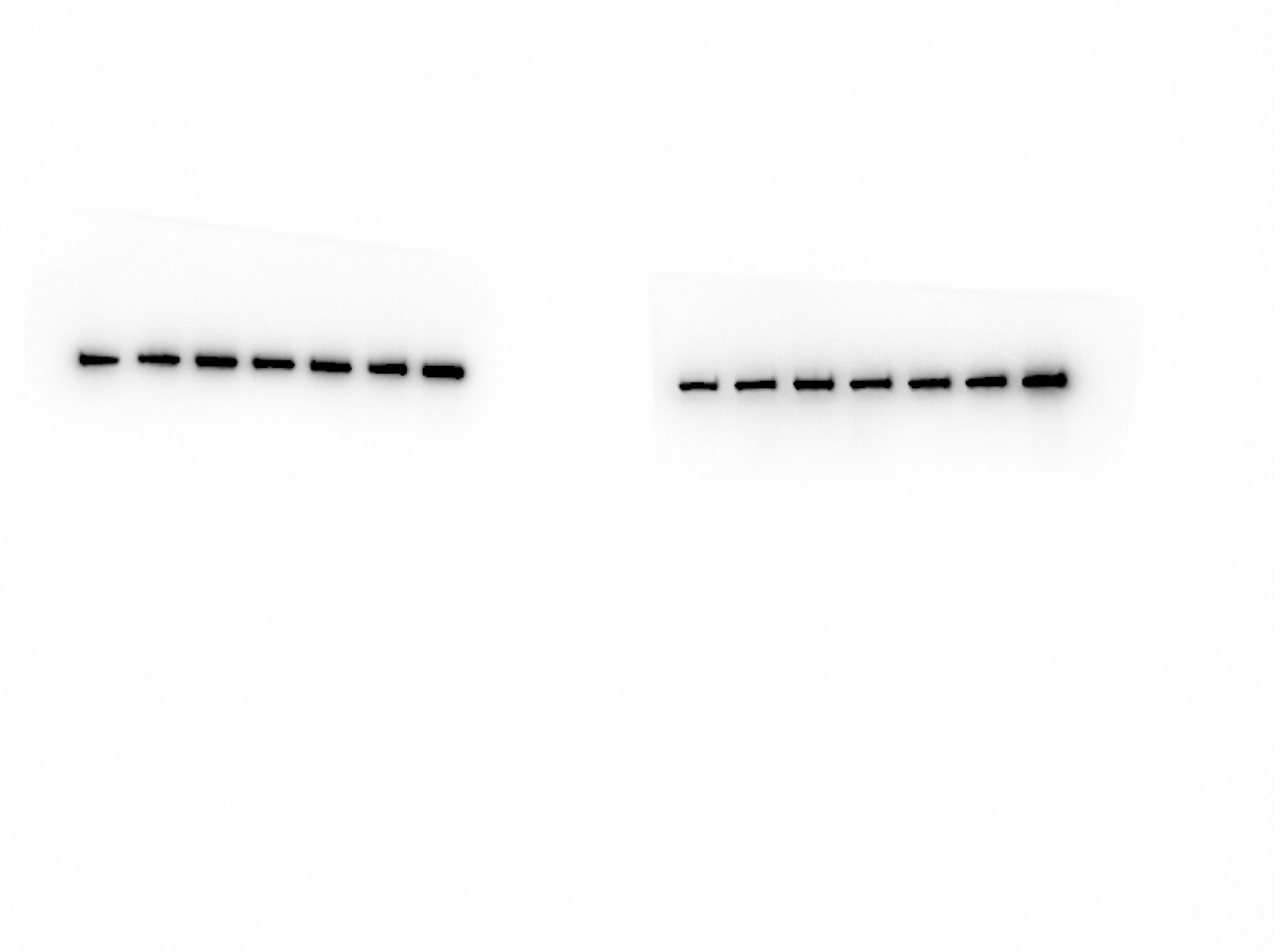

Supplement: Figure 8—source data 1. [file elife-85728-fig8-data1.zip › Figure 8-source data 1/Figure 8a CDLC4-141 iws1-flag raw data.tif]

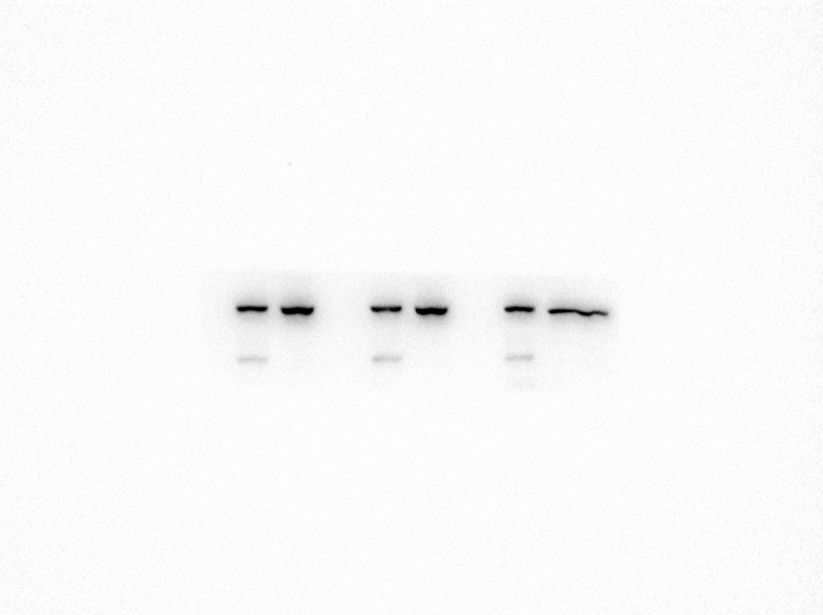

Supplement: Figure 8—source data 1. [file elife-85728-fig8-data1.zip › Figure 8-source data 1/Figure 8c cdlc120 isw1-flag raw data.tiff]

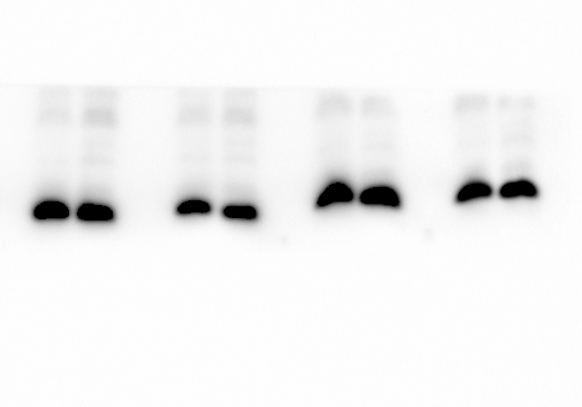

Supplement: Figure 8—source data 1. [file elife-85728-fig8-data1.zip › Figure 8-source data 1/Figure 8c cdlc61 histone raw data.tiff]

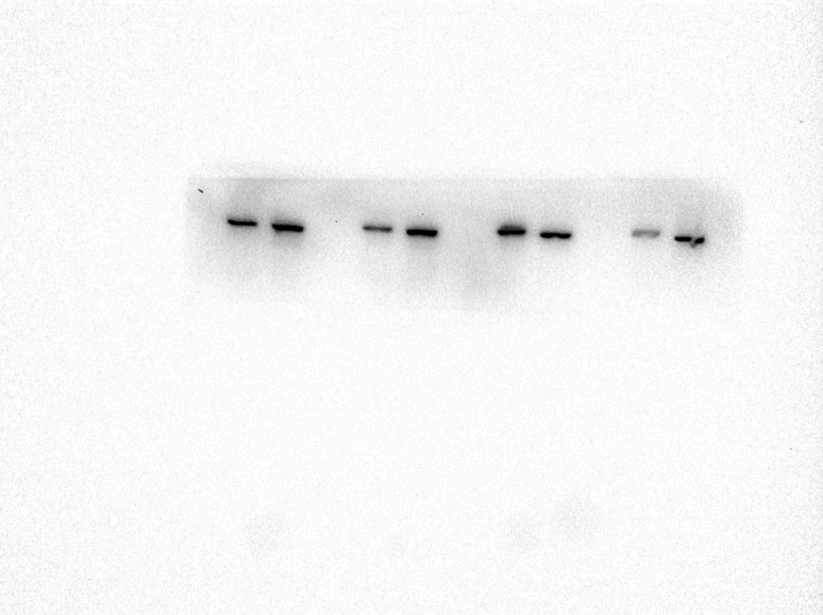

Supplement: Figure 8—source data 1. [file elife-85728-fig8-data1.zip › Figure 8-source data 1/Figure 8c cdlc61 isw1-flag raw data.tiff]

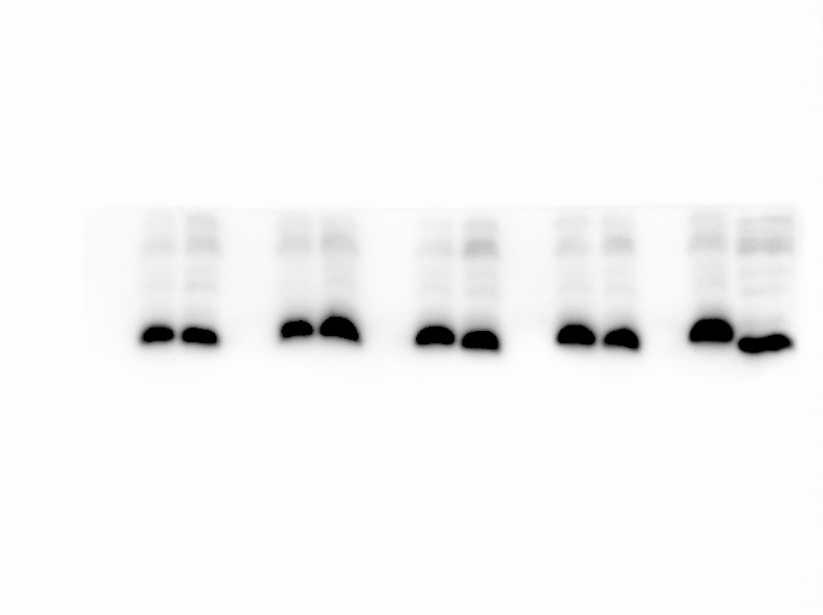

Supplement: Figure 8—source data 1. [file elife-85728-fig8-data1.zip › Figure 8-source data 1/Figure 8c cdlc62 120 histone raw dara.tiff]

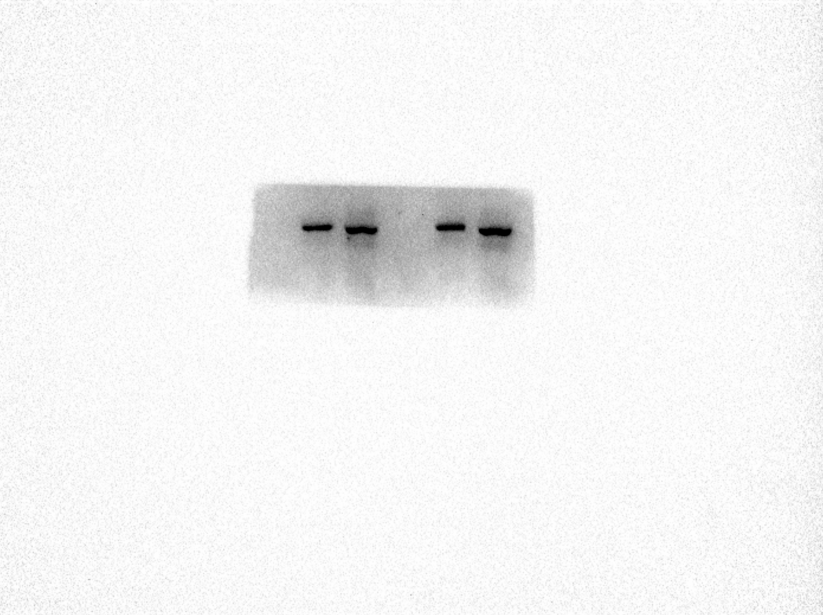

Supplement: Figure 8—source data 1. [file elife-85728-fig8-data1.zip › Figure 8-source data 1/Figure 8c cdlc62 isw1-flag raw data.tiff]

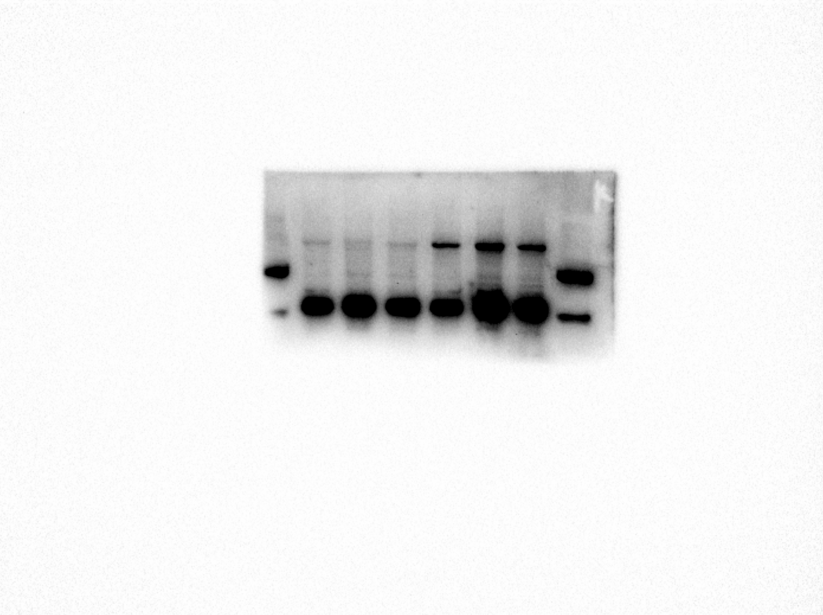

Supplement: Figure 8—source data 1. [file elife-85728-fig8-data1.zip › Figure 8-source data 1/Figure 8d isw1 Kac raw data.tiff]

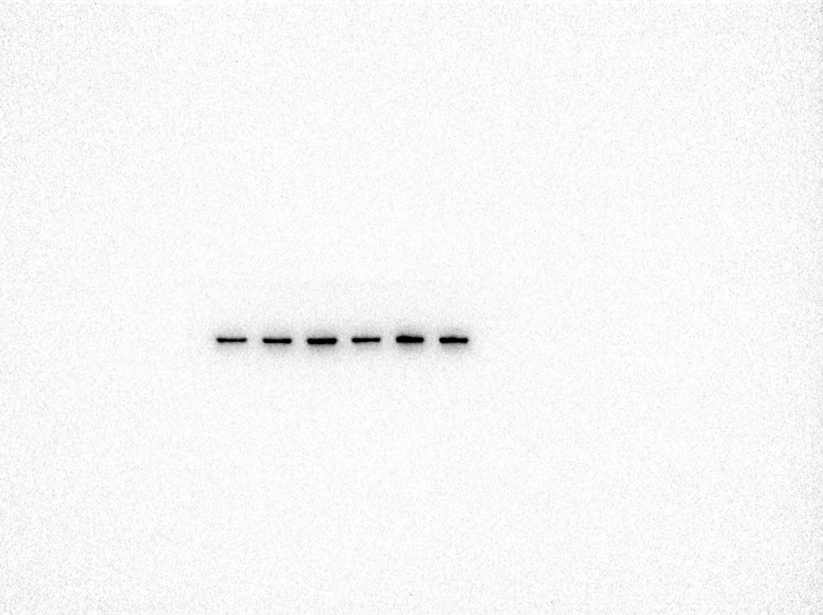

Supplement: Figure 8—source data 1. [file elife-85728-fig8-data1.zip › Figure 8-source data 1/Figure 8d isw1-flag raw data.tiff]
