# Supplementary material for: Interplay between acetylation and ubiquitination of imitation switch chromatin remodeler Isw1 confers multidrug resistance in Cryptococcus neoformans
Source: eLife. 2024 Jan 22;13:e85728. doi: 10.7554/eLife.85728 (PMC10834027; doi:10.7554/eLife.85728)
Supplement: Figure 2—source data 1. [file elife-85728-fig2-data1.zip › Figure 2-source data 1/Figure 2-source data 2.pptx]

## Slide 1
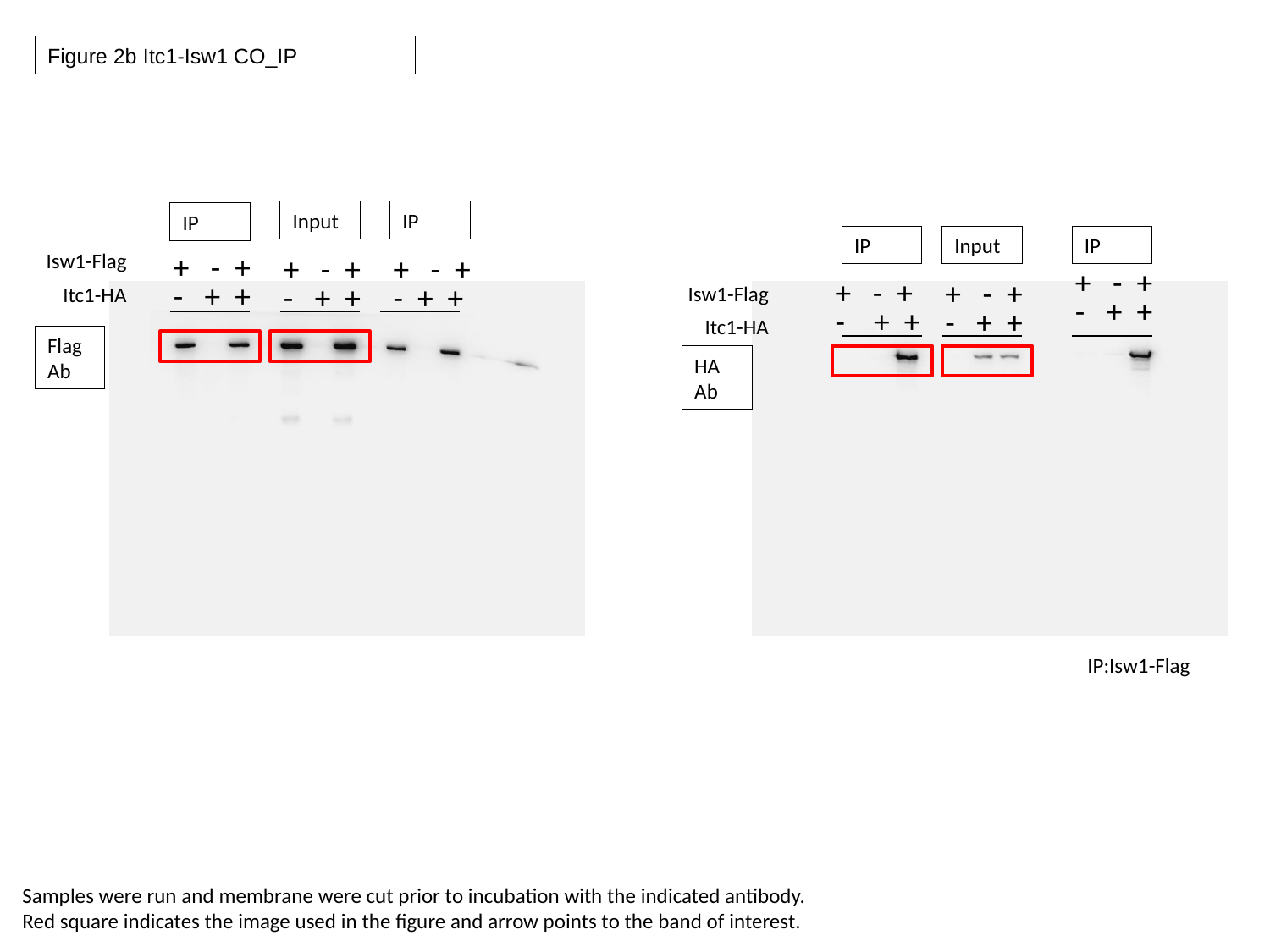

Figure 2b Itc1-Isw1 CO_IP
Input
IP
IP
IP
Input
IP
Isw1-Flag
+ - +
+ - +
+ - +
+ - +
+ - +
+ - +
- + +
- + +
- + +
Isw1-Flag
Itc1-HA
- + +
- + +
- + +
Itc1-HA
Flag
Ab
HA
Ab
IP:Isw1-Flag
Samples were run and membrane were cut prior to incubation with the indicated antibody.
Red square indicates the image used in the figure and arrow points to the band of interest.

## Slide 2
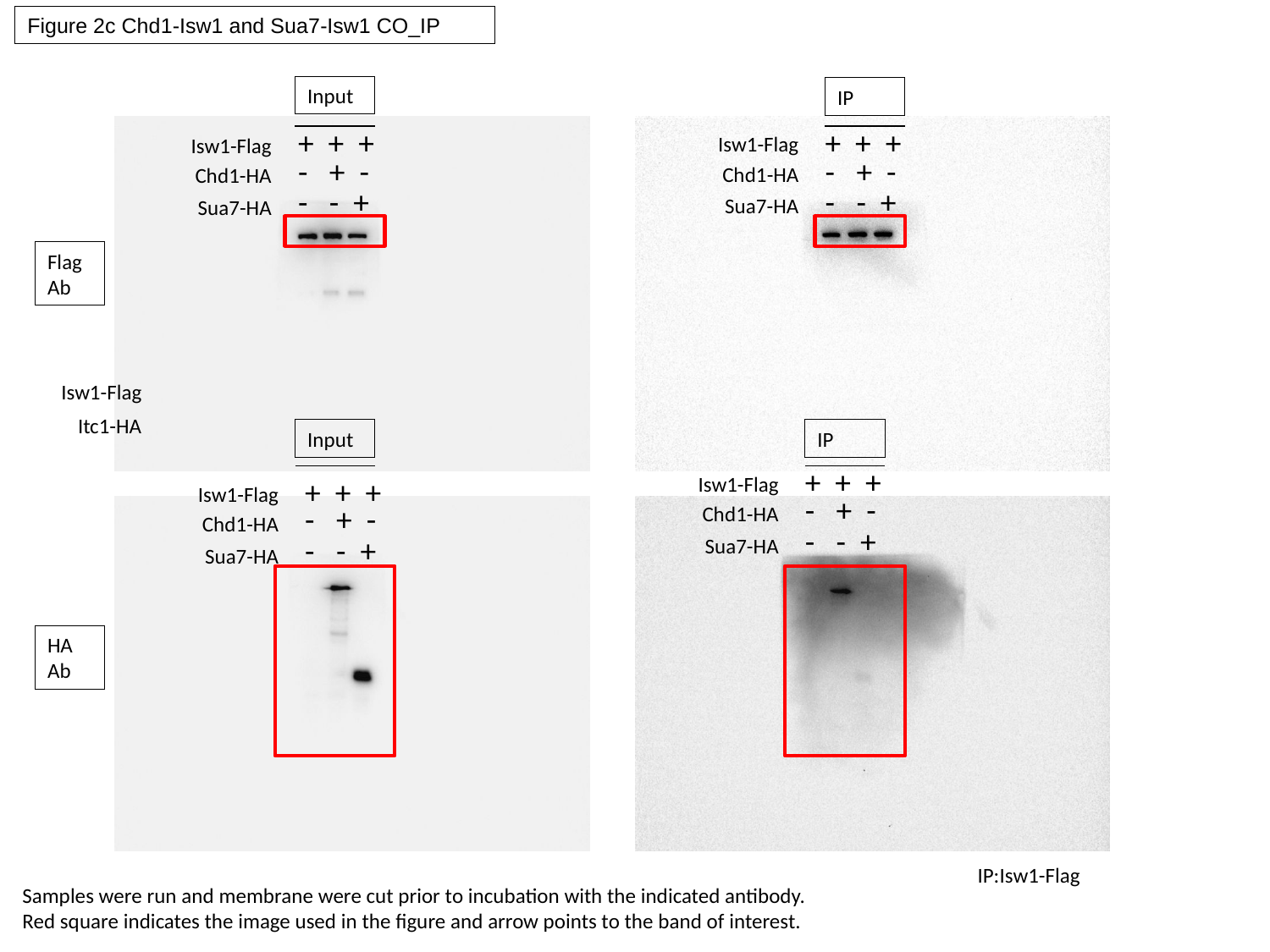

Figure 2c Chd1-Isw1 and Sua7-Isw1 CO_IP
Input
IP
+ + +
+ + +
Isw1-Flag
Isw1-Flag
- + -
- + -
Chd1-HA
Chd1-HA
- - +
- - +
Sua7-HA
Sua7-HA
Flag
Ab
Isw1-Flag
Itc1-HA
Input
IP
+ + +
Isw1-Flag
+ + +
Isw1-Flag
- + -
- + -
Chd1-HA
Chd1-HA
- - +
- - +
Sua7-HA
Sua7-HA
HA
Ab
IP:Isw1-Flag
Samples were run and membrane were cut prior to incubation with the indicated antibody.
Red square indicates the image used in the figure and arrow points to the band of interest.
