# Supplementary material for: Interplay between acetylation and ubiquitination of imitation switch chromatin remodeler Isw1 confers multidrug resistance in Cryptococcus neoformans
Source: eLife. 2024 Jan 22;13:e85728. doi: 10.7554/eLife.85728 (PMC10834027; doi:10.7554/eLife.85728)
Supplement: Figure 3—figure supplement 1—source data 1. [file elife-85728-fig3-figsupp1-data1.zip › Figure 3-figure supplement 1-source data 1/Figure 3-figure supplement 1-source data 2.pptx]

## Slide 1
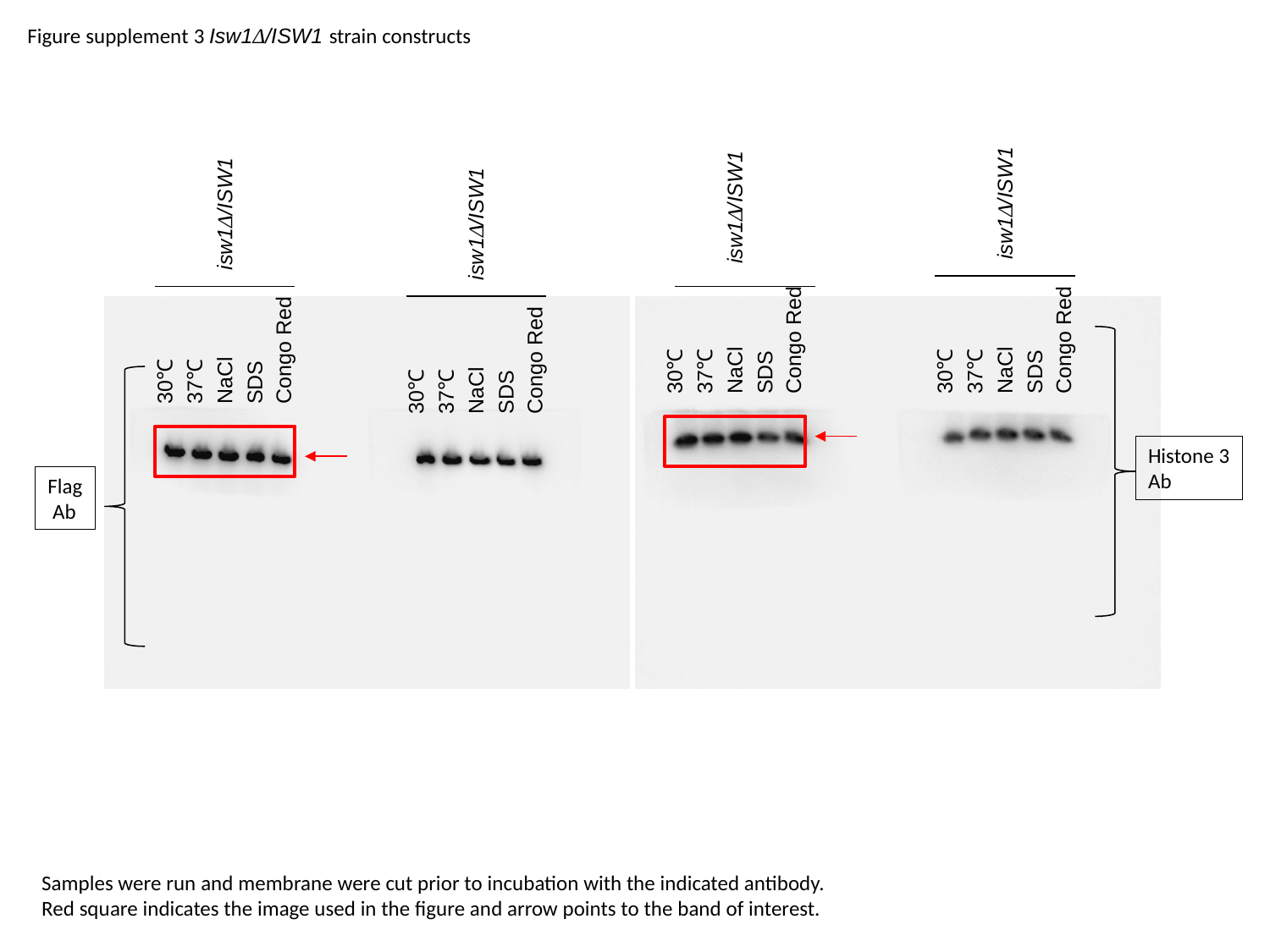

Figure supplement 3 Isw1Δ/ISW1 strain constructs
isw1Δ/ISW1
isw1Δ/ISW1
isw1Δ/ISW1
isw1Δ/ISW1
Congo Red
Congo Red
Congo Red
Congo Red
NaCl
NaCl
30℃
37℃
30℃
37℃
SDS
SDS
NaCl
30℃
37℃
SDS
NaCl
30℃
37℃
SDS
Histone 3
Ab
Flag
 Ab
Samples were run and membrane were cut prior to incubation with the indicated antibody.
Red square indicates the image used in the figure and arrow points to the band of interest.

## Slide 2
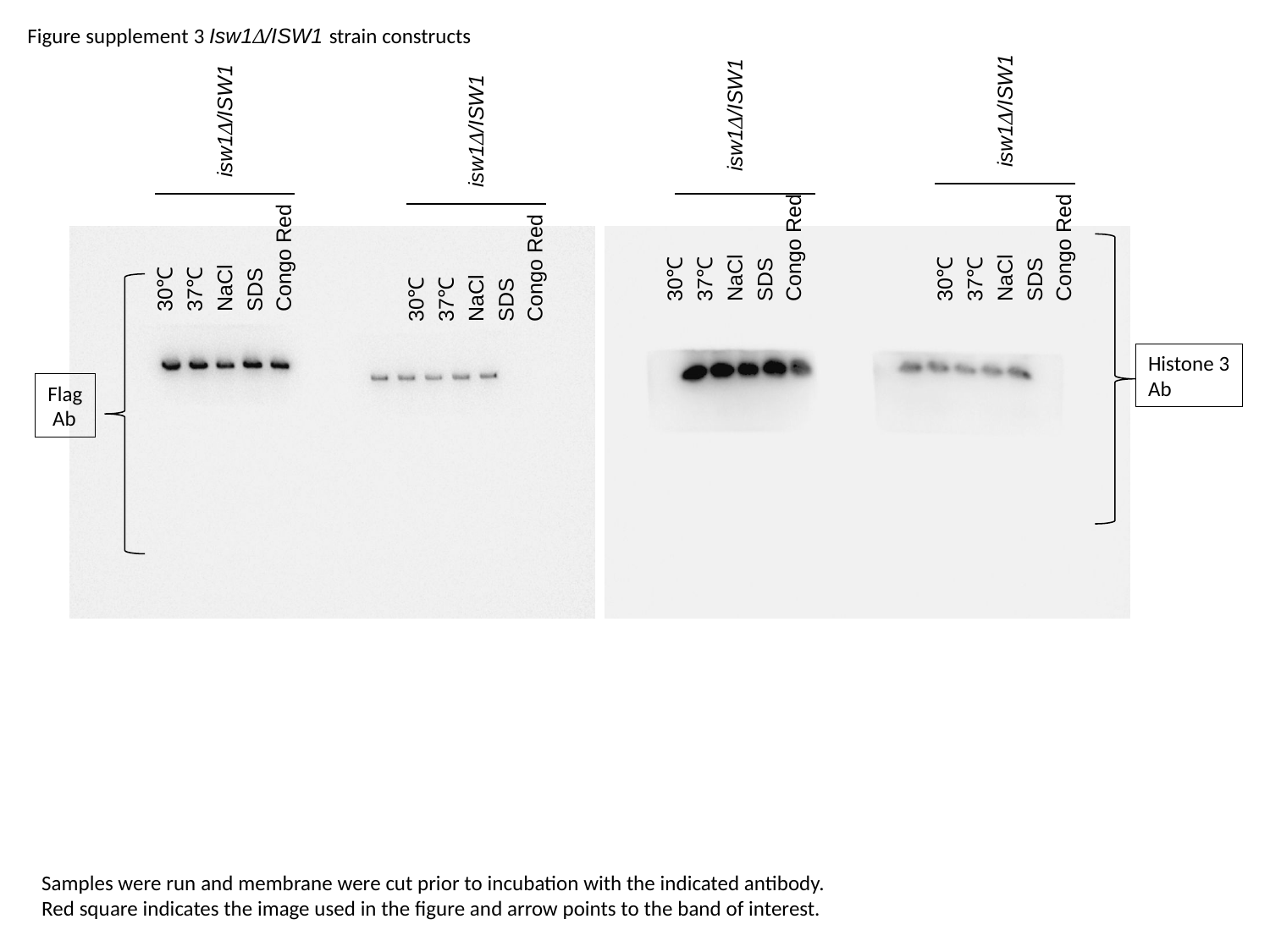

Figure supplement 3 Isw1Δ/ISW1 strain constructs
isw1Δ/ISW1
isw1Δ/ISW1
isw1Δ/ISW1
isw1Δ/ISW1
Congo Red
Congo Red
Congo Red
Congo Red
NaCl
NaCl
30℃
37℃
30℃
37℃
SDS
SDS
NaCl
30℃
37℃
SDS
NaCl
30℃
37℃
SDS
Histone 3
Ab
Flag
 Ab
Samples were run and membrane were cut prior to incubation with the indicated antibody.
Red square indicates the image used in the figure and arrow points to the band of interest.
