# Supplementary material for: Interplay between acetylation and ubiquitination of imitation switch chromatin remodeler Isw1 confers multidrug resistance in Cryptococcus neoformans
Source: eLife. 2024 Jan 22;13:e85728. doi: 10.7554/eLife.85728 (PMC10834027; doi:10.7554/eLife.85728)
Supplement: Figure 4—source data 1. [file elife-85728-fig4-data1.zip › Figure 4-source data 1/Figure 4-source data 5.pptx]

## Slide 1
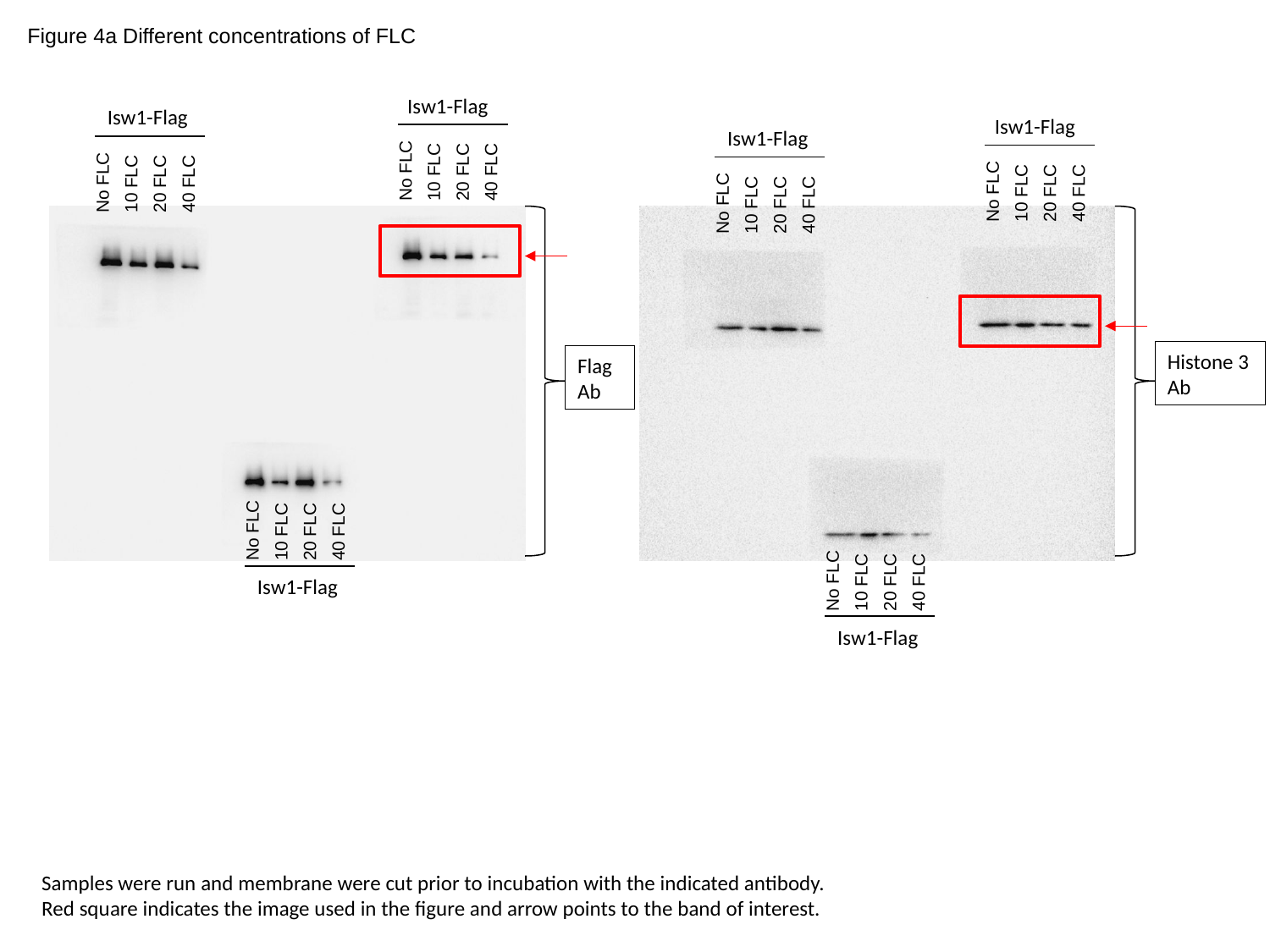

Figure 4a Different concentrations of FLC
Isw1-Flag
Isw1-Flag
Isw1-Flag
Isw1-Flag
No FLC
10 FLC
20 FLC
40 FLC
No FLC
10 FLC
20 FLC
40 FLC
No FLC
10 FLC
20 FLC
40 FLC
No FLC
10 FLC
20 FLC
40 FLC
Histone 3
Ab
Flag
Ab
No FLC
10 FLC
20 FLC
40 FLC
No FLC
10 FLC
20 FLC
40 FLC
Isw1-Flag
Isw1-Flag
Samples were run and membrane were cut prior to incubation with the indicated antibody.
Red square indicates the image used in the figure and arrow points to the band of interest.

## Slide 2
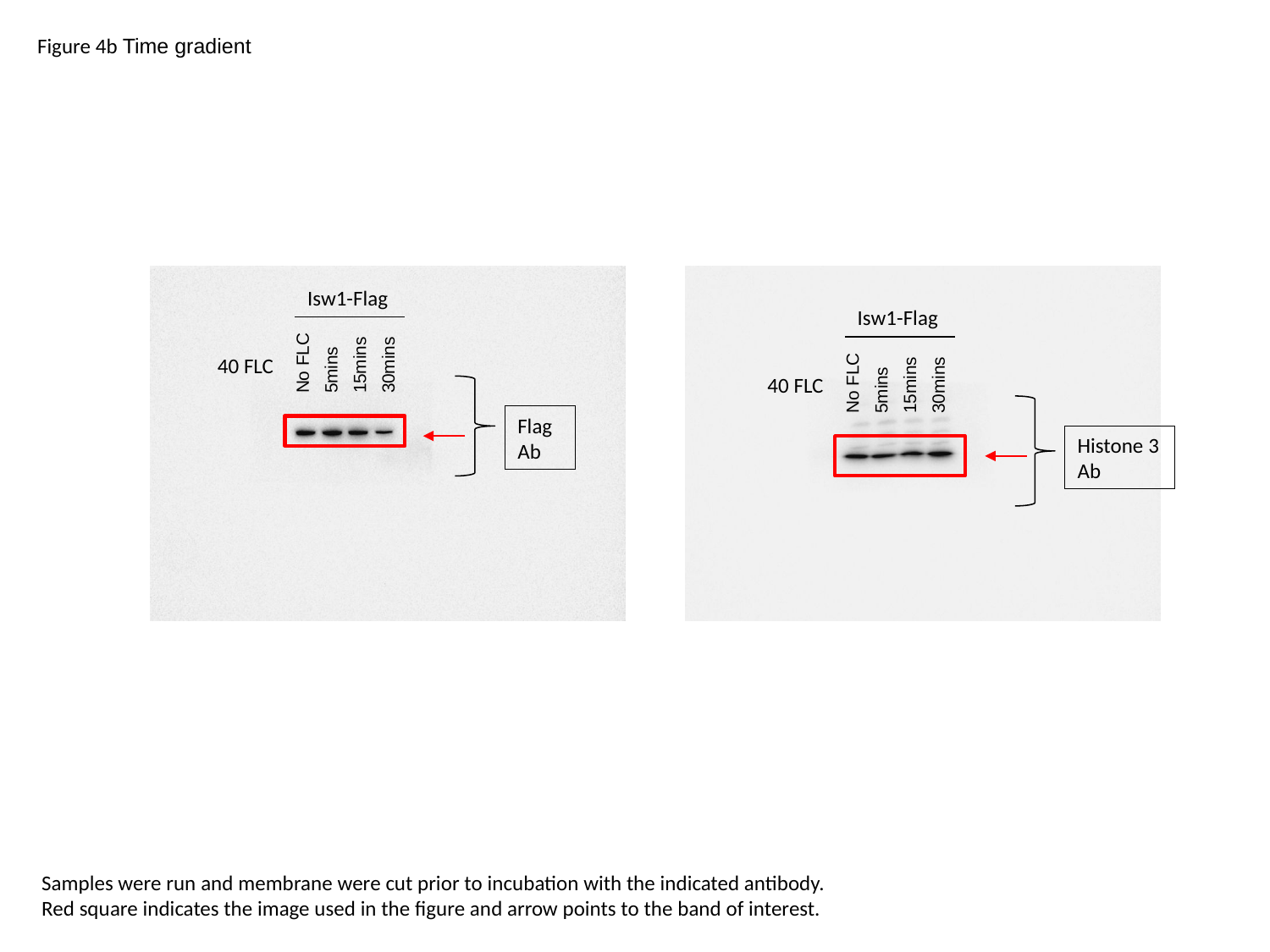

Figure 4b Time gradient
Isw1-Flag
Isw1-Flag
No FLC
5mins
15mins
30mins
40 FLC
No FLC
5mins
15mins
30mins
40 FLC
Flag
Ab
Histone 3
Ab
Samples were run and membrane were cut prior to incubation with the indicated antibody.
Red square indicates the image used in the figure and arrow points to the band of interest.

## Slide 3
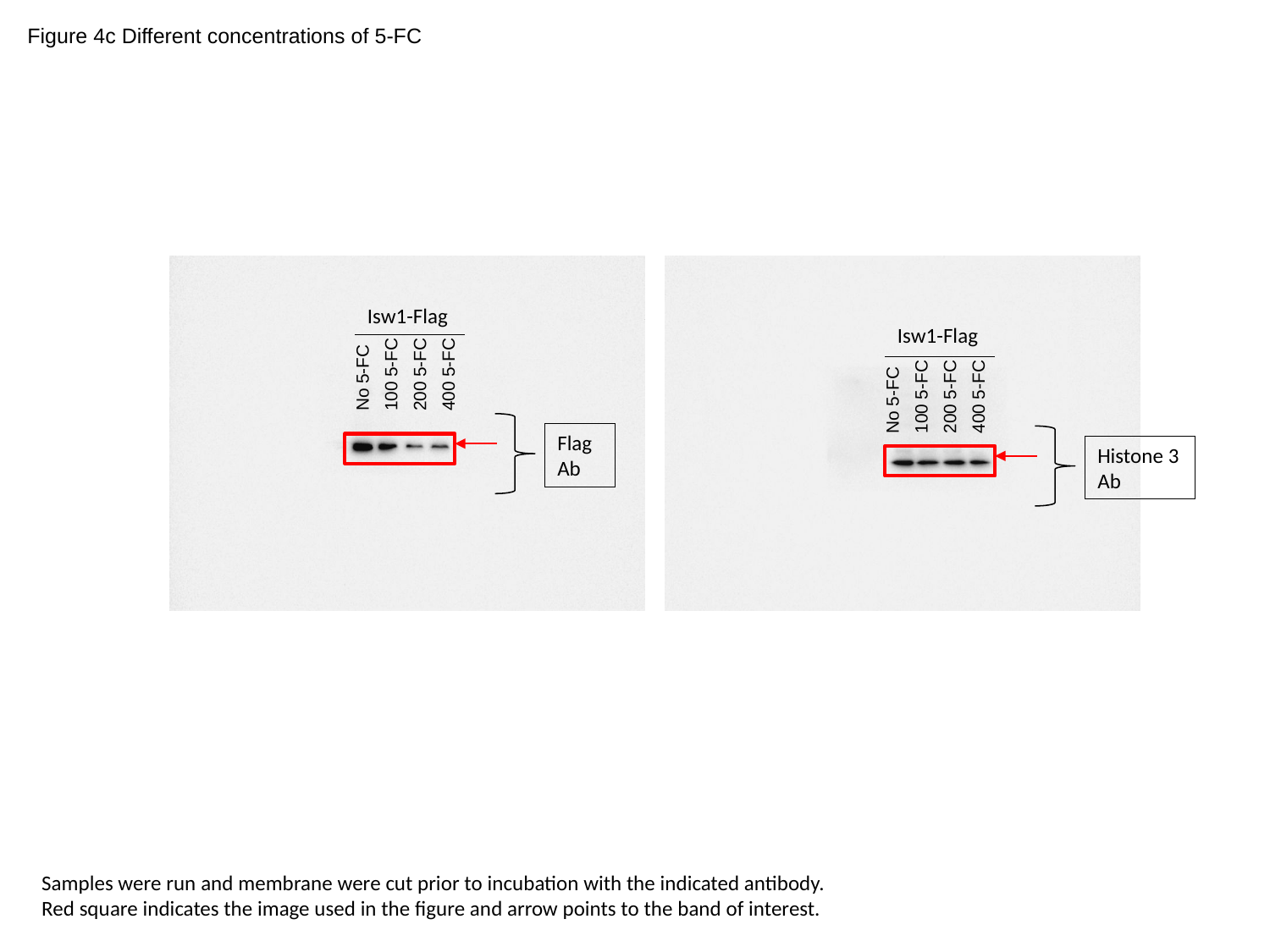

Figure 4c Different concentrations of 5-FC
Isw1-Flag
Isw1-Flag
No 5-FC
100 5-FC
200 5-FC
400 5-FC
No 5-FC
100 5-FC
200 5-FC
400 5-FC
Flag
Ab
Histone 3
Ab
Samples were run and membrane were cut prior to incubation with the indicated antibody.
Red square indicates the image used in the figure and arrow points to the band of interest.

## Slide 4
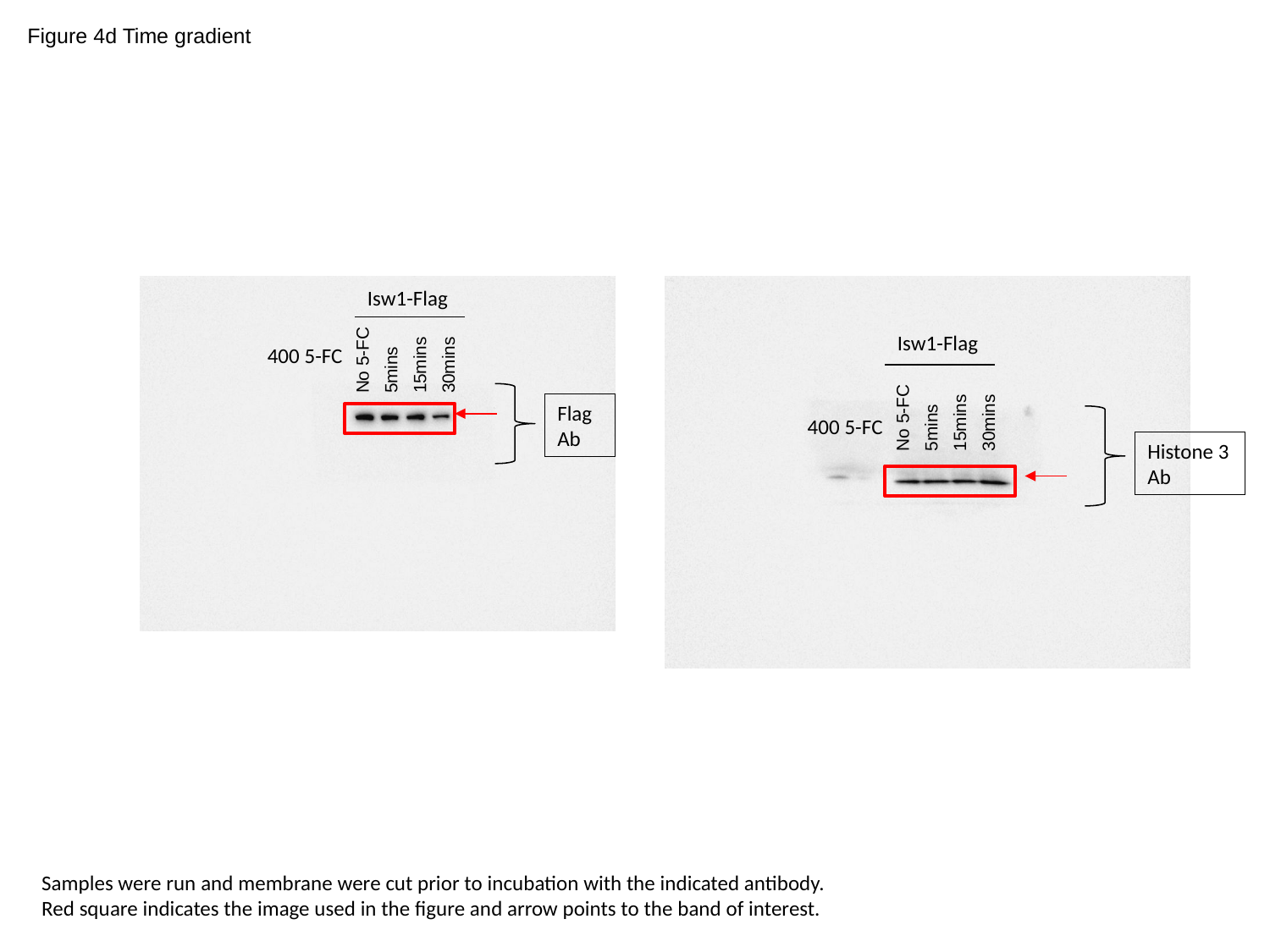

Figure 4d Time gradient
Isw1-Flag
Isw1-Flag
No 5-FC
5mins
15mins
30mins
400 5-FC
No 5-FC
5mins
15mins
30mins
Flag
Ab
400 5-FC
Histone 3
Ab
Samples were run and membrane were cut prior to incubation with the indicated antibody.
Red square indicates the image used in the figure and arrow points to the band of interest.
