# Supplementary material for: Interplay between acetylation and ubiquitination of imitation switch chromatin remodeler Isw1 confers multidrug resistance in Cryptococcus neoformans
Source: eLife. 2024 Jan 22;13:e85728. doi: 10.7554/eLife.85728 (PMC10834027; doi:10.7554/eLife.85728)
Supplement: Figure 5—source data 1. [file elife-85728-fig5-data1.zip › Figure 5-source data 1/Figure 5-source data 8.pptx]

## Slide 1
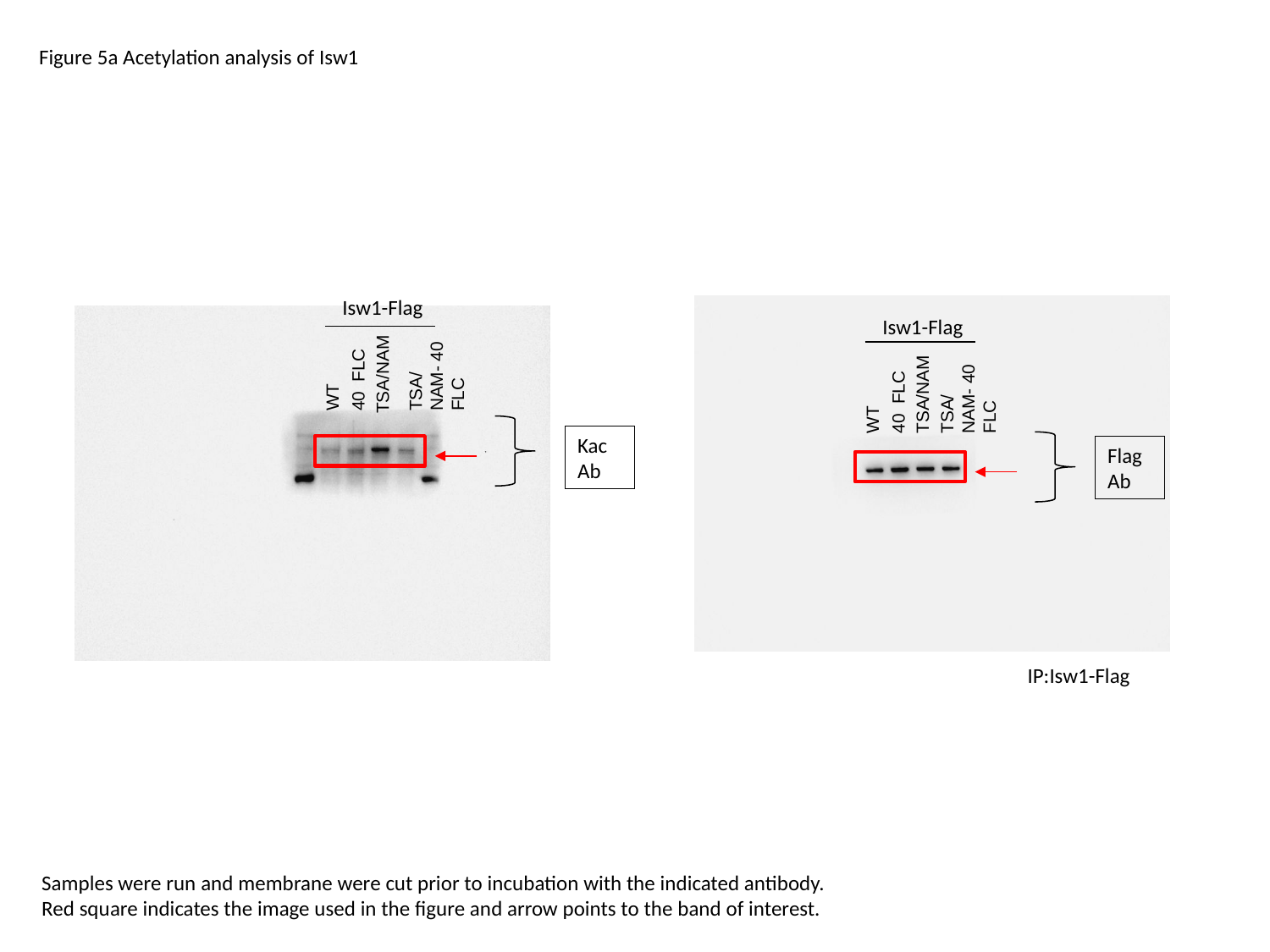

Figure 5a Acetylation analysis of Isw1
Isw1-Flag
Isw1-Flag
TSA/NAM- 40 FLC
WT
40 FLC
TSA/NAM
TSA/NAM- 40 FLC
WT
40 FLC
TSA/NAM
Kac
Ab
Flag
Ab
IP:Isw1-Flag
Samples were run and membrane were cut prior to incubation with the indicated antibody.
Red square indicates the image used in the figure and arrow points to the band of interest.

## Slide 2
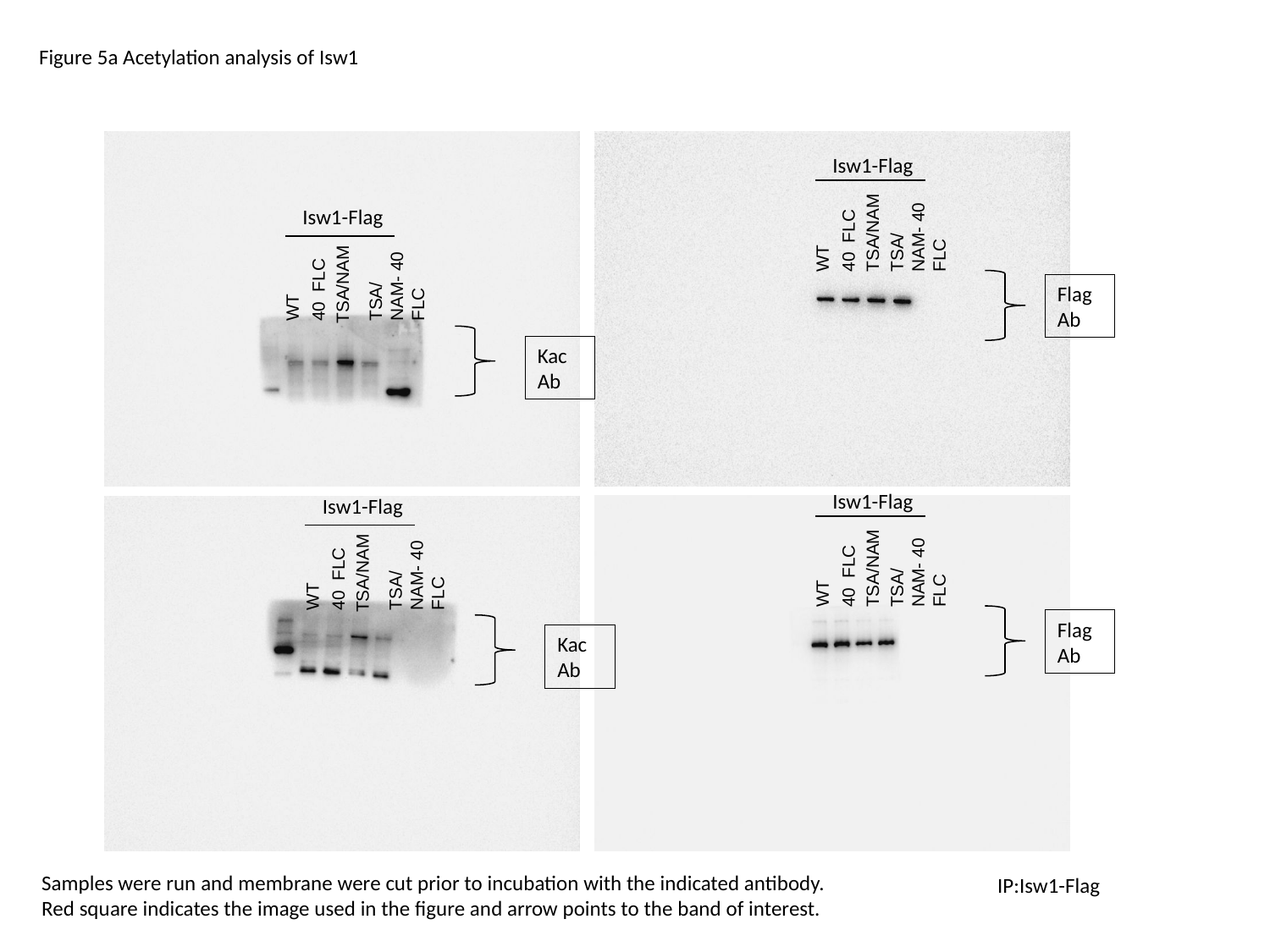

Figure 5a Acetylation analysis of Isw1
Isw1-Flag
Isw1-Flag
TSA/NAM- 40 FLC
WT
40 FLC
TSA/NAM
TSA/NAM- 40 FLC
WT
40 FLC
TSA/NAM
Flag
Ab
Kac
Ab
Isw1-Flag
Isw1-Flag
TSA/NAM- 40 FLC
TSA/NAM- 40 FLC
WT
40 FLC
TSA/NAM
WT
40 FLC
TSA/NAM
Flag
Ab
Kac
Ab
Samples were run and membrane were cut prior to incubation with the indicated antibody.
Red square indicates the image used in the figure and arrow points to the band of interest.
IP:Isw1-Flag

## Slide 3
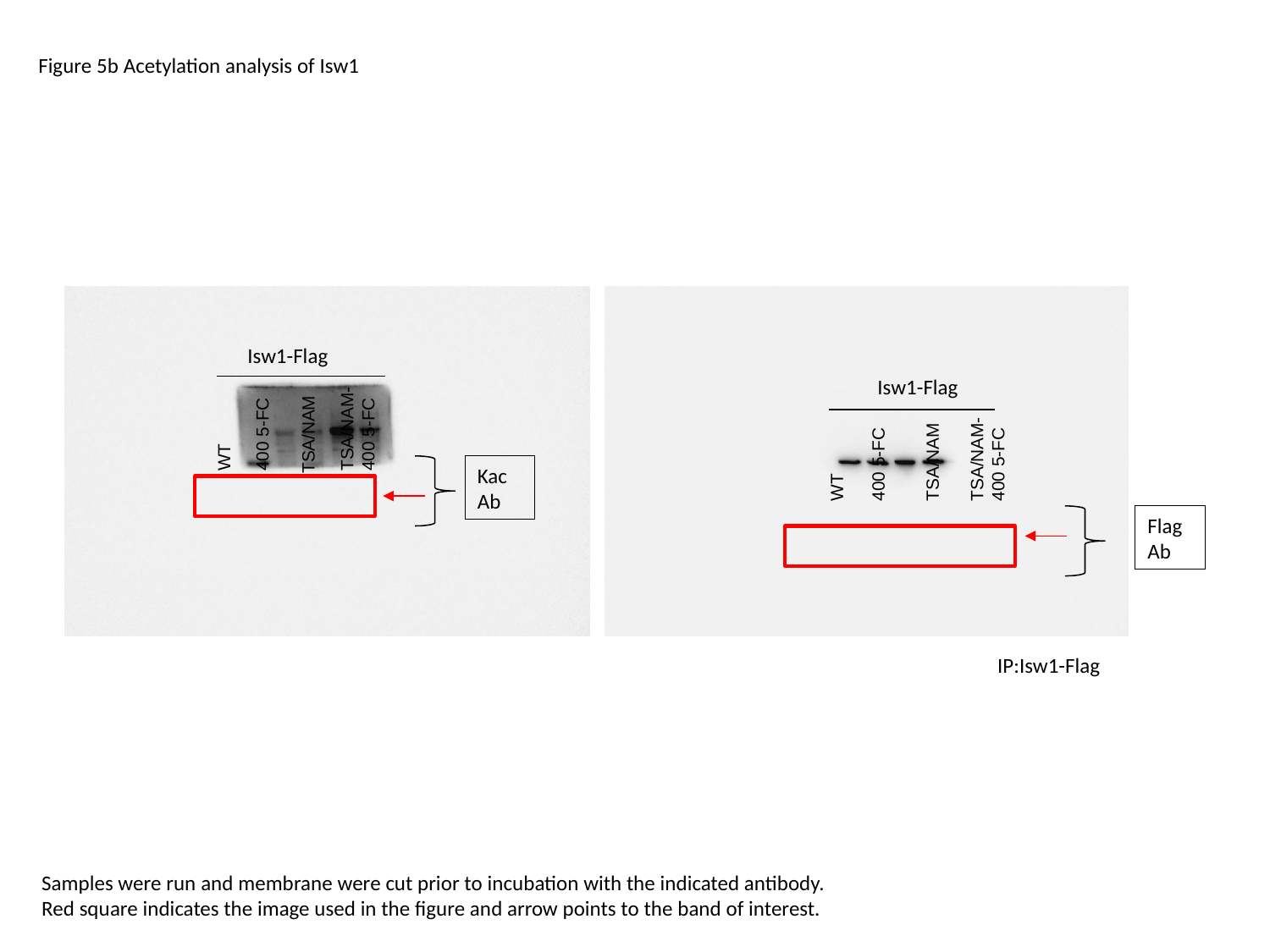

Figure 5b Acetylation analysis of Isw1
Isw1-Flag
Isw1-Flag
TSA/NAM- 400 5-FC
WT
400 5-FC
TSA/NAM
TSA/NAM- 400 5-FC
WT
400 5-FC
TSA/NAM
Kac
Ab
Flag
Ab
IP:Isw1-Flag
Samples were run and membrane were cut prior to incubation with the indicated antibody.
Red square indicates the image used in the figure and arrow points to the band of interest.

## Slide 4
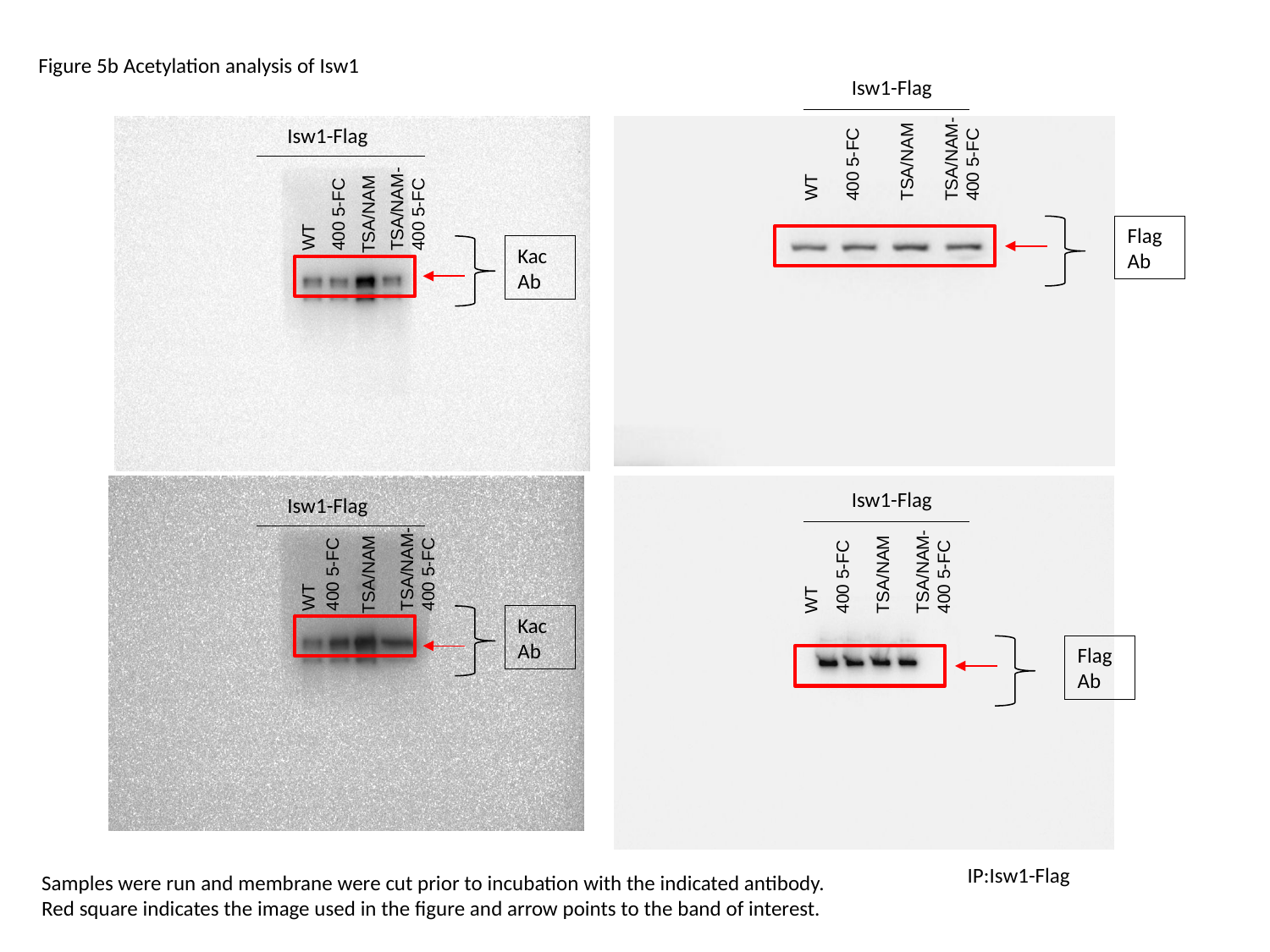

Figure 5b Acetylation analysis of Isw1
Isw1-Flag
Isw1-Flag
TSA/NAM- 400 5-FC
WT
400 5-FC
TSA/NAM
TSA/NAM- 400 5-FC
WT
400 5-FC
TSA/NAM
Flag
Ab
Kac
Ab
Isw1-Flag
Isw1-Flag
TSA/NAM- 400 5-FC
TSA/NAM- 400 5-FC
WT
400 5-FC
TSA/NAM
WT
400 5-FC
TSA/NAM
Kac
Ab
Flag
Ab
IP:Isw1-Flag
Samples were run and membrane were cut prior to incubation with the indicated antibody.
Red square indicates the image used in the figure and arrow points to the band of interest.

## Slide 5
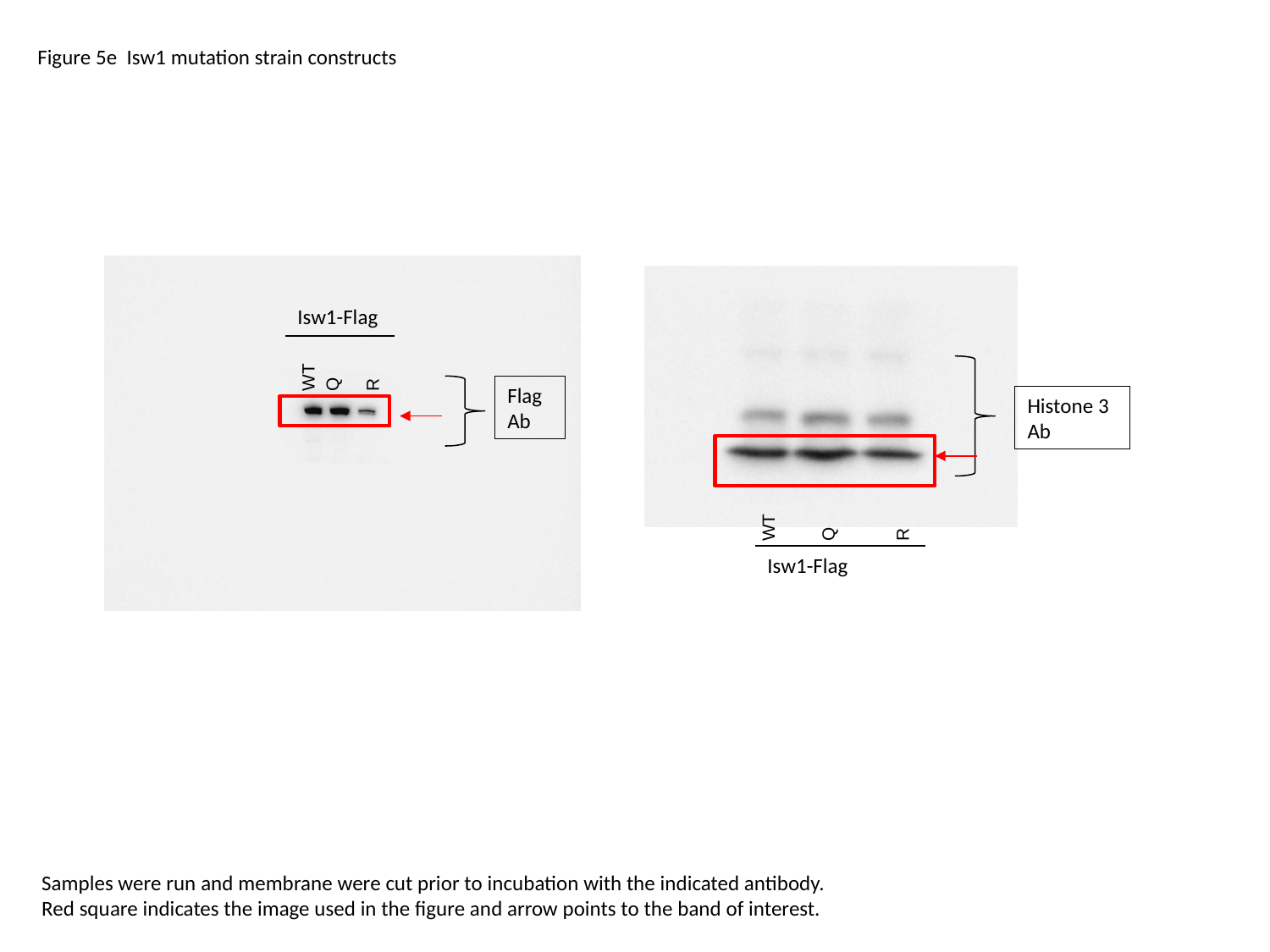

Figure 5e Isw1 mutation strain constructs
Isw1-Flag
WT
Q
R
Flag
Ab
Histone 3
Ab
WT
Q
R
Isw1-Flag
Samples were run and membrane were cut prior to incubation with the indicated antibody.
Red square indicates the image used in the figure and arrow points to the band of interest.

## Slide 6
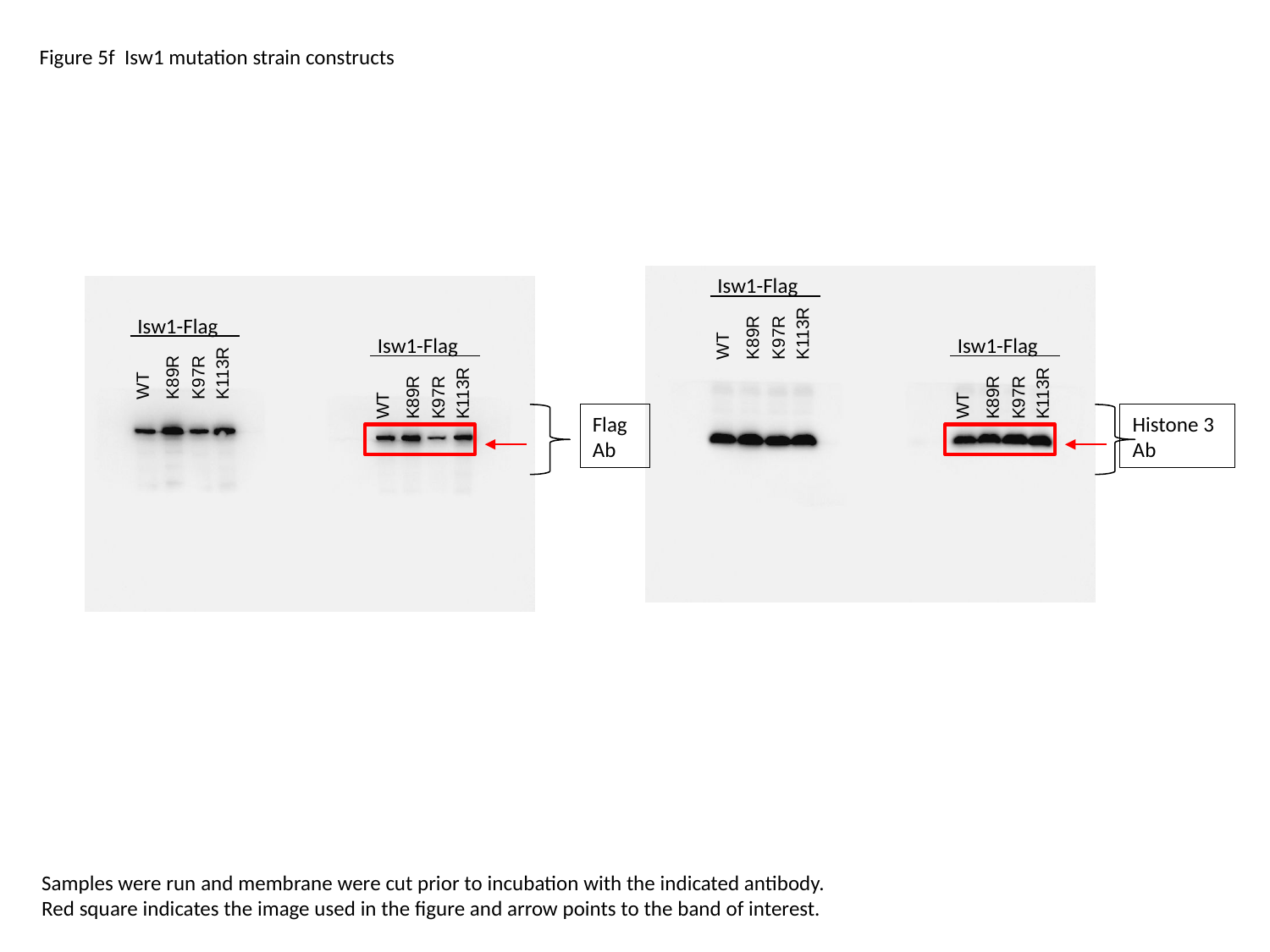

Figure 5f Isw1 mutation strain constructs
Isw1-Flag
Isw1-Flag
K113R
K89R
K97R
Isw1-Flag
Isw1-Flag
WT
K113R
K89R
K97R
WT
K113R
K113R
K89R
K97R
K89R
K97R
WT
WT
Flag
Ab
Histone 3
Ab
Samples were run and membrane were cut prior to incubation with the indicated antibody.
Red square indicates the image used in the figure and arrow points to the band of interest.

## Slide 7
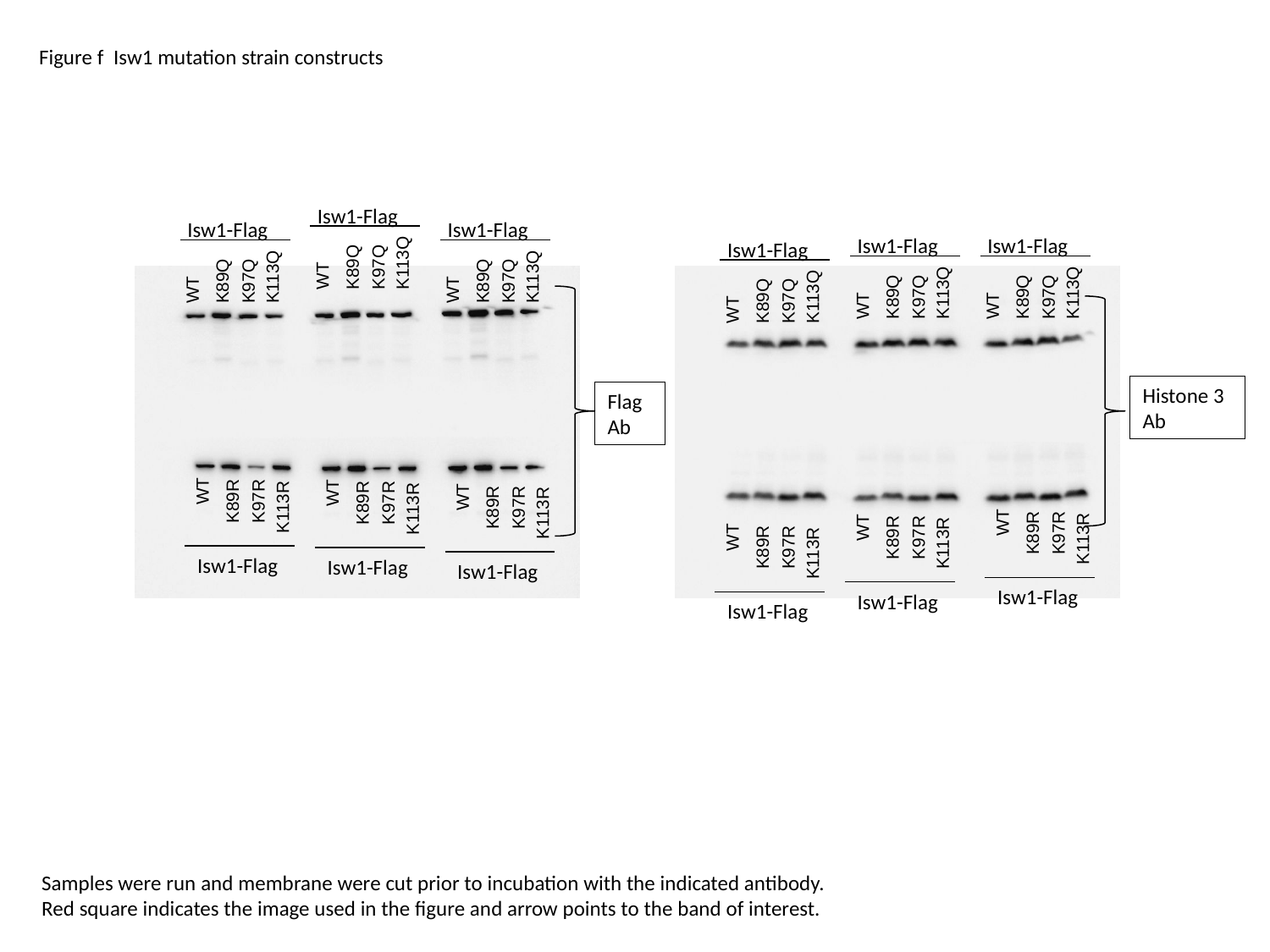

Figure f Isw1 mutation strain constructs
Isw1-Flag
Isw1-Flag
Isw1-Flag
Isw1-Flag
Isw1-Flag
Isw1-Flag
K113Q
K89Q
K97Q
K113Q
K113Q
WT
K89Q
K97Q
K89Q
K97Q
WT
WT
K113Q
K113Q
K113Q
K89Q
K97Q
K89Q
K97Q
K89Q
K97Q
WT
WT
WT
Histone 3
Ab
Flag
Ab
WT
WT
WT
K89R
K97R
K89R
K97R
K113R
K89R
K97R
K113R
K113R
WT
WT
K89R
K97R
K89R
K97R
K113R
WT
K113R
K89R
K97R
K113R
Isw1-Flag
Isw1-Flag
Isw1-Flag
Isw1-Flag
Isw1-Flag
Isw1-Flag
Samples were run and membrane were cut prior to incubation with the indicated antibody.
Red square indicates the image used in the figure and arrow points to the band of interest.

## Slide 8
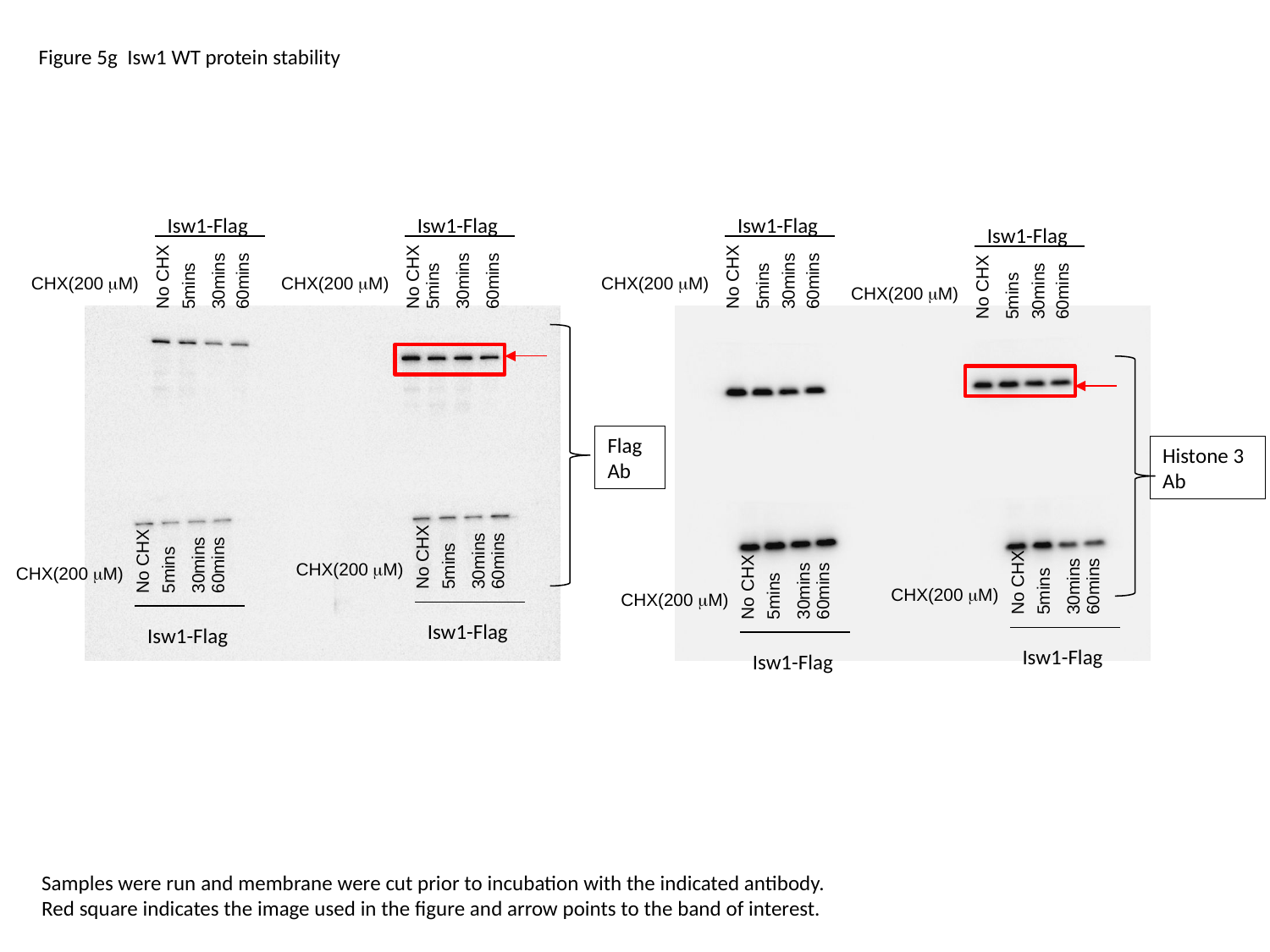

Figure 5g Isw1 WT protein stability
Isw1-Flag
Isw1-Flag
Isw1-Flag
Isw1-Flag
No CHX
No CHX
No CHX
30mins
30mins
30mins
60mins
60mins
60mins
No CHX
CHX(200 μM)
CHX(200 μM)
CHX(200 μM)
5mins
5mins
5mins
30mins
60mins
CHX(200 μM)
5mins
Flag
Ab
Histone 3
Ab
5mins
30mins
No CHX
60mins
5mins
30mins
No CHX
60mins
CHX(200 μM)
CHX(200 μM)
5mins
30mins
No CHX
60mins
5mins
30mins
No CHX
60mins
CHX(200 μM)
CHX(200 μM)
Isw1-Flag
Isw1-Flag
Isw1-Flag
Isw1-Flag
Samples were run and membrane were cut prior to incubation with the indicated antibody.
Red square indicates the image used in the figure and arrow points to the band of interest.

## Slide 9
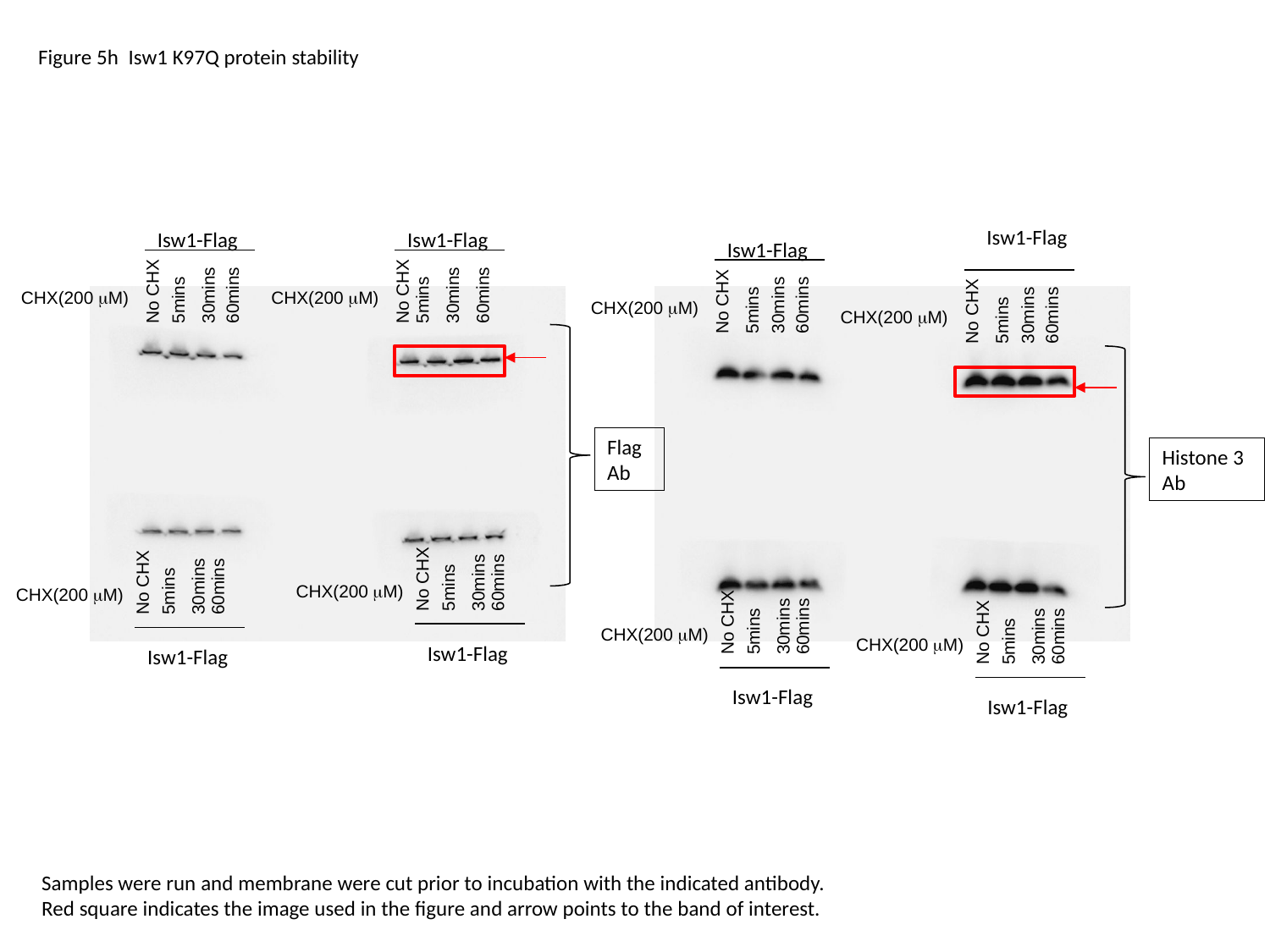

Figure 5h Isw1 K97Q protein stability
Isw1-Flag
Isw1-Flag
Isw1-Flag
Isw1-Flag
No CHX
No CHX
30mins
30mins
60mins
60mins
No CHX
CHX(200 μM)
CHX(200 μM)
30mins
5mins
5mins
60mins
No CHX
CHX(200 μM)
5mins
30mins
60mins
CHX(200 μM)
5mins
Flag
Ab
Histone 3
Ab
5mins
30mins
No CHX
60mins
5mins
30mins
No CHX
60mins
CHX(200 μM)
CHX(200 μM)
5mins
30mins
No CHX
60mins
5mins
30mins
No CHX
60mins
CHX(200 μM)
CHX(200 μM)
Isw1-Flag
Isw1-Flag
Isw1-Flag
Isw1-Flag
Samples were run and membrane were cut prior to incubation with the indicated antibody.
Red square indicates the image used in the figure and arrow points to the band of interest.

## Slide 10
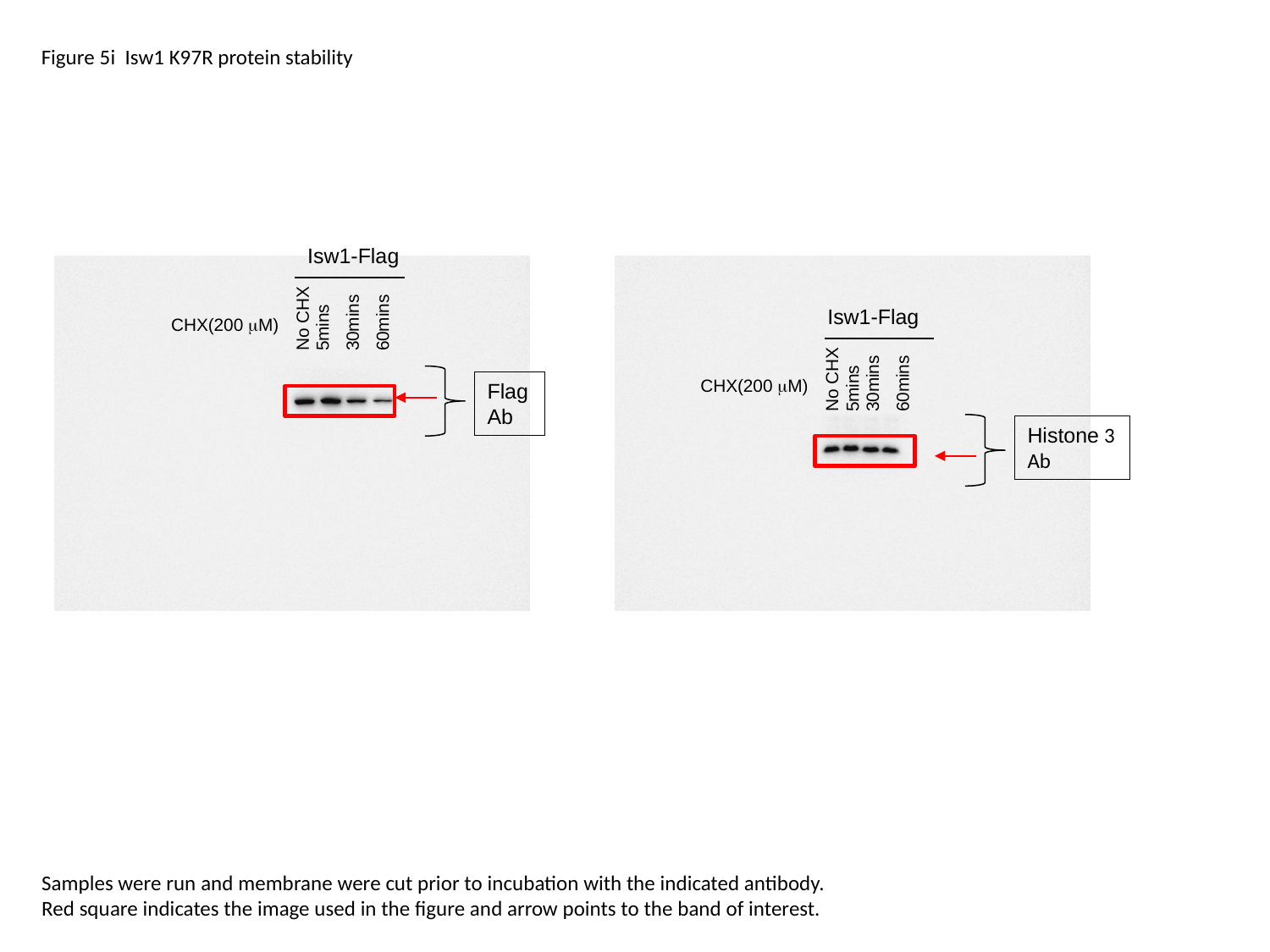

Figure 5i Isw1 K97R protein stability
Isw1-Flag
No CHX
Isw1-Flag
30mins
60mins
CHX(200 μM)
5mins
No CHX
30mins
60mins
CHX(200 μM)
5mins
Flag
Ab
Histone 3
Ab
Samples were run and membrane were cut prior to incubation with the indicated antibody.
Red square indicates the image used in the figure and arrow points to the band of interest.

## Slide 11
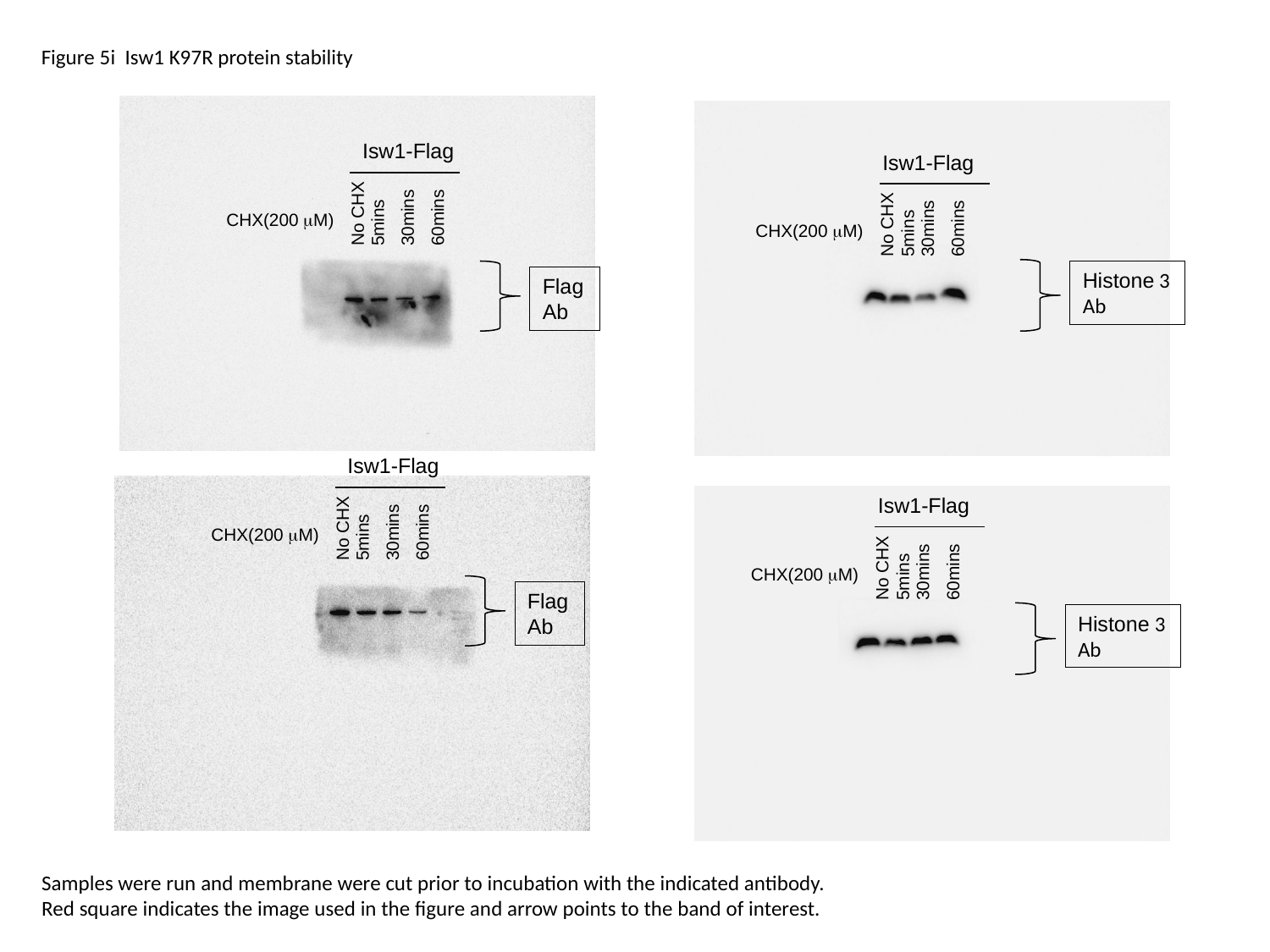

Figure 5i Isw1 K97R protein stability
Isw1-Flag
Isw1-Flag
No CHX
30mins
60mins
No CHX
CHX(200 μM)
5mins
30mins
60mins
CHX(200 μM)
5mins
Histone 3
Ab
Flag
Ab
Isw1-Flag
Isw1-Flag
No CHX
30mins
60mins
CHX(200 μM)
5mins
No CHX
30mins
60mins
CHX(200 μM)
5mins
Flag
Ab
Histone 3
Ab
Samples were run and membrane were cut prior to incubation with the indicated antibody.
Red square indicates the image used in the figure and arrow points to the band of interest.
