# Supplementary material for: Interplay between acetylation and ubiquitination of imitation switch chromatin remodeler Isw1 confers multidrug resistance in Cryptococcus neoformans
Source: eLife. 2024 Jan 22;13:e85728. doi: 10.7554/eLife.85728 (PMC10834027; doi:10.7554/eLife.85728)
Supplement: Figure 5—figure supplement 1—source data 1. [file elife-85728-fig5-figsupp1-data1.zip › Figure 5-figure supplement 1-source data 1/Figure 5-figure supplement 1-source data 3.pptx]

## Slide 1
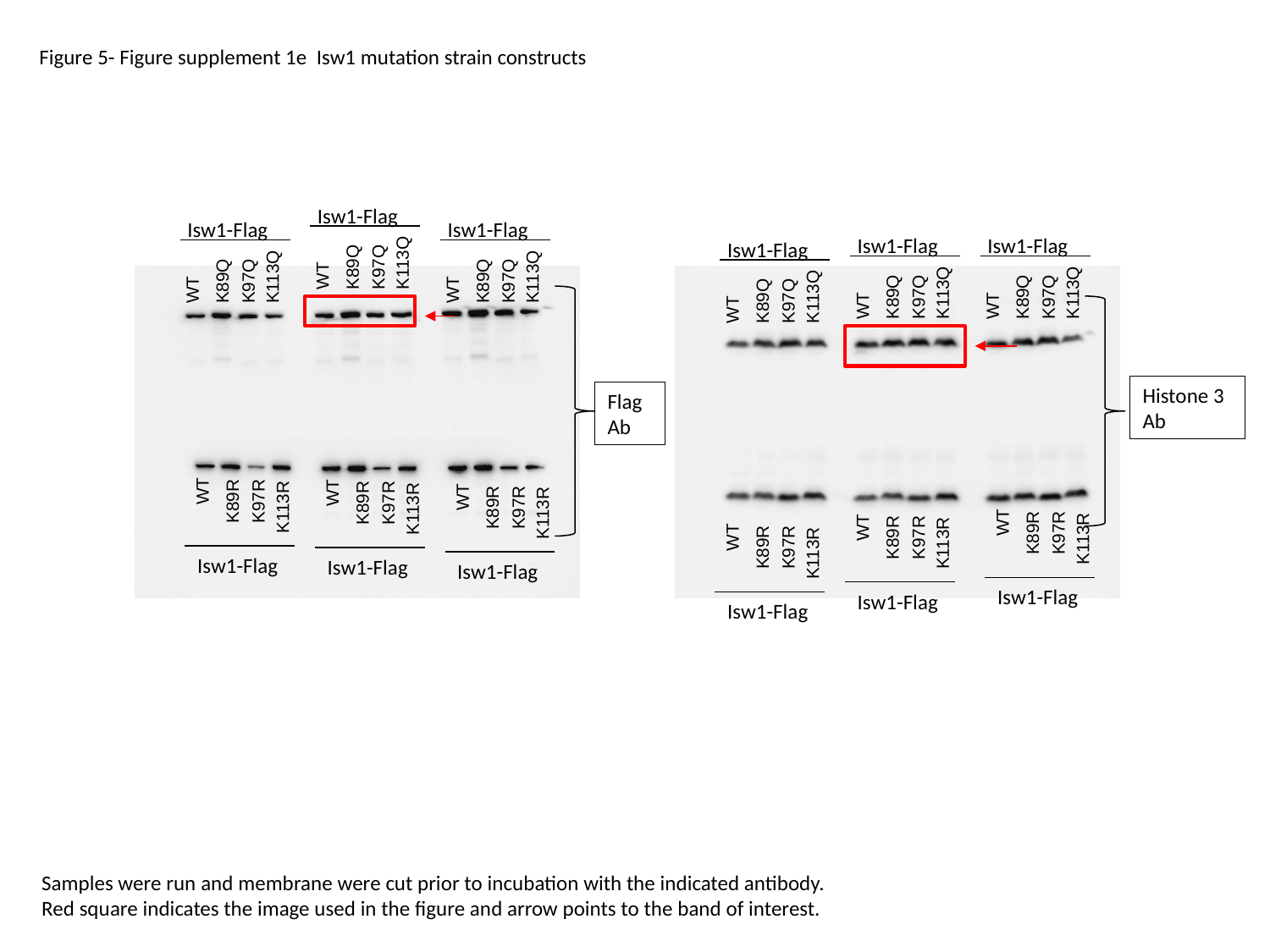

Figure 5- Figure supplement 1e Isw1 mutation strain constructs
Isw1-Flag
Isw1-Flag
Isw1-Flag
Isw1-Flag
Isw1-Flag
Isw1-Flag
K113Q
K89Q
K97Q
K113Q
K113Q
WT
K89Q
K97Q
K89Q
K97Q
WT
WT
K113Q
K113Q
K113Q
K89Q
K97Q
K89Q
K97Q
K89Q
K97Q
WT
WT
WT
Histone 3
Ab
Flag
Ab
WT
WT
WT
K89R
K97R
K89R
K97R
K113R
K89R
K97R
K113R
K113R
WT
WT
K89R
K97R
K89R
K97R
K113R
WT
K113R
K89R
K97R
K113R
Isw1-Flag
Isw1-Flag
Isw1-Flag
Isw1-Flag
Isw1-Flag
Isw1-Flag
Samples were run and membrane were cut prior to incubation with the indicated antibody.
Red square indicates the image used in the figure and arrow points to the band of interest.
