# Supplementary material for: Interplay between acetylation and ubiquitination of imitation switch chromatin remodeler Isw1 confers multidrug resistance in Cryptococcus neoformans
Source: eLife. 2024 Jan 22;13:e85728. doi: 10.7554/eLife.85728 (PMC10834027; doi:10.7554/eLife.85728)
Supplement: Figure 6—source data 1. [file elife-85728-fig6-data1.zip › Figure 6-source data 1/Figure 6-source data 6.pptx]

## Slide 1
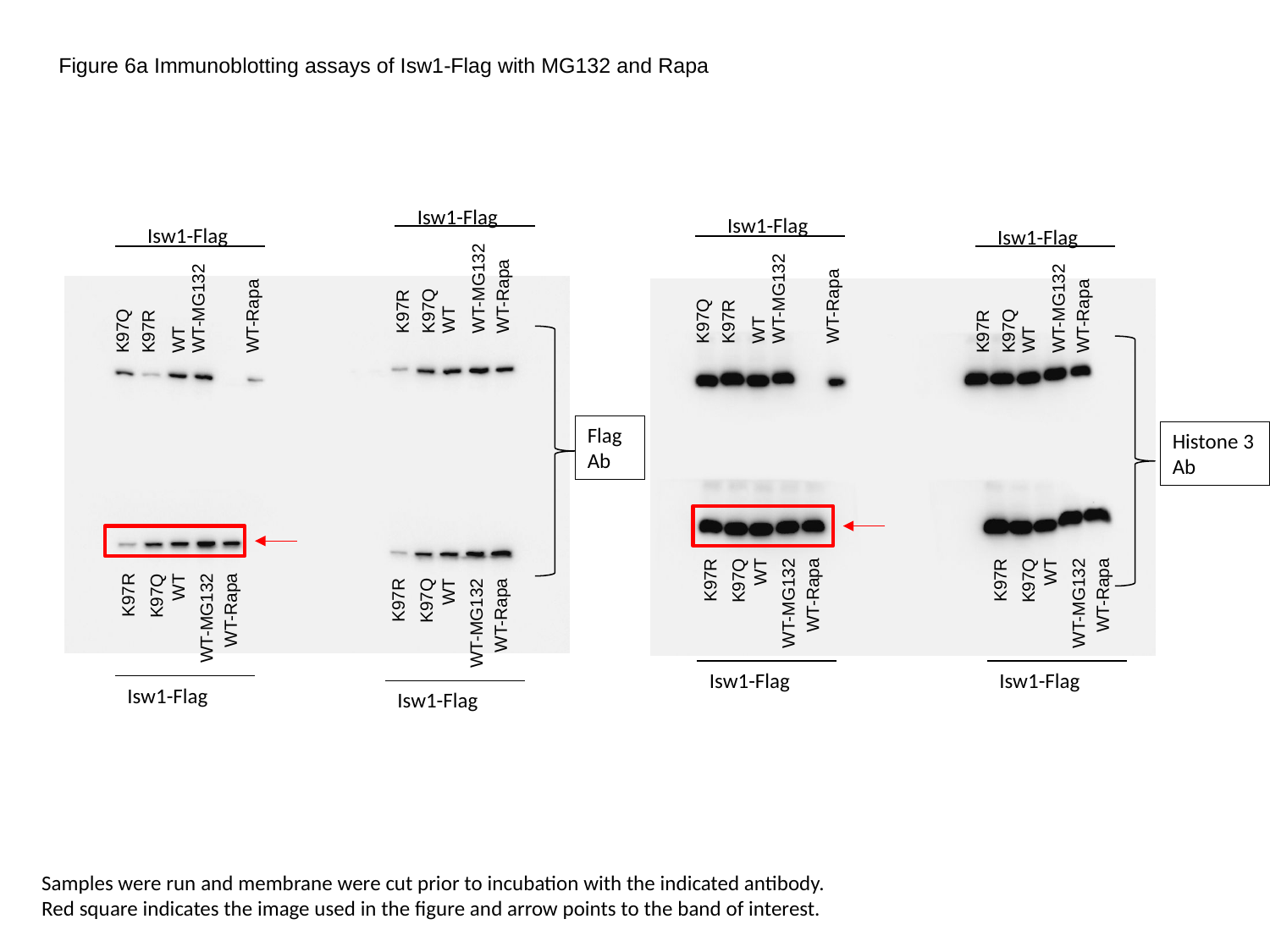

Figure 6a Immunoblotting assays of Isw1-Flag with MG132 and Rapa
Isw1-Flag
Isw1-Flag
Isw1-Flag
Isw1-Flag
WT-MG132
WT-Rapa
WT-MG132
WT-Rapa
WT-MG132
WT-Rapa
WT-MG132
WT-Rapa
K97R
K97Q
WT
K97Q
K97R
WT
K97Q
K97R
WT
K97R
K97Q
WT
Flag
Ab
Histone 3
Ab
K97R
K97Q
WT
WT-Rapa
K97R
K97Q
WT
WT-Rapa
WT-MG132
WT-MG132
K97R
K97Q
WT
WT-Rapa
WT-MG132
K97R
K97Q
WT
WT-Rapa
WT-MG132
Isw1-Flag
Isw1-Flag
Isw1-Flag
Isw1-Flag
Samples were run and membrane were cut prior to incubation with the indicated antibody.
Red square indicates the image used in the figure and arrow points to the band of interest.

## Slide 2
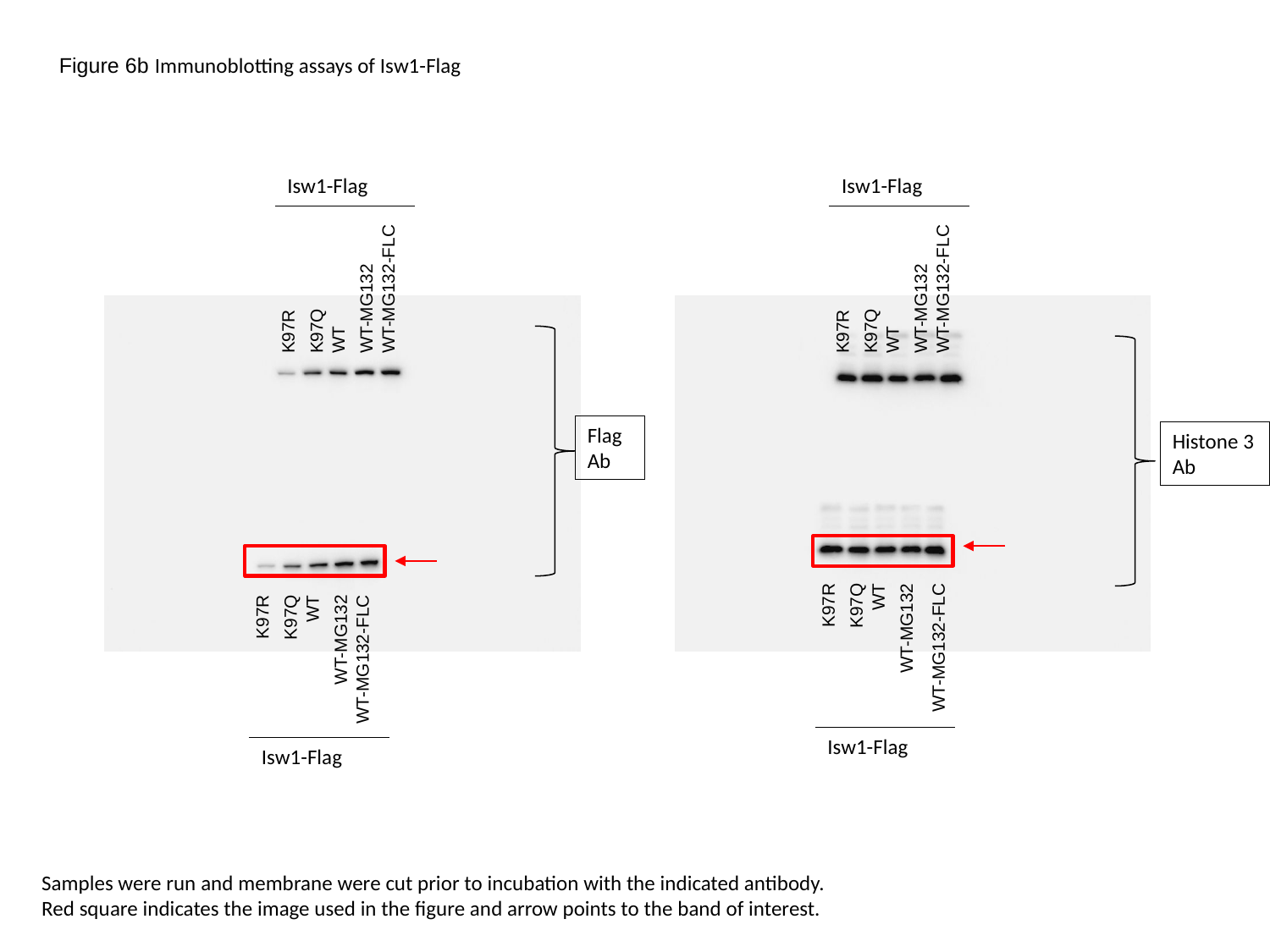

Figure 6b Immunoblotting assays of Isw1-Flag
Isw1-Flag
Isw1-Flag
WT-MG132-FLC
WT-MG132-FLC
WT-MG132
WT-MG132
K97R
K97Q
WT
K97R
K97Q
WT
Flag
Ab
Histone 3
Ab
K97R
K97Q
WT
WT-MG132
K97R
K97Q
WT
WT-MG132
WT-MG132-FLC
WT-MG132-FLC
Isw1-Flag
Isw1-Flag
Samples were run and membrane were cut prior to incubation with the indicated antibody.
Red square indicates the image used in the figure and arrow points to the band of interest.

## Slide 3
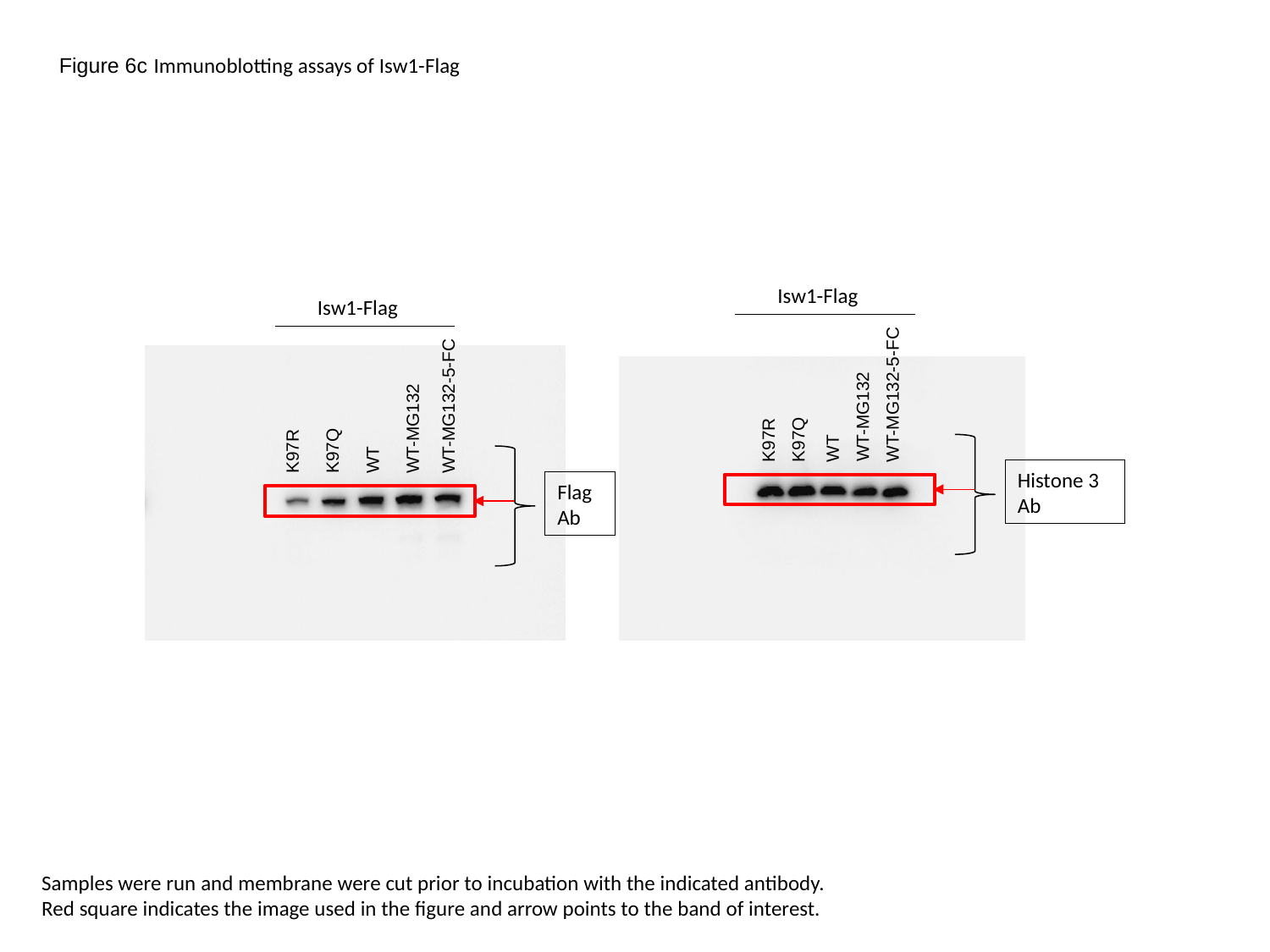

Figure 6c Immunoblotting assays of Isw1-Flag
Isw1-Flag
Isw1-Flag
WT-MG132-5-FC
WT-MG132-5-FC
WT-MG132
K97R
K97Q
WT
WT-MG132
K97R
K97Q
WT
Histone 3
Ab
Flag
Ab
Samples were run and membrane were cut prior to incubation with the indicated antibody.
Red square indicates the image used in the figure and arrow points to the band of interest.

## Slide 4
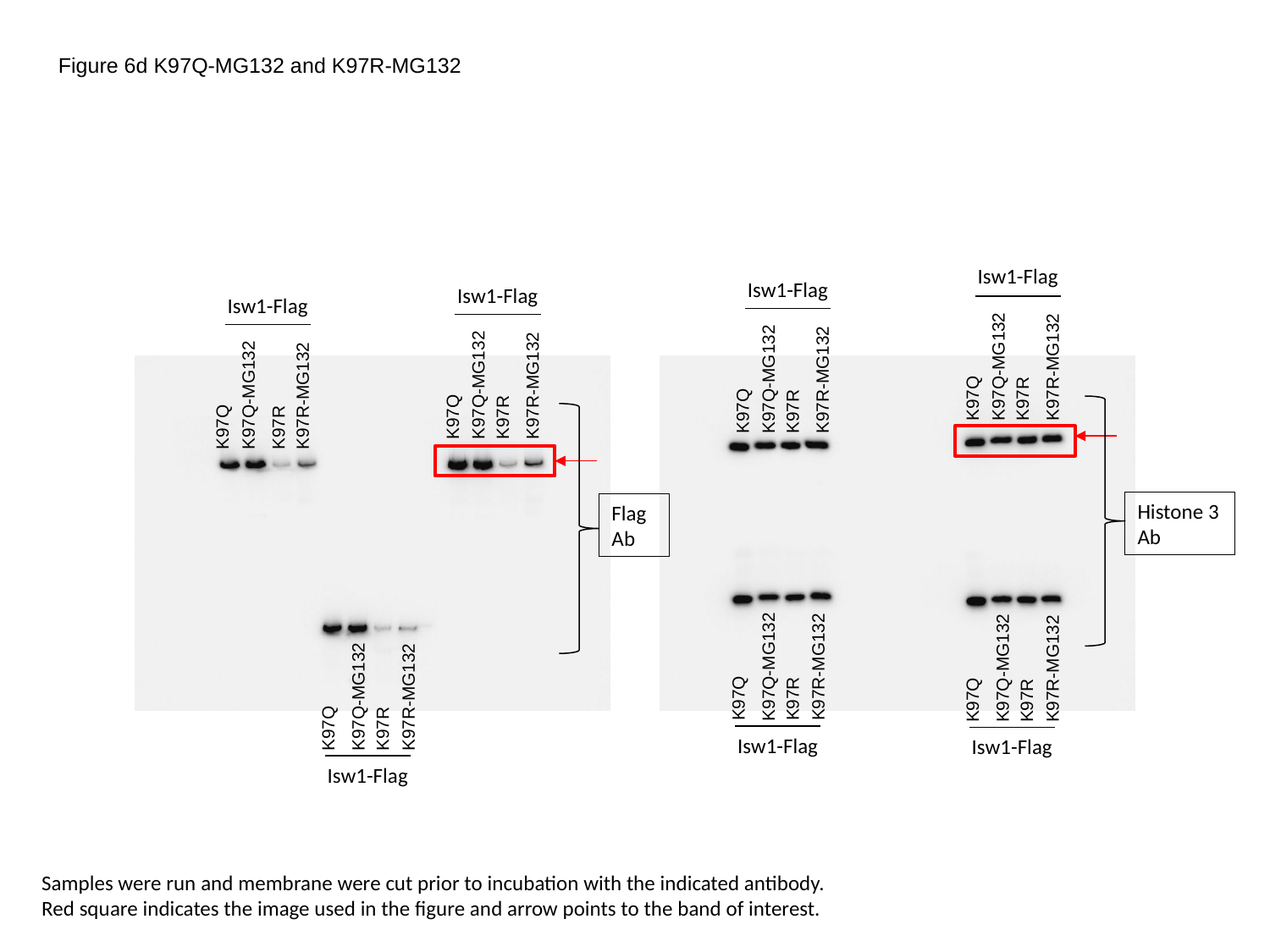

Figure 6d K97Q-MG132 and K97R-MG132
Isw1-Flag
Isw1-Flag
Isw1-Flag
Isw1-Flag
K97R-MG132
K97Q-MG132
K97R-MG132
K97Q
K97R
K97Q-MG132
K97R-MG132
K97Q-MG132
K97Q
K97R
K97R-MG132
K97Q
K97R
K97Q-MG132
K97Q
K97R
Histone 3
Ab
Flag
Ab
K97R-MG132
K97R-MG132
K97Q-MG132
K97Q-MG132
K97Q
K97R
K97Q
K97R
K97R-MG132
K97Q-MG132
K97Q
K97R
Isw1-Flag
Isw1-Flag
Isw1-Flag
Samples were run and membrane were cut prior to incubation with the indicated antibody.
Red square indicates the image used in the figure and arrow points to the band of interest.

## Slide 5
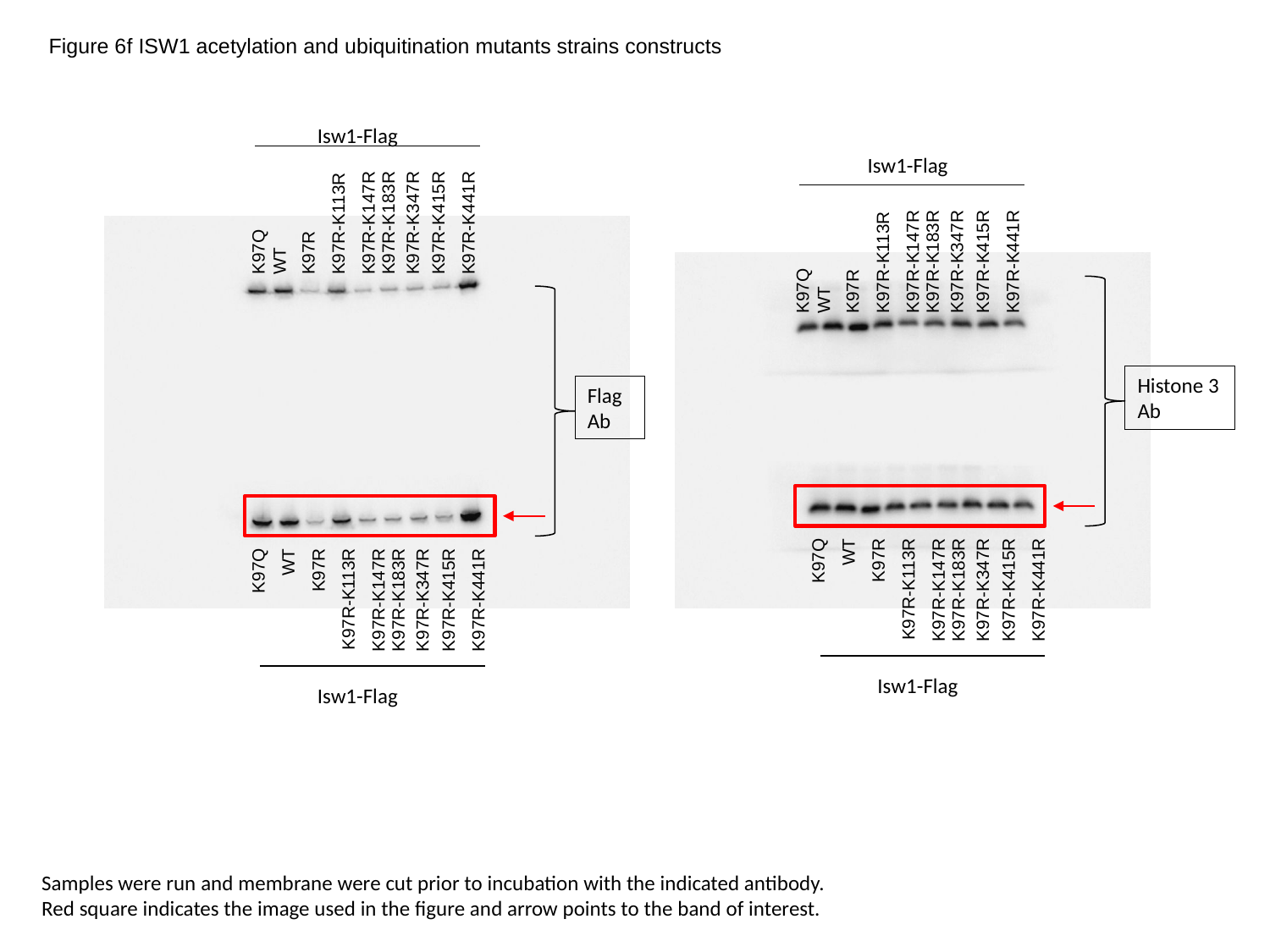

Figure 6f ISW1 acetylation and ubiquitination mutants strains constructs
Isw1-Flag
Isw1-Flag
K97R-K113R
K97R-K147R
K97R-K183R
K97R-K347R
K97R-K415R
K97R-K441R
K97Q
WT
K97R-K113R
K97R-K147R
K97R-K183R
K97R-K347R
K97R-K415R
K97R-K441R
K97R
K97Q
WT
K97R
Histone 3
Ab
Flag
Ab
K97R
K97Q
WT
K97R
K97Q
WT
K97R-K113R
K97R-K147R
K97R-K183R
K97R-K347R
K97R-K415R
K97R-K441R
K97R-K113R
K97R-K147R
K97R-K183R
K97R-K347R
K97R-K415R
K97R-K441R
Isw1-Flag
Isw1-Flag
Samples were run and membrane were cut prior to incubation with the indicated antibody.
Red square indicates the image used in the figure and arrow points to the band of interest.
