# Supplementary material for: Interplay between acetylation and ubiquitination of imitation switch chromatin remodeler Isw1 confers multidrug resistance in Cryptococcus neoformans
Source: eLife. 2024 Jan 22;13:e85728. doi: 10.7554/eLife.85728 (PMC10834027; doi:10.7554/eLife.85728)
Supplement: Figure 7—source data 1. [file elife-85728-fig7-data1.zip › Figure 7-source data 1/FIgure 7-source data 3.pptx]

## Slide 1
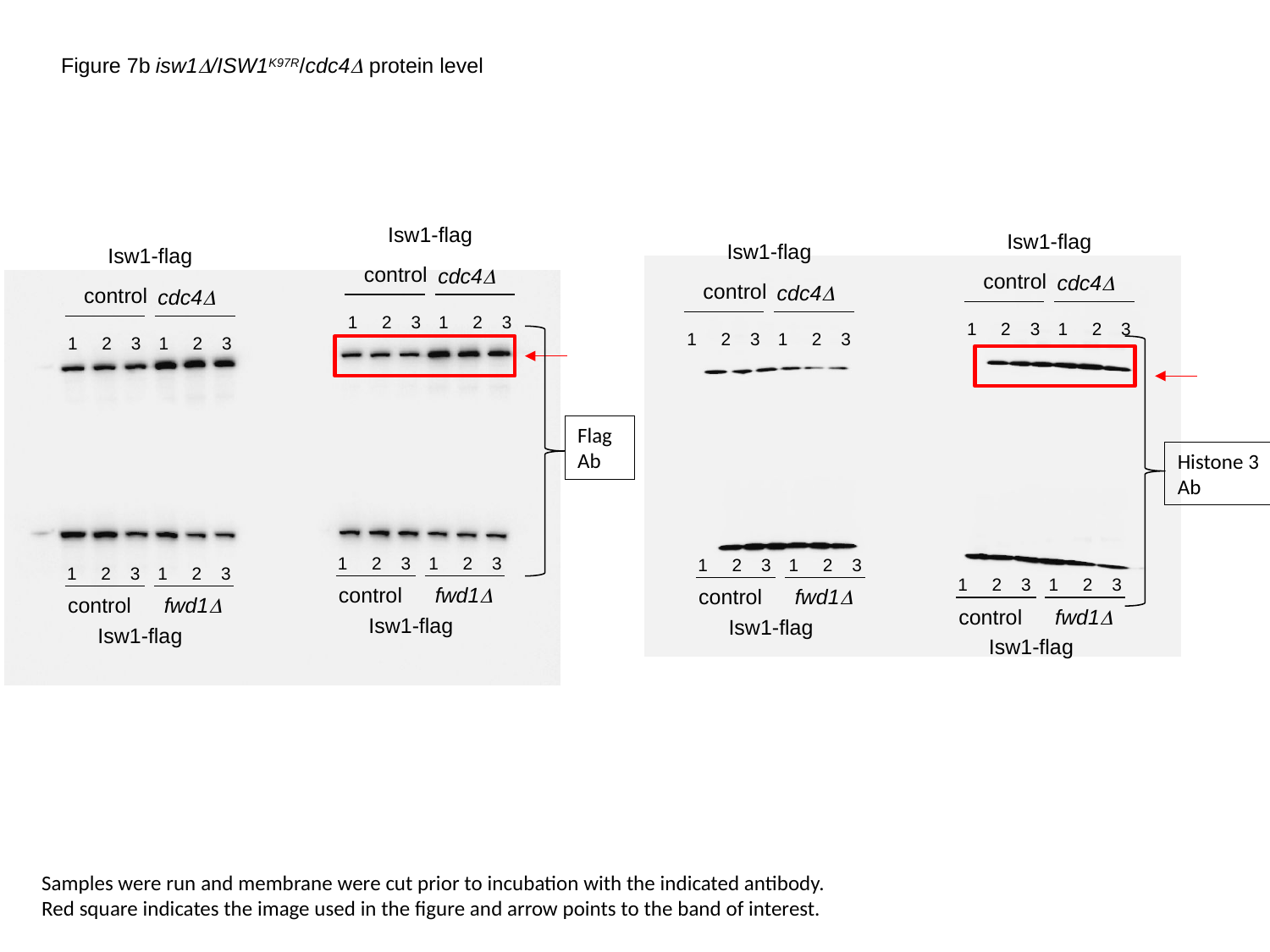

Figure 7b isw1Δ/ISW1K97R/cdc4Δ protein level
Isw1-flag
Isw1-flag
Isw1-flag
Isw1-flag
control
cdc4Δ
control
cdc4Δ
control
cdc4Δ
control
cdc4Δ
1 2 3
1 2 3
1 2 3
1 2 3
1 2 3
1 2 3
1 2 3
1 2 3
Flag
Ab
Histone 3
Ab
1 2 3
1 2 3
1 2 3
1 2 3
1 2 3
1 2 3
1 2 3
1 2 3
control
fwd1Δ
control
fwd1Δ
control
fwd1Δ
control
fwd1Δ
Isw1-flag
Isw1-flag
Isw1-flag
Isw1-flag
Samples were run and membrane were cut prior to incubation with the indicated antibody.
Red square indicates the image used in the figure and arrow points to the band of interest.

## Slide 2
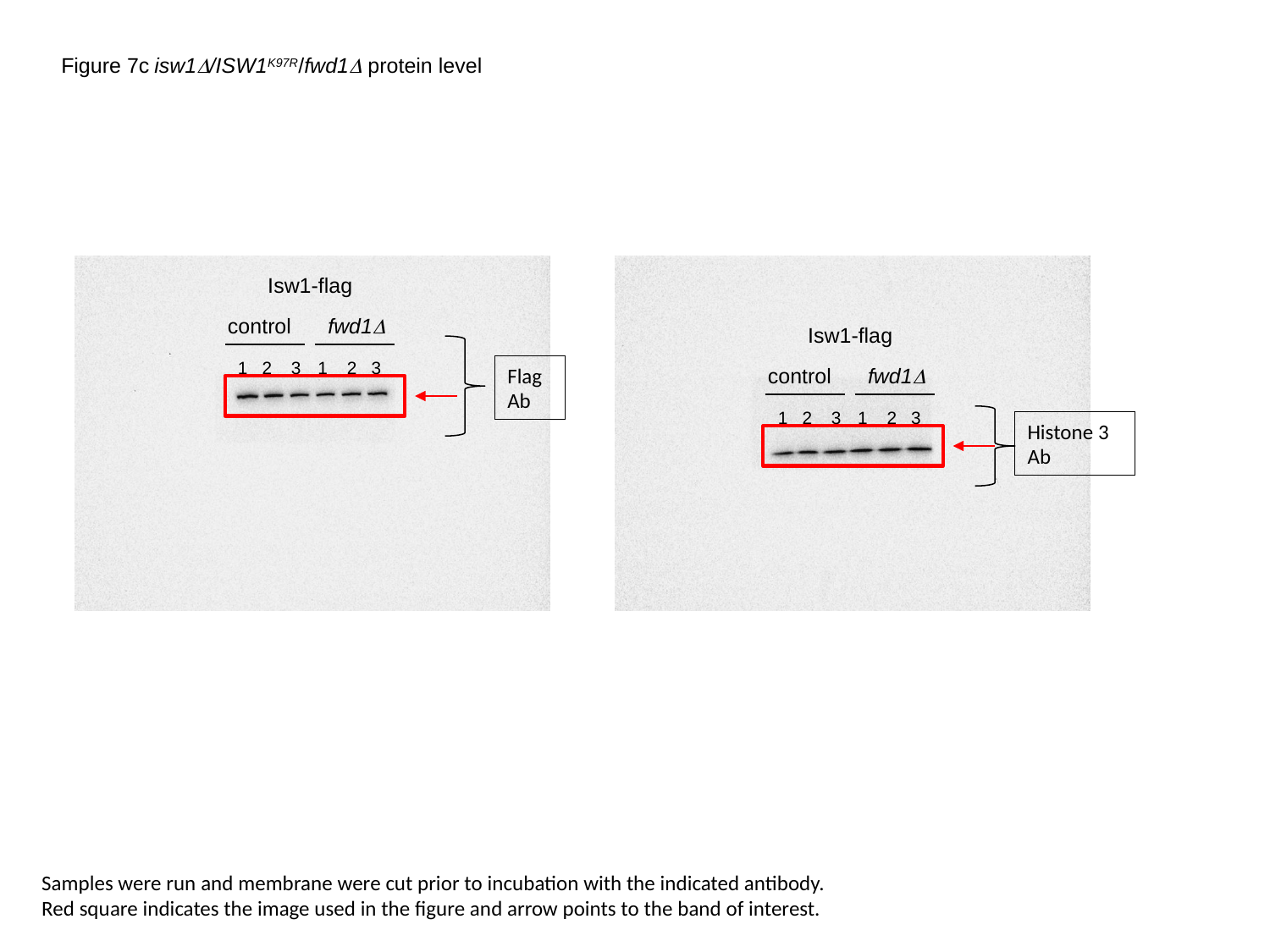

Figure 7c isw1Δ/ISW1K97R/fwd1Δ protein level
Isw1-flag
control
fwd1Δ
Isw1-flag
1 2 3
1 2 3
Flag
Ab
control
fwd1Δ
1 2 3
1 2 3
Histone 3
Ab
Samples were run and membrane were cut prior to incubation with the indicated antibody.
Red square indicates the image used in the figure and arrow points to the band of interest.

## Slide 3
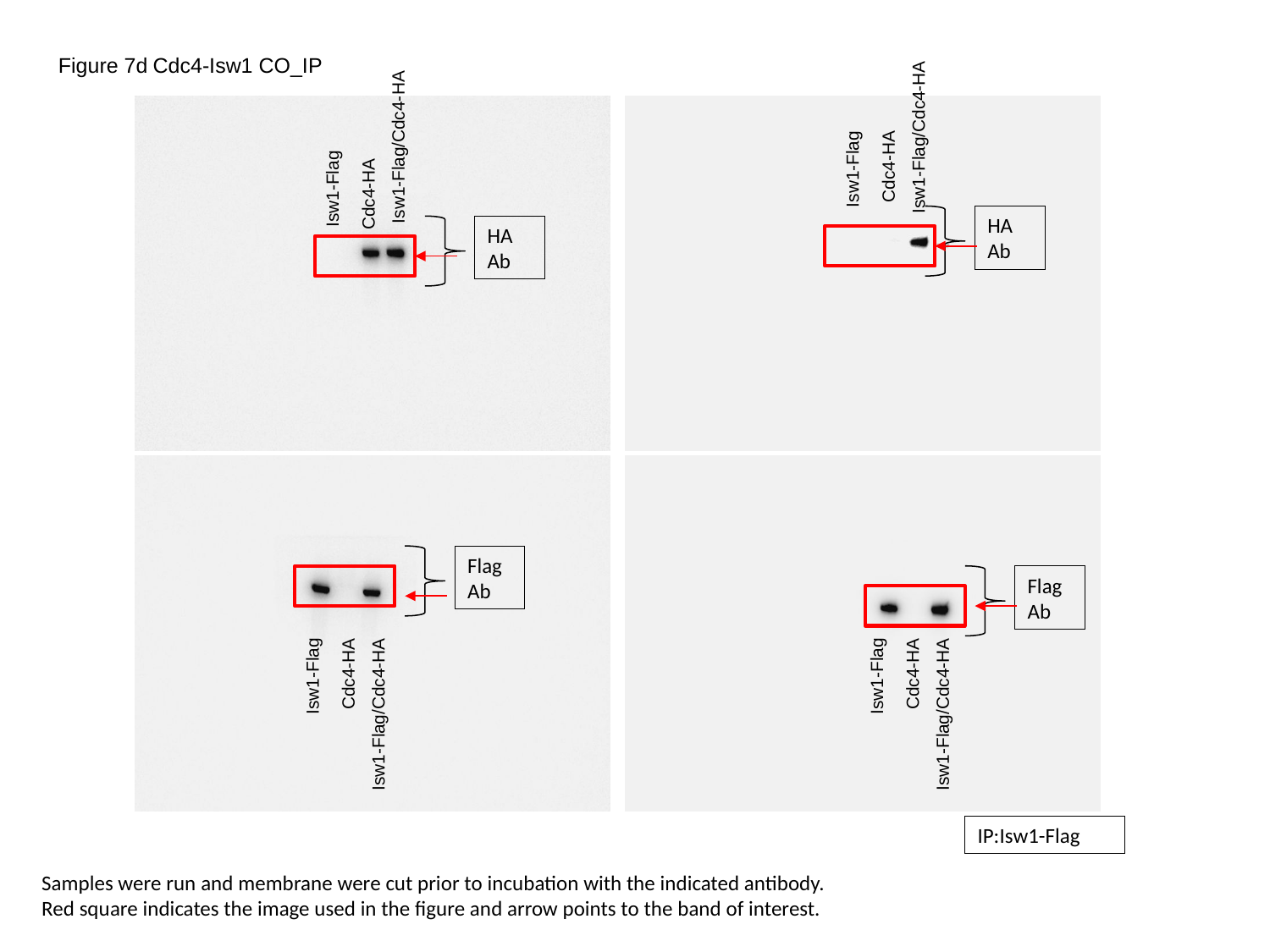

Figure 7d Cdc4-Isw1 CO_IP
Isw1-Flag/Cdc4-HA
Isw1-Flag/Cdc4-HA
Isw1-Flag
Cdc4-HA
Isw1-Flag
Cdc4-HA
HA
Ab
HA
Ab
Flag
Ab
Flag
Ab
Isw1-Flag
Cdc4-HA
Isw1-Flag
Cdc4-HA
Isw1-Flag/Cdc4-HA
Isw1-Flag/Cdc4-HA
IP:Isw1-Flag
Samples were run and membrane were cut prior to incubation with the indicated antibody.
Red square indicates the image used in the figure and arrow points to the band of interest.

## Slide 4
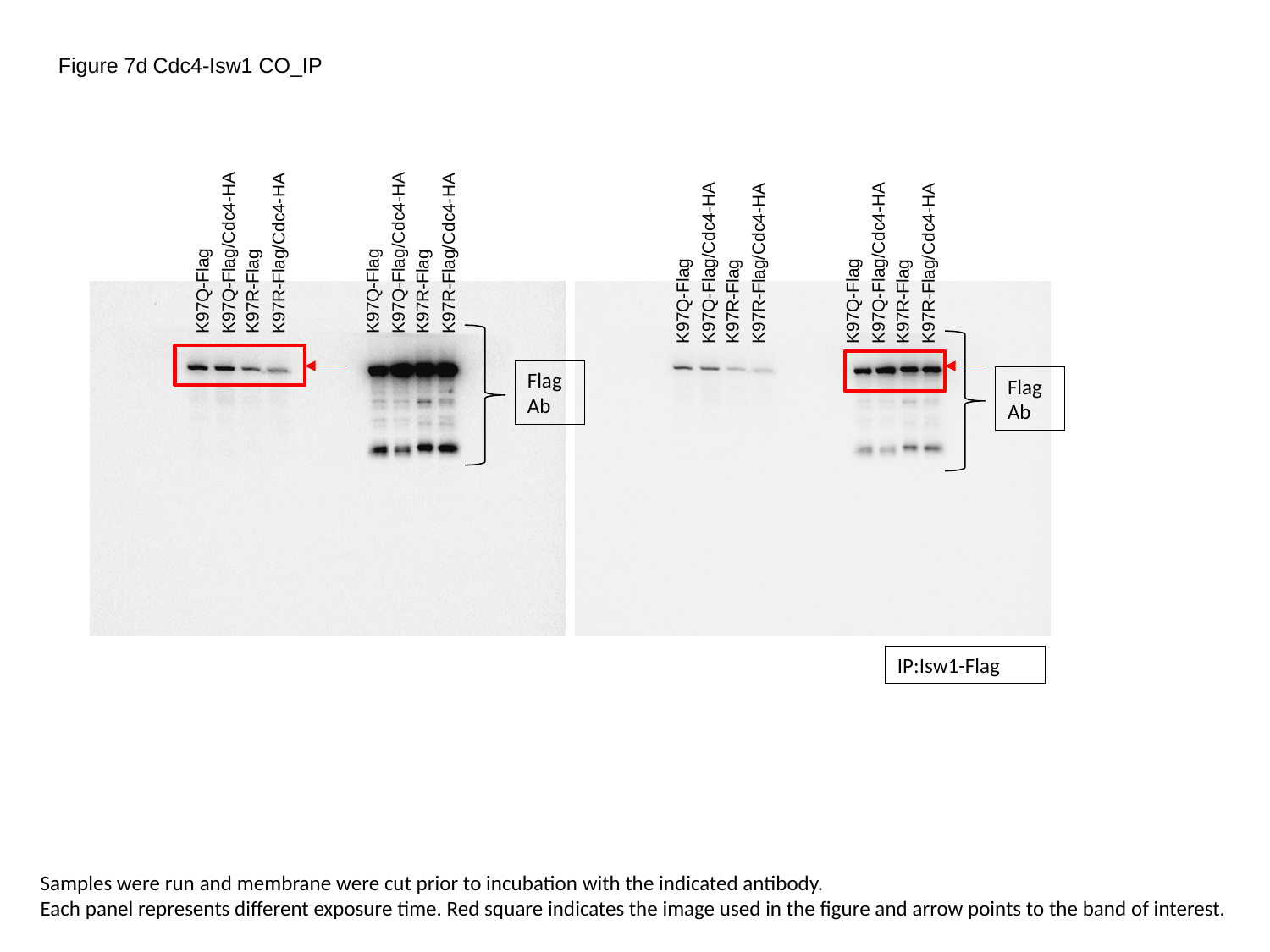

Figure 7d Cdc4-Isw1 CO_IP
K97Q-Flag/Cdc4-HA
K97R-Flag/Cdc4-HA
K97Q-Flag/Cdc4-HA
K97R-Flag/Cdc4-HA
K97Q-Flag/Cdc4-HA
K97R-Flag/Cdc4-HA
K97Q-Flag/Cdc4-HA
K97R-Flag/Cdc4-HA
K97Q-Flag
K97R-Flag
K97Q-Flag
K97R-Flag
K97Q-Flag
K97R-Flag
K97Q-Flag
K97R-Flag
Flag
Ab
Flag
Ab
IP:Isw1-Flag
Samples were run and membrane were cut prior to incubation with the indicated antibody.
Each panel represents different exposure time. Red square indicates the image used in the figure and arrow points to the band of interest.

## Slide 5
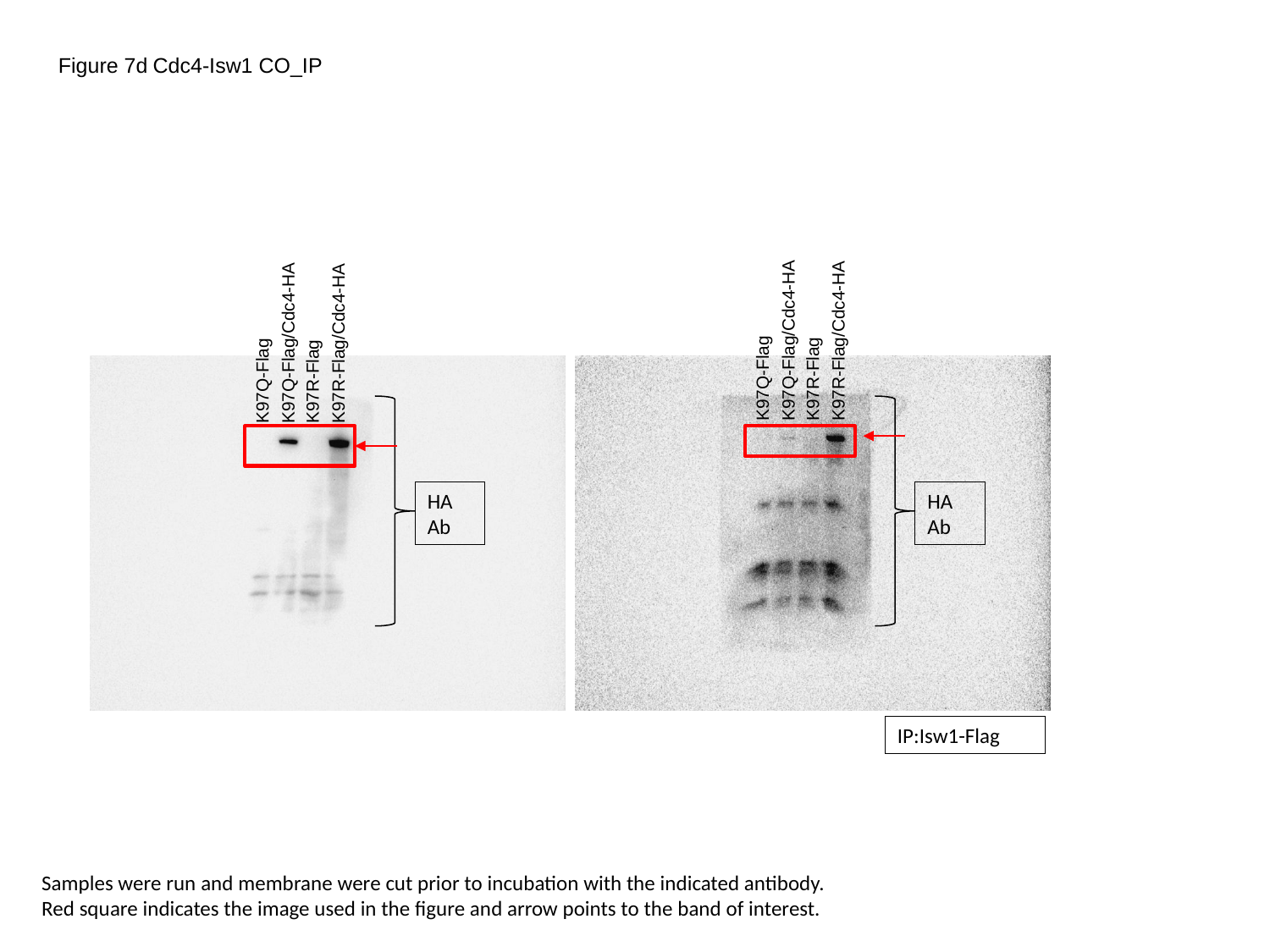

Figure 7d Cdc4-Isw1 CO_IP
K97Q-Flag/Cdc4-HA
K97R-Flag/Cdc4-HA
K97Q-Flag/Cdc4-HA
K97R-Flag/Cdc4-HA
K97Q-Flag
K97R-Flag
K97Q-Flag
K97R-Flag
HA
Ab
HA
Ab
IP:Isw1-Flag
Samples were run and membrane were cut prior to incubation with the indicated antibody.
Red square indicates the image used in the figure and arrow points to the band of interest.
