# Supplementary material for: Interplay between acetylation and ubiquitination of imitation switch chromatin remodeler Isw1 confers multidrug resistance in Cryptococcus neoformans
Source: eLife. 2024 Jan 22;13:e85728. doi: 10.7554/eLife.85728 (PMC10834027; doi:10.7554/eLife.85728)
Supplement: Figure 8—source data 1. [file elife-85728-fig8-data1.zip › Figure 8-source data 1/Figure 8-source data 7.pptx]

## Slide 1
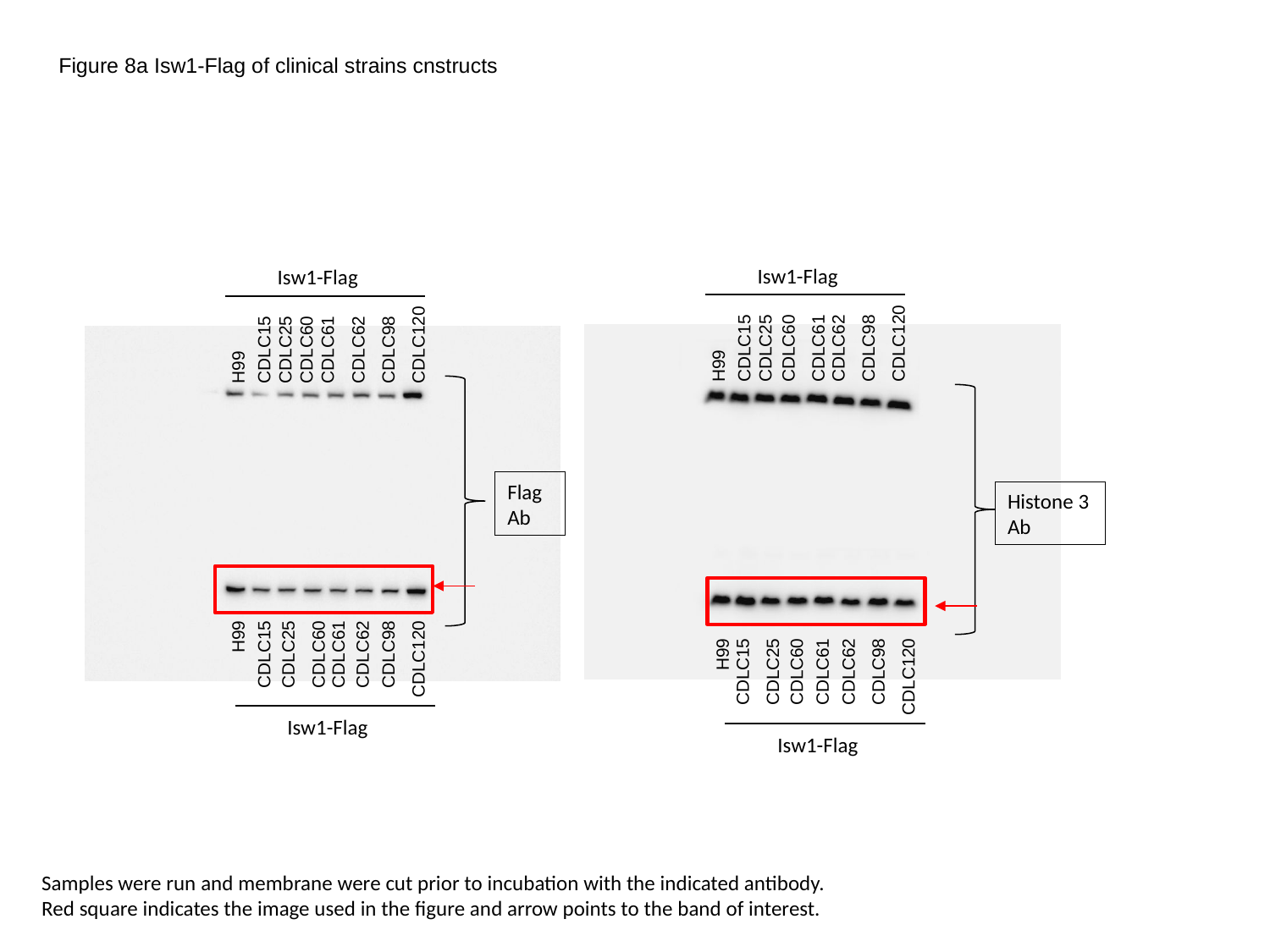

Figure 8a Isw1-Flag of clinical strains cnstructs
Isw1-Flag
Isw1-Flag
H99
CDLC15
CDLC25
CDLC60
CDLC61
CDLC62
CDLC98
CDLC120
H99
CDLC15
CDLC25
CDLC60
CDLC61
CDLC62
CDLC98
CDLC120
Flag
Ab
Histone 3
Ab
H99
CDLC15
CDLC25
CDLC60
CDLC61
CDLC62
CDLC98
CDLC120
H99
CDLC15
CDLC25
CDLC60
CDLC61
CDLC62
CDLC98
CDLC120
Isw1-Flag
Isw1-Flag
Samples were run and membrane were cut prior to incubation with the indicated antibody.
Red square indicates the image used in the figure and arrow points to the band of interest.

## Slide 2
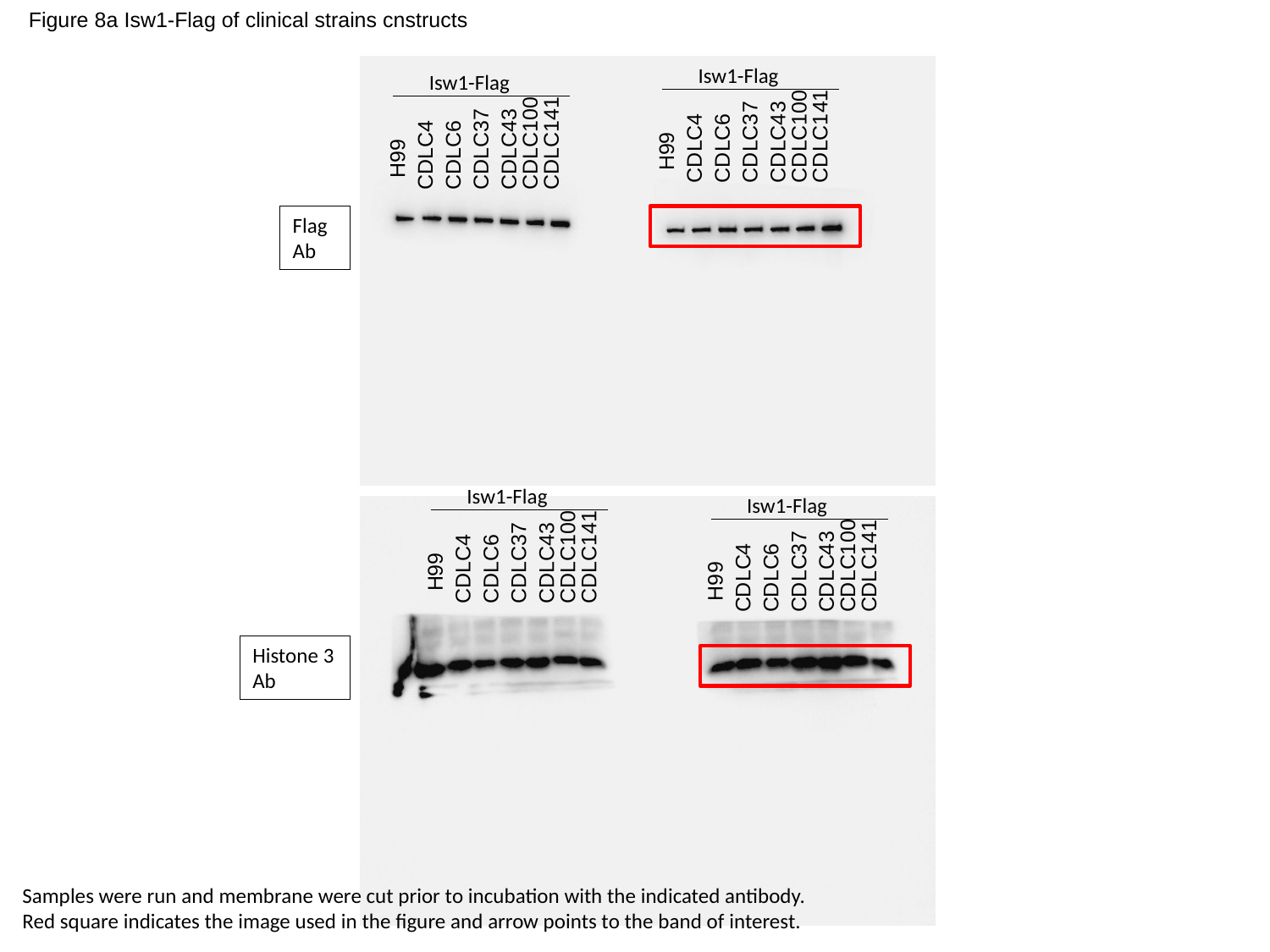

Figure 8a Isw1-Flag of clinical strains cnstructs
Isw1-Flag
Isw1-Flag
CDLC100
CDLC141
CDLC100
CDLC141
CDLC37
CDLC43
CDLC37
CDLC43
CDLC4
CDLC6
CDLC4
CDLC6
 H99
 H99
Flag
Ab
Isw1-Flag
Isw1-Flag
CDLC100
CDLC141
CDLC100
CDLC141
CDLC37
CDLC43
CDLC37
CDLC43
CDLC4
CDLC6
CDLC4
CDLC6
 H99
 H99
Histone 3
Ab
Samples were run and membrane were cut prior to incubation with the indicated antibody.
Red square indicates the image used in the figure and arrow points to the band of interest.

## Slide 3
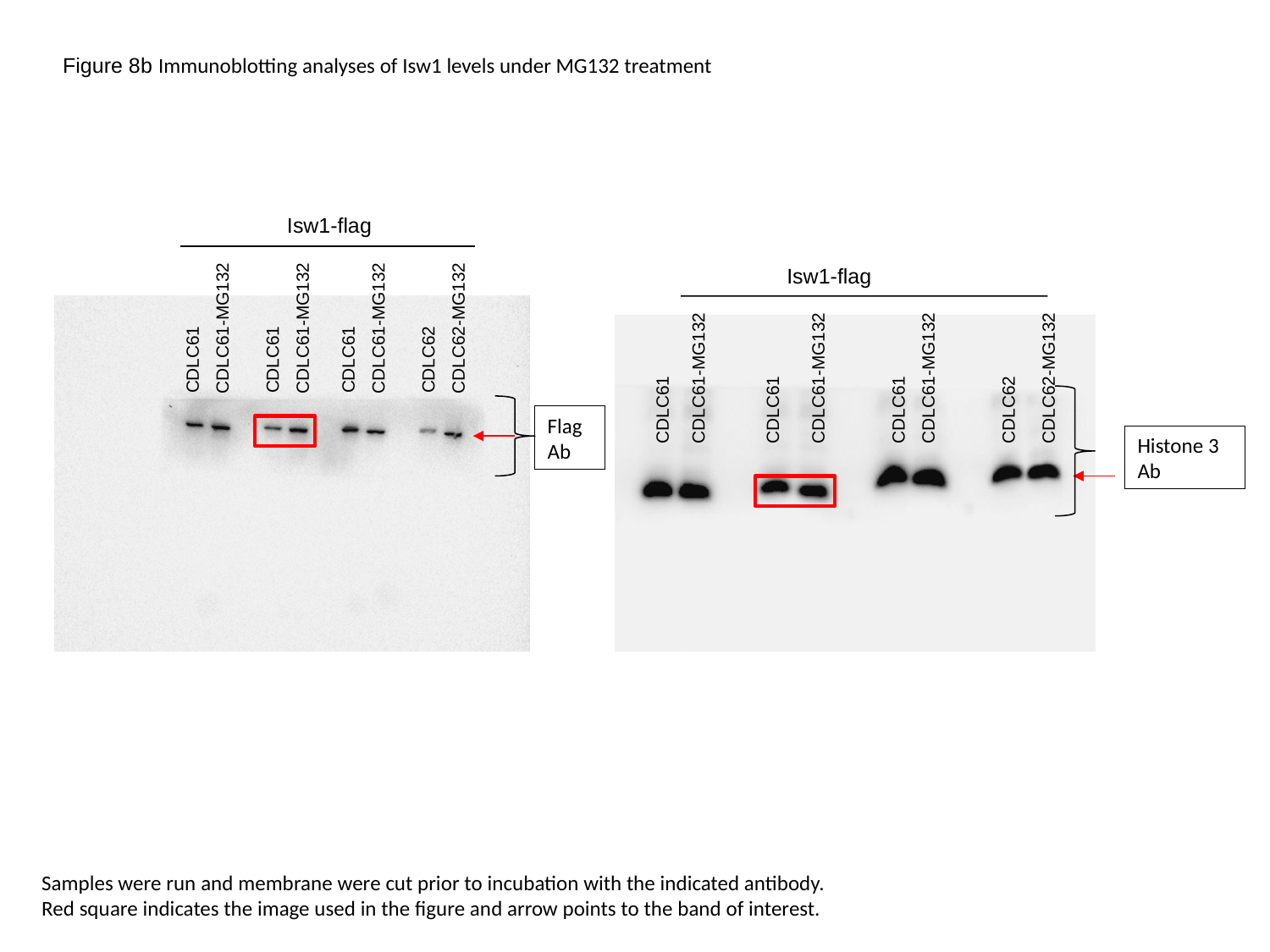

Figure 8b Immunoblotting analyses of Isw1 levels under MG132 treatment
Isw1-flag
Isw1-flag
CDLC61-MG132
CDLC61-MG132
CDLC61-MG132
CDLC62-MG132
CDLC61
CDLC61
CDLC61
CDLC62
CDLC61-MG132
CDLC61-MG132
CDLC61-MG132
CDLC62-MG132
CDLC61
CDLC61
CDLC61
CDLC62
Flag
Ab
Histone 3
Ab
Samples were run and membrane were cut prior to incubation with the indicated antibody.
Red square indicates the image used in the figure and arrow points to the band of interest.

## Slide 4
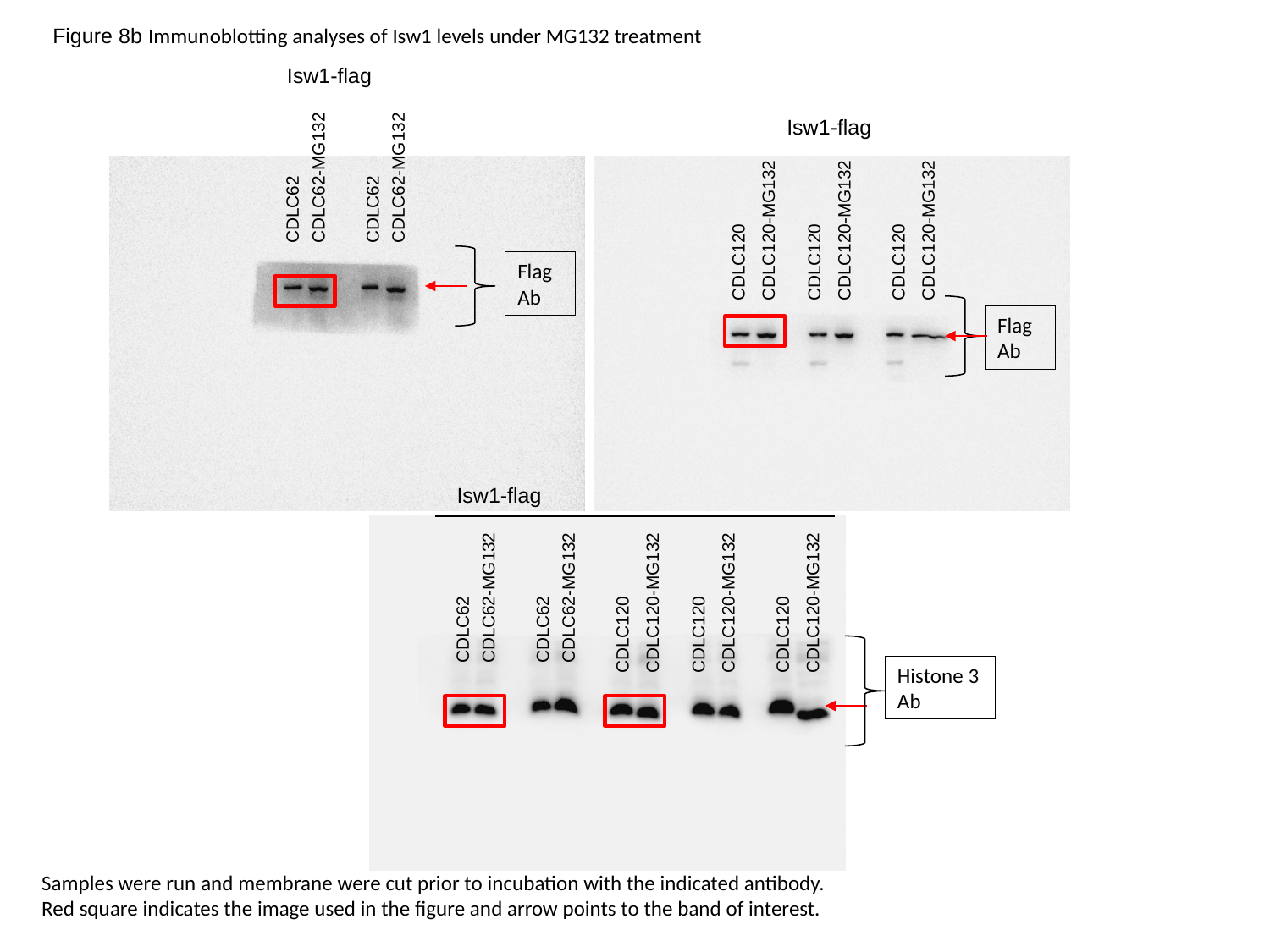

Figure 8b Immunoblotting analyses of Isw1 levels under MG132 treatment
Isw1-flag
Isw1-flag
CDLC62-MG132
CDLC62-MG132
CDLC62
CDLC62
CDLC120-MG132
CDLC120-MG132
CDLC120-MG132
CDLC120
CDLC120
CDLC120
Flag
Ab
Flag
Ab
Isw1-flag
CDLC120-MG132
CDLC120-MG132
CDLC62-MG132
CDLC62-MG132
CDLC120-MG132
CDLC62
CDLC62
CDLC120
CDLC120
CDLC120
Histone 3
Ab
Samples were run and membrane were cut prior to incubation with the indicated antibody.
Red square indicates the image used in the figure and arrow points to the band of interest.

## Slide 5
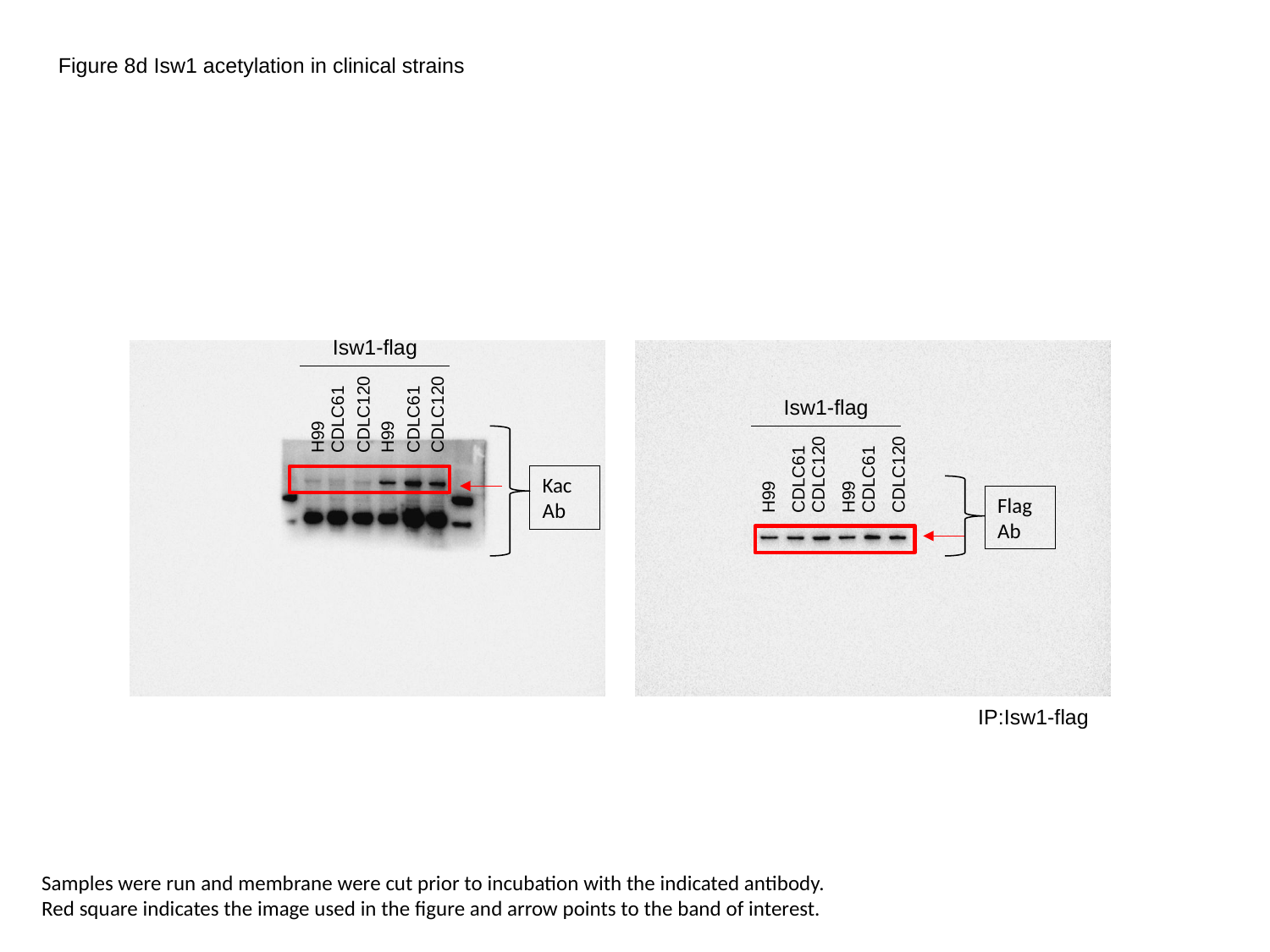

Figure 8d Isw1 acetylation in clinical strains
Isw1-flag
Isw1-flag
H99
CDLC61
CDLC120
H99
CDLC61
CDLC120
H99
CDLC61
CDLC120
H99
CDLC61
CDLC120
Kac
Ab
Flag
Ab
IP:Isw1-flag
Samples were run and membrane were cut prior to incubation with the indicated antibody.
Red square indicates the image used in the figure and arrow points to the band of interest.
